# Experimental and Theoretical Insights into CO_2_ and N_2_ Capture Using Natural Hydrophobic Deep Eutectic Solvents at High Pressures

**DOI:** 10.1021/acsomega.5c01533

**Published:** 2025-06-04

**Authors:** Ahmad Al-Bodour, Noor Alomari, Shan Khai Liew, Santiago Aparicio, James Springstead, Mert Atilhan

**Affiliations:** † Chemical and Paper Engineering Department, 4175 Western Michigan University, 4601 Campus Dr. Floyd Hall, Kalamazoo, Michigan 49008-5462, United States; ‡ Department of Chemistry, 16725 University of Burgos, Plaza Misael Bañuelos s.n., Burgos 09001, Spain

## Abstract

This study explores three binary natural hydrophobic deep eutectic solvents (HDESs) for capturing carbon dioxide (CO_2_) and nitrogen (N_2_) at high pressures. The HDES systems, comprising linoleic acid (LnA) as a hydrogen-bond donor (HBD) and camphor (CAM), citral (CIT), or piperitone (PIP) as a hydrogen-bond acceptor (HBA), were synthesized and characterized for density, viscosity, conductivity, surface tension, and contact angle. High-pressure gas absorption experiments demonstrated CO_2_ and N_2_ capture, achieving absorption rates of ∼62%–92% within 100 s at 10–30 bar. At 25 bar, a mole fraction absorption of 0.47 matched the performance of aqueous monoethanolamine (MEA) at 25 °C. Among the HDESs, CAM–LnA (1:1) exhibited the highest CO_2_ selectivity at 2.5 and 5 bar, with values of 41.4 and 44.2, respectively. The conductor-like screening model for real solvents (COSMO-RSs) method predicted eutectic points and gas absorption, while molecular dynamics simulations assessed gas interactions at the molecular level. The results underscore the potential of HDES for high-pressure gas capture, providing insights into their production, characterization, and applications.

## Graphical abstract

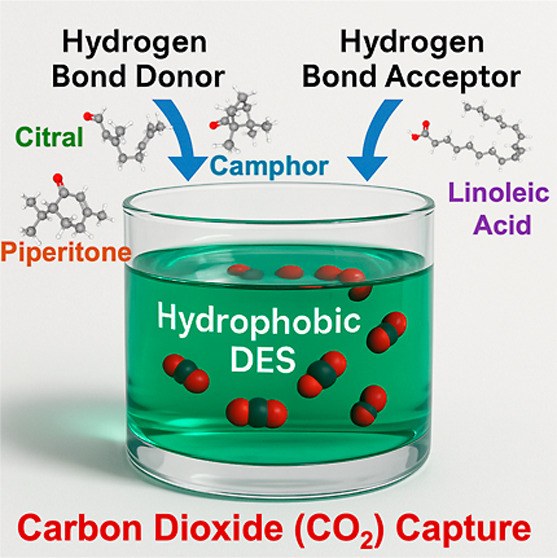

## 1 Introduction

The increasing industrial energy demands and continuous fossil fuel consumption have led to an approximate 1% annual rise in atmospheric carbon dioxide (CO_2_) concentrations over the past decade.^1,2^ Significantly, CO_2_ gas emissions contribute to the global warming problem.^3,4^ The concentration of CO_2_ gas is around 10–15% from the flue gas mixture.^5,6^ These emissions pose significant threats to both the environment and human health. Consequently, reducing CO_2_ emissions through sequestration and capture has become a top priority for both industry and academia.

Over the history, the aqueous solutions of alkanolamine have been utilized broadly for the purpose of CO_2_ gas capture.^7,8^ These amines have a high performance, in terms of CO_2_ absorption and the rate of gas capture. However, these solutions experience various drawbacks involving corrosiveness,^9^ toxicity,^10^ high regeneration energy requirements,^11–13^ and poor degradation behavior.^14^ Hence, it is necessary to produce high-capacity and low-volatility absorbents, which can be realistically a replacement for the traditional existing amines. Recently, attention has shifted to a class of materials known as deep eutectic solvents (DESs). These novel solvents have shown a significant potential for CO_2_ capture. DES systems are created by combining a hydrogen-bond acceptor (HBA) with a hydrogen-bond donor (HBD), resulting in a homogeneous liquid phase formed through hydrogen bonding.^15,16^ The moisture content of the flue gas mixture is a critical factor to consider in the CO_2_ capture. Normally, it represents 8–20% of the effluent gas volume.^17^ This amount of moisture drastically reduces the CO_2_ absorption capacity when hydrophilic liquids are used in the absorption process. The absorption capacity reduction leads to a higher operational cost and a higher consumption of energy.^18^ Consequently, interest has shifted toward exploring hydrophobic DES systems (HDES) for the purpose of CO_2_ capture. This type of systems was introduced for the first time by Osch’s group in 2015.^19^ HDES systems have been exploited for the process of gas separation^20^ and liquid–liquid extraction.^21^ For in-depth details on the recent gas absorption applications of DES/or natural deep eutectic solvents (NADESs) and the various experimental designs for gas absorption by DES/NADES, we refer readers to our recent systematic review paper,^22^ which provides a detailed and extensive examination of this area. This study investigates the performance of three monoterpenoid-based hydrophobic natural deep eutectic solvents for CO_2_ capture. These systems are classified as type-V DES, the latest development in DES technology.^23^ The HDESs prepared in this study were formed by combining camphor (CAM), citral (CIT), and piperitone (PIP) as hydrogen-bond acceptors (HBAs) and linoleic acid (LnA) as the hydrogen-bond donor (HBD). Camphor is a hydrophobic natural material that is utilized in different areas such as a preservative component in cosmetics and pharmaceuticals, embalming fluid, and moth repellant.^24^ Also, citral is a hydrophobic^25^ natural compound identified as a safe material by the U.S. Food and Drug Administration (FDA). Usually, it is utilized as a food additive and a flavor in addition to other applications as a precursor for aromatics production.^26^ Piperitone is natural derived material from plants, which is presented commercially as an additive for food.^27^ Due to the aforementioned characteristics and uses of CAM, CIT, and PIP, they were chosen to be the HBA components. In addition to the higher degree of hydrophobicity, linoleic acid as HBD provides a complementary environmental sustainability aim of producing HDES systems because it is widely used, biodegradable, and nontoxic material.^28^ This introduces them as perfect candidates as ecologically friendly solvents. The system of CAM–LnA was prepared for the first by our lab but for water treatment application^29^ and here it is utilized for a different application. To our knowledge, the other two systems were prepared and reported for the first time in this work. The resulting HDES systems—CAM–LnA, CIT–LnA, and PIP–LnA—were mixed in a 1:1 molar ratio. The CO_2_ capture performance was evaluated under near-real-life conditions, encompassing both postcombustion and precombustion stages, with a particular focus on high-pressure scenarios. The conductor-like screening model for real solvents (COSMO-RSs), a quantum chemical calculation method developed by Klamt,^30^ was employed to predict the solubility of CO_2_ gas in the three systems. COSMO-RS is based on individual species unimolecular quantum chemical calculations. It is broadly employed for the fluid’s thermodynamics property predictions, including gas absorption.^31^ Comparing experimental data with the COSMO-RS predictions offers a comprehensive understanding of the high-pressure absorption kinetics and characteristics of the prepared HDES systems. This provides valuable insights into sustainable CO_2_ capture technology.

We also investigated the solubilities of CO_2_ and N_2_ in DES at the nanoscopic level using Molecular Dynamics (MD) simulations to evaluate their interaction energies. The electronic structure of the hydrophobic NADES systems was examined through quantum mechanical modeling, providing detailed insights into the bonding mechanisms between the HDES systems and gas molecules. Additionally, MD simulations were used to observe the time-dependent behavior of the NADES systems, analyzing their bulk phase behavior from a nanoscopic perspective. This multiscale approach, from single-molecule interactions to overall system behavior, underscores the depth and comprehensiveness of our research on CO_2_ capture.

This study combines both experimental and computational approaches to provide a comprehensive understanding of the systems’ behaviors under various conditions. By bridging the gap between experimental observations and theoretical predictions, the integration enhances the validity and reliability of the findings. Notably, this research goes beyond typical pressure conditions by investigating high-pressure scenarios, covering a wide range of feasible conditions for the capture of CO_2_. This thorough examination offers valuable insights into the CO_2_ absorption efficiency of natural HDES systems in diverse operational environments. The extensive experimental data, especially under high-pressure conditions, combined with innovative modeling methods distinguish this research as a significant contribution to sustainable CO_2_ capture technology.

To further clarify the breakthrough progress of our work, we highlight that the HDES systems presented here offer several advantages over conventional amine-based CO_2_ capture solvents. The primary benefit is the reduction in operational cost due to the simplified regeneration process, as it is based on physisorption rather than chemisorption. This allows for the less energy-intensive recovery of CO_2_. Additionally, unlike traditional amines, which are known for their toxicity and corrosion, the HDES systems we introduce are nontoxic and biodegradable. Thus, this work not only proposes a more economical approach but also contributes to environmental sustainability by providing a safer alternative for large-scale CO_2_ capture operations.

## 2 Materials and Methods

### 2.1 Chemicals

For this research work, a 99.999% purity research-grade CO_2_ gas was provided by the Airgas company. A 96% purity camphor (CAM) [CAS: 76-22-2] and a 95% purity citral (CIT) [CAS: 5392-40-5] were gotten from Alfa Aesar company. Piperitone (PIP) [CAS RN: 89-81-6] with a >94% purity and Linoleic acid (LnA) [CAS RN: 60-33-3] with a purity ≥85% were collected from the TCI America company. During this work, all the collected chemicals were involved as received and without any other purifications.

### 2.2 NADES Preparation

In this study, three binary natural hydrophobic deep eutectic solvent (HDES) systems were prepared. The systems were produced by combining LnA as the HBD and the other materials (CAM, CIT, and PIP) as HBAs. The eutectic composition and eutectic temperature predictions were acquired through the COSMO-RS method. The predicted data by COSMO-RS are shown in Figure 1.

**Figure 1:**
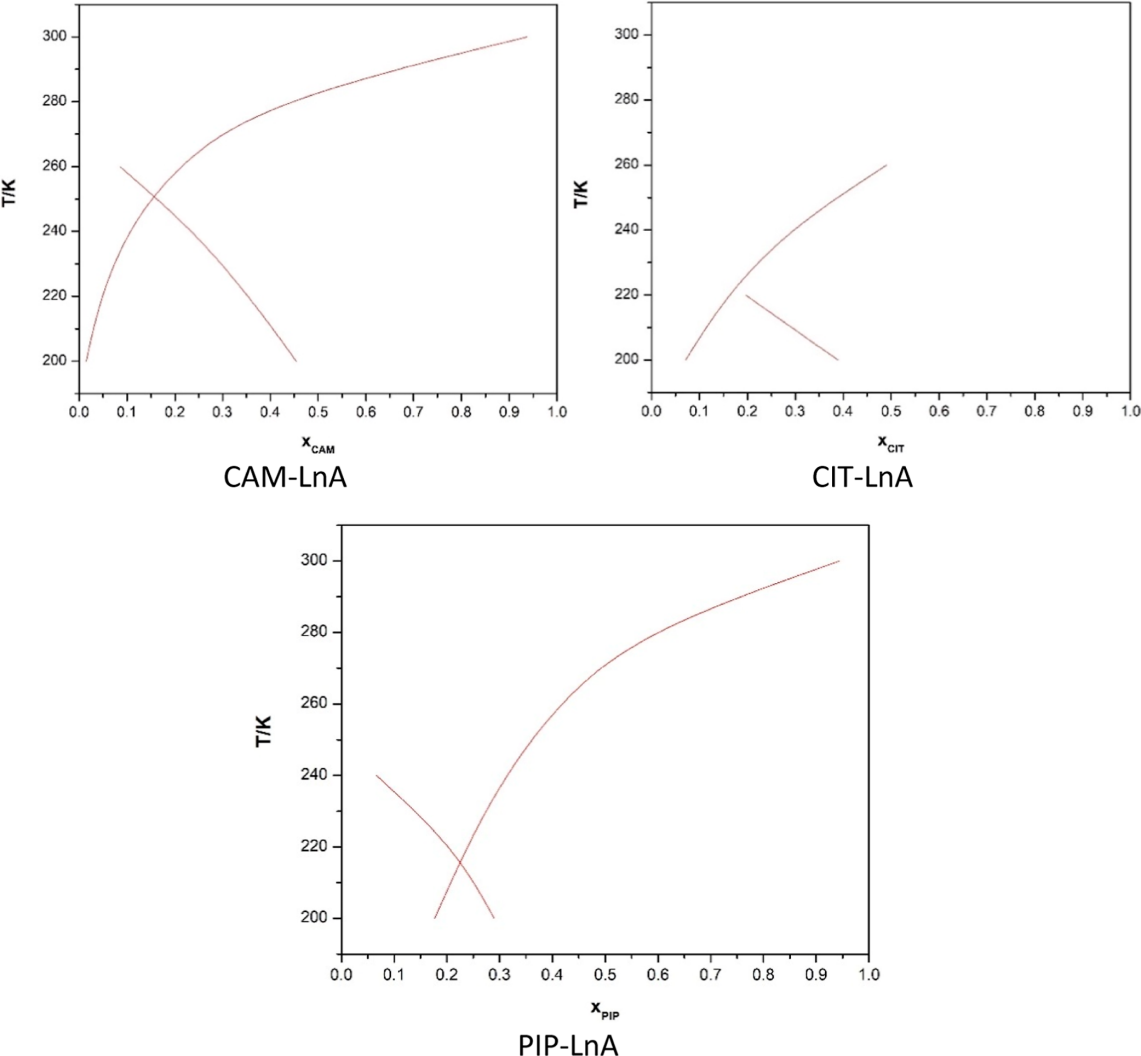
COSMO-RS eutectic composition and eutectic temperature predictions.

Molar ratios for the HDES systems were predicted as 1:5.67 for the CAM–LnA system, 1:7.25 for the CIT–LnA system, and 1:3 for the PIP–LnA system. Nonetheless, a molar mixing ratio of 1:1 could yield DES systems in the laboratory. After trying the 1:1 experiments, we observed the formation of a homogeneous liquid phase at room temperature, and the low glass transition temperatures (*T*_0_) along with FTIR spectra confirmed the physical mixing characteristic of eutectic solvents, so we used this ratio for further experiments in this work. After performing the experiments, the predicted eutectic temperatures were obtained as 250 K for the CAM–LnA system, 225 K for CIT–LnA, and 215 K for PIP–LnA DES. Based on that, the natural HDES systems were prepared at room temperature in the fume hood with rigorous mixing for 1 h using a magnetic stirrer mixer.

### 2.3 High-Pressure Gas-Solubility Experiments

Gas capture experiments were executed for carbon dioxide, CO_2_ and nitrogen N_2_ gases. Absorption work was performed by a designed and constructed in-house isochoric high-pressure gas sorption apparatus with an uncertainty of ±0.20%. The high-pressure equilibrium cell (isochoric high-pressure autoclave) was bought from Parr Instruments (Model No. 4790-HP-100 mL-T-SS-VGR-5000-BTS). This cell is built using a T316 stainless steel material and has a fixed 100 mL volume. The maximum working pressure limit is 5000 psi (345 bar), and the maximum working temperature is 350 °C. A pressure transducer Ashcroft T2 (PT, total error band accuracy TEB = ±1% of span) is linked at the top of the equilibrium cell. Also, at the top of the closure flange, a thermowell is positioned allowing the resistance temperature detector (RTD) to go all the way down to the position where the specimen is stationed inside the high-pressure cell. An entirely automated controller is hooked up to the cell (Parr Instruments Model 4838) to monitor and record the pressure and temperature second by second. The PT and the RTD are coupled to the 4838 control box module, which is linked to a computer to log the data. An exterior constant-temperature circulator bath (Polyscience SD07R-20-A11B) is employed to control the autoclave temperature by circulating the heat transfer fluid through the coils around the autoclave. The coil, autoclave flange closure and bottom, the lines, and their connections that run from and to the circulator are insulated to maintain the thermal stability. The equilibrium cell has two valves, one for gas charging and one for gas purging. Additionally, it is provided with a rupture disc for safety objects. In this setup, the cylinders of the gases are connected to gas regulators to manage the delivered pressure to the equipment. Each connecting line of the gas cylinders is isolated with a valve to prevent any contamination between the gases. After the isolation valve, a digital gauge of the pressure (GE-DPI Druck 104) is attached to the system. This is used to control the gas pressure that would be charged into the cell. In front of the pressure gauge, a hand screw manual gas pressure booster (High Pressure Company model HIP-87-6-5) is linked, which is used to pressurize and charge the gas to the cell. This gas pressure generator is controlled by its known volume and wheel revolutions (total 60 and 84 revolutions [5 mL per 7 revolutions]). The gas pressure generator temperature is controlled by a dedicated external constant temperature circulator (Polyscience SD07R-20-A11B). The circulating heat transfer fluid runs through a coil around the gas pressure booster. The inlet and outlet temperatures of the pressure booster are observed and recorded by thermocouples of the J-type. A data acquisition card (MC Measurement Computing, USB-TEMP) is connected to the thermocouples, and the card is connected to a computer with specific software recording the data. The connecting line between the gas pressure generator and the cell has a metering valve for fine gas flow control to the autoclave. A Welch CRVpro8 vacuum pump is connected to a vacuum pressure gauge to see if it works properly. This pump is used for sample activation prior to experiments, moisture knocking off, and system evacuation once the experiment is over. The vacuum pump line is entirely isolated by a valve from the other parts of the system in order to save the pump from backflow damage caused by the high-pressure gas flow. A stainless steel (SS-316) 1/8 in. tubing (I.D. = 0.069 in., SS-T2-S-028-20) is employed to build the gas connections through the whole setup. All of the valves used in this equipment are of the needle-valve type. The overall uncertainty of this equipment was determined by the consideration of the uncertainties arising from PT, RTD, material purity, and sorbent densities, which was aforementioned as ±0.20%. The commissioning of this setup was done and published in our previously published work.^32^ Here, the gas absorption amount is reported in terms of the mole fraction of the absorbed gas in the DES sample for both gases. The gas amount was calculated initially at the start of the experiment and finally at the equilibrium state following eq 1

1 $$N=\frac{PV}{zRT}$$

where *N*: moles (gas moles in eq 1), *P*: gas pressure in (Pa), *V*: gas volume (m^3^), *z*: gas compressibility factor, *R*: universal gas constant (8.314463 m^3^·Pa·K^–1^·mol^–1^), and *T*: gas temperature (K).

The compressibility factor was estimated using the REFPROP software based on the equation of state published by the National Institute of Standards and Technology (NIST).^33^

The dissolved gas moles in the prepared HDES are the difference between the initial and final amounts of gas at the beginning and the end of the experiment (eq 2).

2 $$N_{gas,dissolved}=N_{gas,i}-N_{gas,f}$$

Gas mole fraction was obtained by eq 3.

3 $$x_{gas}=\frac{N_{gas,dissolved}}{N_{gas,dissolved}+N_{DES}}$$

Here, *N*_DES_ is the DES number of moles and is calculated by the equation

4 $$N_{DES}=\frac{m_{DES}}{M_{w,DES}}=\frac{\rho_{DES}\times V_{DES}}{\sum x_{i}M_{wi}}$$

where *m*_DES_: DES mass (g), *M*_wDES_: DES molecular mass (g/mol), ρ_DES_: DES mass density (g/cm^3^), *V*_DES_: DES sample volume (mL), *x*_*i*_: DES component mole fraction, and *M*_w*i*_: DES component molecular mass (g/mol).

Figure 2 shows an illustration of the high-pressure isochoric gas sorption system, and Figure S1 in the Supporting Information shows the actual setup in the lab. The experimental sorption data for CO_2_ and N_2_ gases were acquired at 25 °C temperature and at a pressure range of 2.5 to 30 bar.

**Figure 2:**
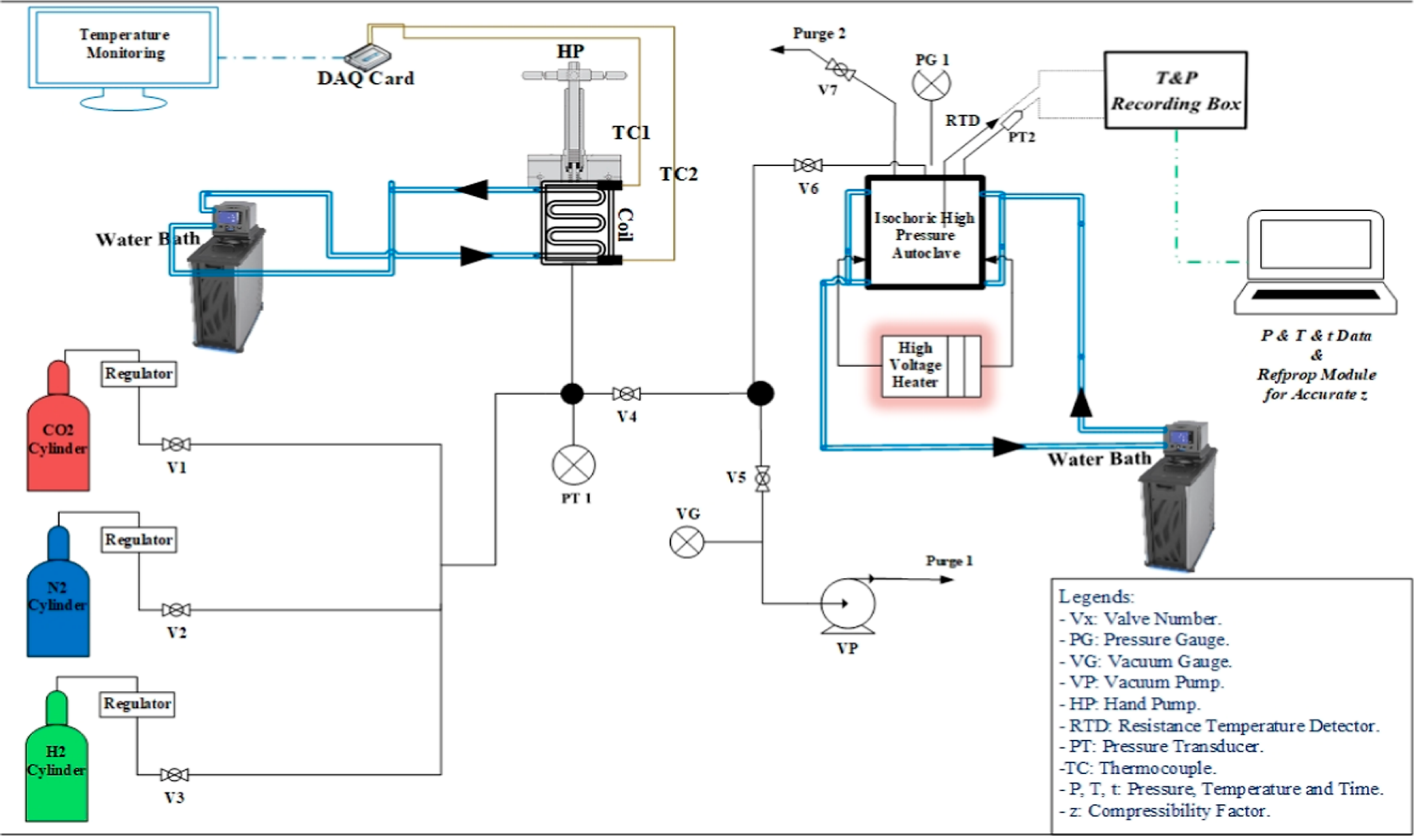
Schematic representation of the high-pressure isochoric gas sorption system.^32,34^ Reprinted (adapted or reprinted in part) with permission from [*J. Environ. Chem. Eng.* **2022,** *10*(5), 108237 and *J. Mol. Liq.* **2023,** *390,* 123114]. Copyright [2022, 2023] [Elsevier].

### 2.4 Material Characterization

Thermophysical property measurements of the prepared natural HDES systems were acquired and presented, particularly, density, viscosity, contact angle, surface tension, and conductivity. Density and viscosity measurement data were collected over the 20–60 °C temperature range. The equipment used for the density measurements was an Anton Paar Density Meter DMA 1001, which comes with a 0.0001 g accuracy magnitude. In the case of viscosity measurements, Anton Paar Rotational Viscometer ViscoQC 300 (accuracy = ±1% full-scale range) provided with Peltier temperature control PTD80 was utilized for the data collection. Ionic conductivity data were acquired using Mettler Toledo SevenCompact Duo S213 equipment (±0.5% accuracy) at the atmospheric pressure and over the ∼24 to ∼32 °C temperature range. The acquisition of the contact angle and the surface tension measurements was carried out using First Ten Angstroms Dynamic Contact Angle Analyzer FTA200 equipment. The contact angle was measured between the prepared natural HDES and four different surfaces which were glass, copper, stainless steel 304, and stainless steel 316 at the atmospheric pressure and the lab temperature.

Thermal gravimetric analysis (TGA) and Fourier transform infrared spectroscopy (FTIR) were measured for the three natural HDES systems. TGA analyses were performed via TA Instruments Q50 TGA equipment with a 5 °C/min rate of heating without a N_2_ gas purge at atmospheric pressure. FTIR spectra were acquired via a PerkinElmer Spectrum 100 FT-IR Spectrometer under the lab conditions of pressure and temperature.

In addition, the purities of camphor, citral, linoleic acid, and piperitone that were used in DES preparation were confirmed using electrospray ionization mass spectrometry (ESI-MS) by flow injection analysis (FIA). These compounds were detected with a FIA solvent of 100% methanol with 1 mM ammonium formate as cations in positive ion mode. ESI-MS was performed on a Thermo LCQ Advantage Max mass spectrometer fitted with a Surveyor solvent delivery and auto sampler instrument (Thermo Electron Corp, West Palm Beach, FL). Solvents were diluted in LC-grade water and injected into the FIA analysis solvent with a flow rate of 50 μl/min into the ion source of the mass spectrometer. Mass spectra showing relative intensities are shown in the corresponding figures, demonstrating the purities of camphor, citral, linoleic acid, and piperitone that were used in experiments. The resolution of the mass spectrometer used is 0.1 Da as per manufacturer specifications.

## 3 Results and Discussion

### 3.1 Thermophysical Properties

Thermophysical properties were investigated to give a deep understanding of the characteristics of the prepared natural HDES systems. The exact and reliable thermophysical property measurements of the DES systems are needed because of the growing interest in utilizing DES materials for gas separation applications. This improves the optimization and the design of the separation process of gas.^35,36^

#### 3.1.1 Density

DES density is a fundamental physical volumetric characteristic. The high accuracy measurement of this property is vital for the detailed design and the feasibility studies for the actual industrial-scale applications of DES.^37^ Typically, density measurements of the DES systems are needed to develop the proper equation of state, which enables the establishment of the industrial-scale implementations of the DES materials.^38^ Likewise, the selection of the proper DES system that has the suitable density magnitude is carried out via lab-scale testing. DES system density varies based on the nature and the molar mixing ratios of the parent constituents that form the DES system.^39^ During this work, the density profiles between temperatures of 20 and 60 °C of the natural HDES systems were tested at atmospheric pressure. Figure 3 demonstrates the densities of the three HDES systems alongside with the isobaric thermal expansion coefficient, which can be explained at the constant pressure as the negative partial derivative of the density natural logarithm with respect to temperature.^22^ As expected, the density was inversely affected by the temperature variation; as the temperature increased, the density values decreased and vice versa. The system of CAM–LnA exhibited the largest magnitude of density on all of the temperature values, whereas the CIT–LnA HDES system showed the least values for density and PIP–LnA was the intermediate case among the three systems. Density differences are referred to the differences in the HBA densities, which, according to the providers, are 0.992, 0.888, and 0.940 g/cm^3^ for CAM, CIT, and PIP, individually, at the room temperature. The exhibited trend of the density–temperature evolution in the three cases was linear, which allows the isobaric thermal expansivity coefficient to be estimated as a temperature function. In this work for the three HDES systems, the computed values of the isobaric thermal expansion coefficient showed a larger magnitude than that of the common type-III DES which is typically lower than 8 × 10^–4^ K^–1^.^23,40^

**Figure 3:**
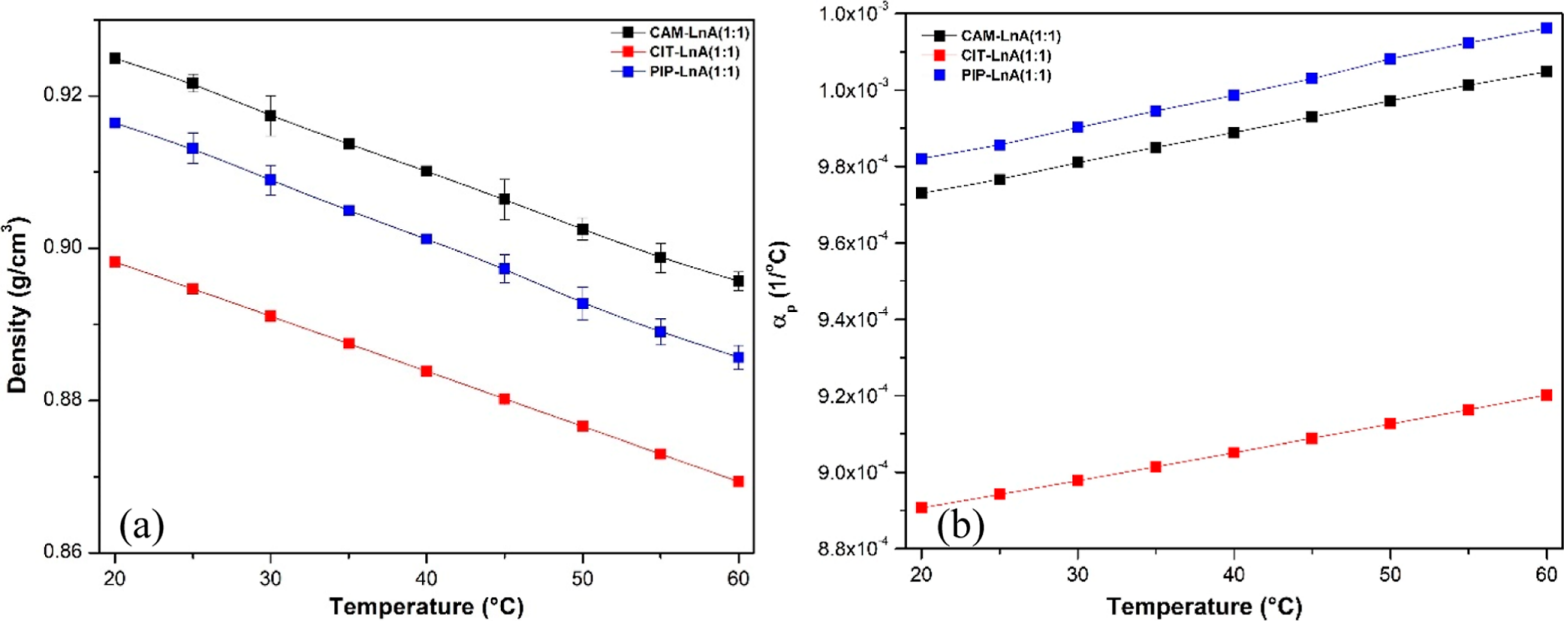
(a) HDES density evolution profile with temperature change. (b) HDES thermal expansion coefficient profile as temperature functions.

#### 3.1.2 Viscosity

Viscosity is also another important thermophysical property for the industrial implementation of DES in general, which is defined as the resistance of the fluid to the deformation at a specific shear rate.^22^ DES system viscosity measurement as a temperature function is essential for potential DES applications such as the high-pressure applications or the lubrication applications.^41^ Figure 4 shows the reported viscosity measurement data for the natural HDES systems in this work. The CAM–LnA natural HDES system was the most viscose system among the three HDES systems. That was anticipated because of the physical state of CAM which is in the solid phase up to ∼172–176 °C as it was reported by the provider as a normal melting point for this material. In comparison, the other HBAs which are CIT and PIP are in the liquid phase at the room-temperature condition. With the temperature increase, the gap between the HDES system viscosities becomes smaller. The viscosity values at 20 °C were 14.62, 7.46, and 9.88 mPa s for CAM–LnA, CIT–LnA, and PIP–LnA, respectively. At 60 °C temperature, the viscosity magnitudes of these systems reduced to be 4.6, 2.91, and 3.41 mPa s, individually. All the viscosity magnitudes in the research were less than 500 mPa s, which puts them under the category of low-viscosity natural DES.^39^ Therefore, these natural HDESs should be easy to handle and pump and appropriate for various applications such as the CO_2_ capture and gas separation, which was considered in this work. Moreover, the low-viscosity DES system will not hinder the operations of heat and mass transport, which does not need to oversize the equipment. In comparison with the common 1-butyl-3-methylimidazolium tetrafluoroborate ionic liquid viscosity at 25 °C, the viscosity of this IL is around ∼180 mPa s^42^ compared to ∼6.5, ∼8.5, and ∼12.5 mPa s for the CIT–LnA, PIP–LnA, and CAM–LnA, respectively. Also, these viscosity values of the studied DES systems are much lower than the viscosity of ChCl–urea (1:2), ChCl–glycerol (1:2), and ChCl–levulinic acid (1:2), at the same temperature and they have a magnitude of 750, 259, and 227 mPa s, respectively.^22^ Moreover, diethanolamine has a viscosity of ∼640 mPa s at 25 °C, which is way higher than that of the prepared DES systems.^43^

**Figure 4:**
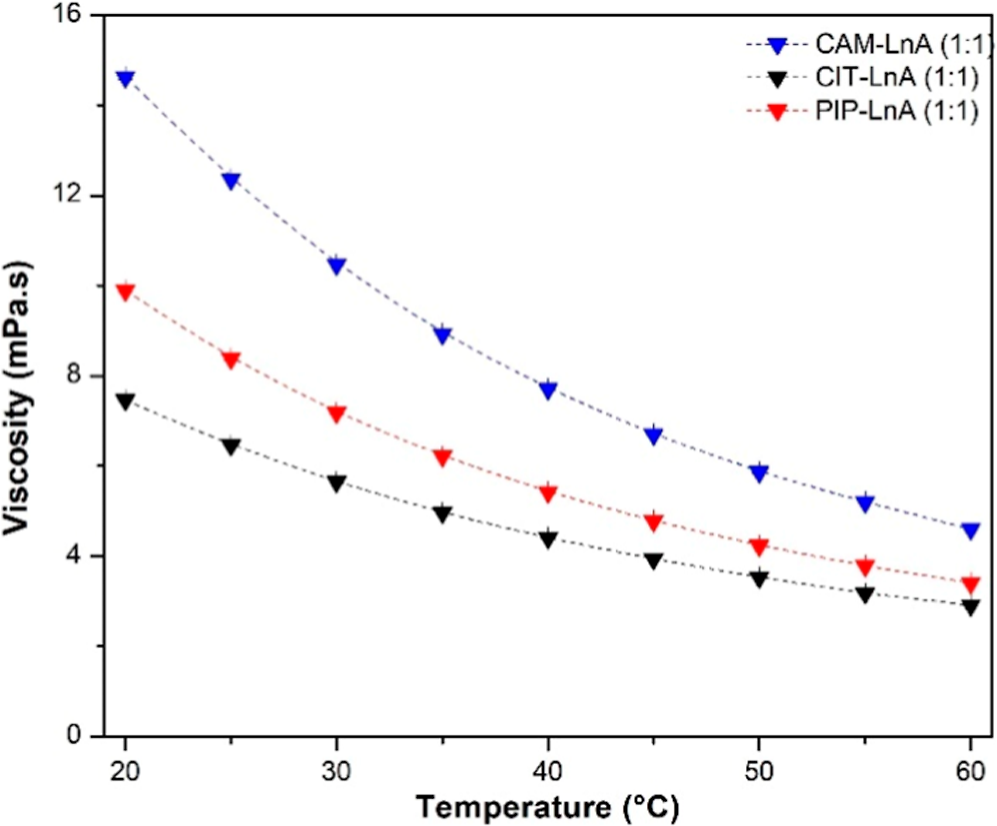
Experimental viscosity data of the natural HDES systems are temperature dependent.

Because the viscosity changes with temperature for the three HDES systems follow the non-Arrhenius behavior, the viscosity values were fitted using the Vogel–Fulcher–Tammann (VFT) model. The VFT model equation is shown below^23,44^

5 $$\eta =\eta_{0}\exp\left(\frac{D_{f}}{T-T_{0}}\right)$$

where η_0_, *D*_f_, and *T*_0_ are the VFT model fitting parameters. η and η_0_ are the viscosities at any temperature (*T*) and at the ideal glass transition (*T*_0_), respectively. The parameter *D*_f_ = *B*/*T*_0_ is Angell’s strength (fragility measurement) parameter and *B* is a constant. Table 1 demonstrates the VFT model parameters in addition to the values of the adjusted *R*-square.

**Table 1:** VFT Model Parameters and Fragility Measurement Parameters of the HDES Systems

natural HDES	η_0_/mPa s	*B*/°C	–*T*_0_/°C	*D*_f_ = *B*/*T*_0_	adj. *R* ^2^
CAM–LnA (1:1)	0.0304	1068.31	152.94	6.99	0.99993
CIT–LnA (1:1)	0.08181	681.77	131.06	5.20	0.99997
PIP–LnA (1:1)	0.08542	657.86	118.47	5.55	1.00000

The viscosity VFT curves and their residual graphs are reported in Figures S2 and S3 in the Supporting Information. The small regular residual values and their scatter distribution demonstrated that the viscosity data were properly fitted to the VFT model. The values of the *T*_0_ parameter are connected to the glass transition temperatures.^44^

At the aforementioned temperature (*T*_0_), the molecules of the material are thought as totally frozen.^45^ Getting to a temperature higher than that, the material would be liquified. Although the glass transition temperature is not as distinct as the melting point, it can be utilized as an indirect method of confirming the low melting point of the formed DES. The melting points of CAM, CIT, PIP, and LnA are +175, −10, −29, and −5 °C, respectively, and the predicted melting points by the COSMO-RS of CAM–LnA, CIT–LnA, and PIP–LnA were −23, −53, and −58 °C, accordingly. Here, the calculated *T*_0_ values were −152.9, −131.1, and −118.5 for the three systems, respectively. Although they are different from the predictions of COSMO-RS, these values are lower than those of the single components of the DES and satisfy the very low melting point conditions. In a sense, these values confirm that the prepared solvents are DESs.

Although Karl Fischer titration was not performed, the DES samples were prepared using predried components and stored under dry, sealed conditions. Viscosity measurements were conducted immediately after preparation to minimize the environmental moisture interference. COSMO-RS and DFT simulations, including a small number of water molecules, showed no significant disruption of the hydrogen-bonding network between HBD and HBA components, suggesting structural robustness of the DESs to trace moisture. The consistency of the experimental viscosity measurements further supports this observation.

#### 3.1.3 Contact Angle and Surface Tension

The interfacial characteristics of the solvents over the different surfaces are explained through fundamental physical properties, which are contact angle and surface tension. The measurement of these physical properties is vital for the design of separation application. The measured contact angle and surface tension values for the HDES systems are reported in Table 2 below. Typically, contact angle data report the HDES systems’ wettability properties. Overall, the low values of the contact angle can be noticed, meaning that the studied systems have a high wettability potential. The highest value was reported as 42.7° for PIP–LnA on the copper surface, and the lowest was 24.9° for CIT–LnA over the glass surface. The reported experimental values of the surface tension for all the explored HDES systems are around ∼30–32 dyn/cm, which is within the same range of the reported monoterpenoid–LnA HDES surface tension in our published work.^46^ These values are less than the reported surface tension magnitude of the popular ChCl-based system which is ∼47.5 dyn/cm.^47^

**Table 2:** Contact Angle of the HDES and Their Surface Tension

	contact angle (deg)				
HDES systems	copper	glass	SS-304	SS-316	surface tension dyn/cm
CAM–LnA (1:1)	26.5 ± 2.7	31.9 ± 2.1	25.2 ± 2.2	32.6 ± 2.9	30.0 ± 1.4
CIT–LnA (1:1)	29.9 ± 2.4	24.9 ± 2.5	27.3 ± 4.6	38.5 ± 3.2	30.8 ± 1.4
PIP–LnA (1:1)	42.7 ± 4.8	30.8 ± 3.2	31.7 ± 3.9	32.4 ± 2.3	32.3 ± 1.5

#### 3.1.4 Ionic Conductivity

Conductivity is one of the necessary characteristics for industrial electrochemical applications. Investigating this physical property is vital for the DES to gain substantial industrial-scale application. In this research, ionic conductivity magnitudes were reported in microsiemens per centimeter (mS/cm) as a function of temperature over the range of ∼24 °C to ∼32 °C, as shown in Figure 5. Here, conductivity data is provided as part of a comprehensive characterization of the DES systems, offering valuable insights for potential applications beyond gas solubility. Additionally, reporting such data ensures that the very low conductivity values are consistent with the characteristics of type-V DES, which are inherently nonionic in nature. This serves as confirmation of the expected thermophysical behavior of the investigated systems. Naturally, the DES conductivity magnitude increases as the temperature increases, which is a clear behavior in the explored HDES systems. The ionic conductivity magnitude is impacted by the strength of HBA–HBD interactions.^39^ Here, HBA and HBD species are nonionic constituents, which can justify the very small magnitude of the conductivity. The reported conductivity magnitudes of the investigated natural HDES systems over the whole range of temperature are less than 1 mS/cm, which is defined as a very low conductivity value.^48^

**Figure 5:**
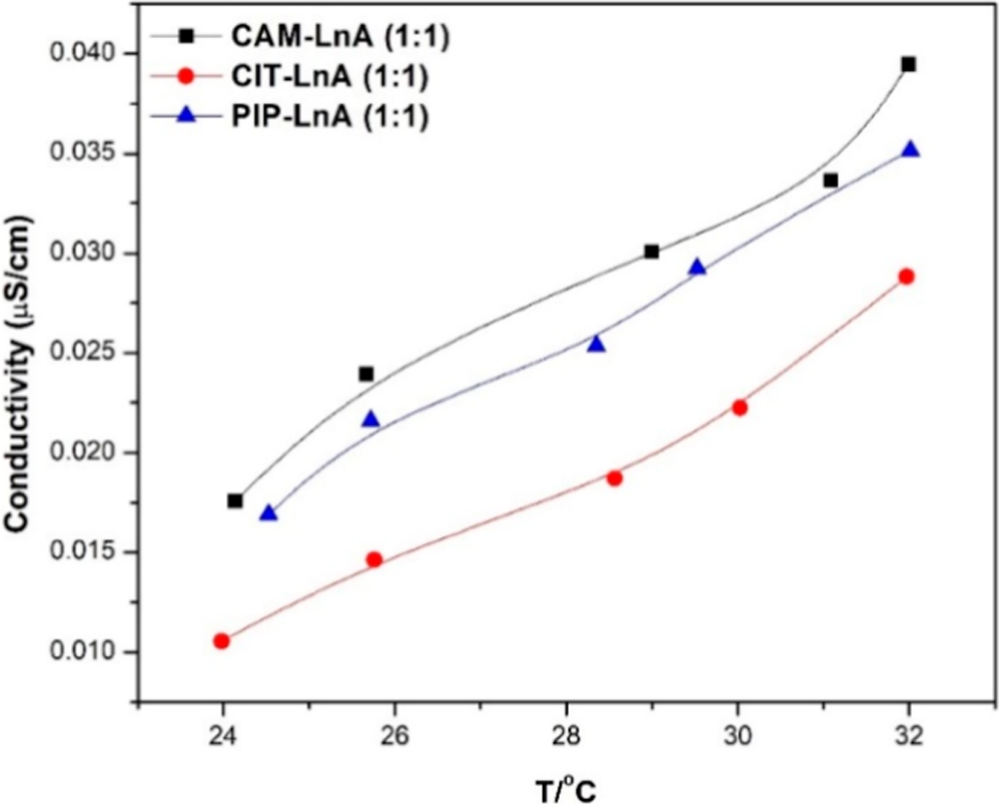
Ionic conductivity for the three HDESs.

#### 3.1.5 Thermal Gravimetric Analysis (TGA)

Figure 6 reports the experimental TGA data of the explored HDES. This analysis was executed to inspect the prepared hydrophobic NADES thermal stability and their limitations. The test was carried out over the range of temperature from the lab temperature up to 250 °C. Using TGA data, the thermal degradation onset temperature (*T*_onset_) parameter of the test sample is defined. This parameter explains the applicable temperature range of the explored sample; hence, the tested specimen can be employed in operations within a temperature range away from the material disintegration limits. This parameter is usually defined based on the TGA data as the intersecting point of the baseline and the line of the first inflection point.^49^ Here, the estimated *T*_onset_ parameter values for the investigated HDES are ∼70 °C for the CAM–LnA case and ∼88 °C for the other two DES systems (CIT and PIP systems). The mass drop percentages from the start of the test until reaching the *T*_onset_ value were ∼5.0% for the CAM–LnA system and ∼3.0% in the cases of CIT–LnA and PIP–LnA systems. The systems of CIT–LnA and PIP–LnA exhibited an overall similarity in their mass change curves. They showed higher stability during the heating process until reaching ∼150 °C in comparison with CAM–LnA stability. Nevertheless, the three HDES systems lost ∼30.0% of the first mass amounts of the specimens. A plateau was reached by the three systems over the temperature domain of ∼140 °C to ∼170 °C. At a higher temperature than ∼170 °C, all systems got in a sharp degradation stage until the end of the experiment. At ∼160 °C, the mass change lines of CAM–LnA and PIP–LnA systems became closer from each other and the change curve of the CIT–LnA system was under the other lines until reaching the temperature value of ∼220 °C.

**Figure 6:**
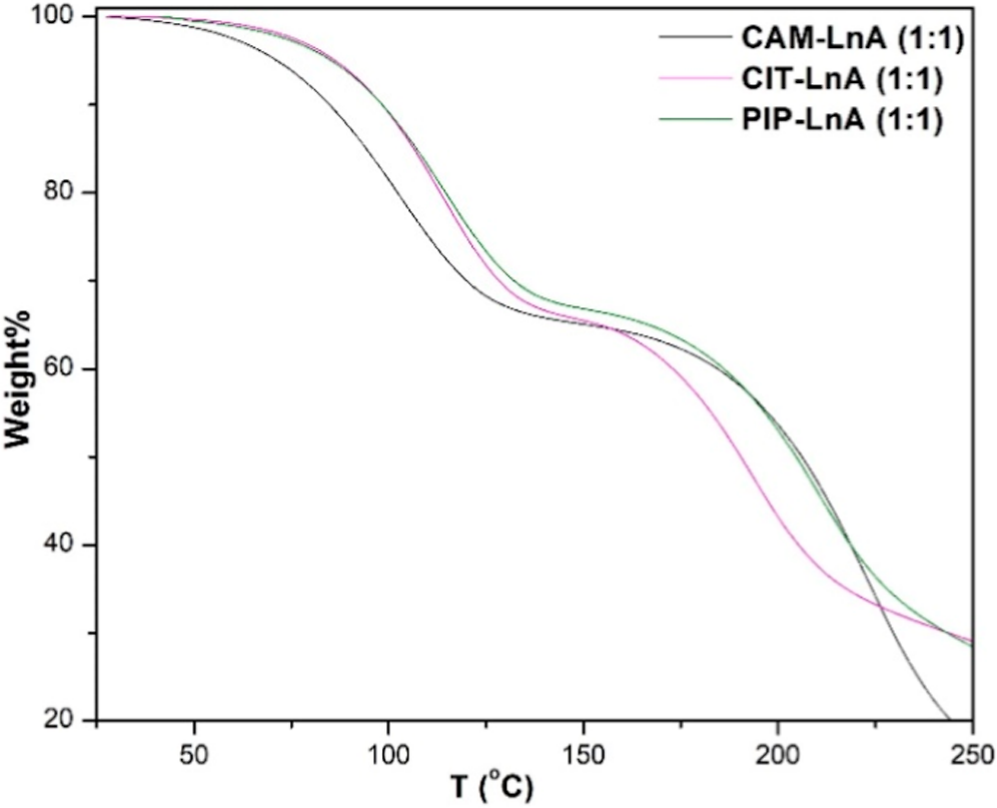
TGA data curves of the three LnA-based HDES systems.

The TGA experiments were performed at a heating rate of 5 °C/min under a nitrogen flow. The sloped baseline and early mass losses observed below 150 °C may reflect gradual evaporation of the more volatile DES components or trace impurities. The presence of one or more plateaus likely corresponds to the staged volatilization of individual DES constituents based on their boiling point and composition ratios. While dynamic TGA is useful for comparative thermal screening, we acknowledge that isothermal TGA runs and repeated thermal cycling would better simulate real-world desorption behavior and capture long-term stability.

#### 3.1.6 Fourier Transform Infrared Spectroscopy (FTIR)

Fourier transform infrared spectra were investigated to define the probable existing functional groups and the chemical bonds that are connected to predefined frequencies. Figure 7 reports the FTIR spectra acquired in this work. The collected FTIR curves fall in the mid-IR spectrum range (4000–600 cm^–1^). This IR range is split into four domains which are (1) single-bond (–) domain (4000–2500 cm^–1^), (2) triple-bond (≡) domain (2500–2000 cm^–1^), (3) double-bond (=) domain (2000–1500 cm^–1^), and (4) domain of fingerprint (1500–600 cm^–1^). All of the collected FTIR curves have more than five peaks, which put the studied HDES in the category of complex molecules. In the region of a single bond (–), the peaks close to the 3000 cm^–1^ wavenumber revealed the presence of the aromatic structure in the HDES. Around 3000–2800 cm^–1^ wavenumber, the peaks confirm the aliphatic bonds (C–C). Within the range of the triple bond (≡), there were no peaks detected, which means no triple bonds in these HDES combinations. The bands around ∼1700 cm^–1^ in the double-bond range demonstrated the existence of the vinyl (C=C) and the carbonyl (C=O) groups.^50^ Generally, by looking at the spectrum of CAM, CIT, PIP, and LnA, individually, compared to the spectrum of the formed systems out of them, it can be noticed that the peaks of the formed systems came from the original two components but with different transmittance values. In other words, the resulting spectra for the DES systems are pretty much a merge between the spectrum of the HBA and the HBD. The transmittance change in FTIR spectra reveals that the amount passed IR through the tested sample at designated wavelengths has changed. This means that the tested sample absorbs less or more IR radiation at that wavelength. This can be credited to the chemical bonds’ concentration change in the tested sample. It is known DES system formation has no presence of chemical reactions^51^ and here the results of FTIR are in agreement with this information. For instance, the CAM–LnA system has two peaks around the wavenumber of 1750 cm^–1^. These two peaks came from CAM and LnA with a change in the transmittance value. Similarly, the peaks below 3000 cm^–1^ came from the combination of the peaks of CAM and LnA in that range of wavelengths. The same principle applies to the fingerprint region. In the other DES cases also, the same principle applies. The FTIR spectra of the three DES systems have similarity due to the similarity of the structures of the HBA components and the common HBD used to form the systems.

**Figure 7:**
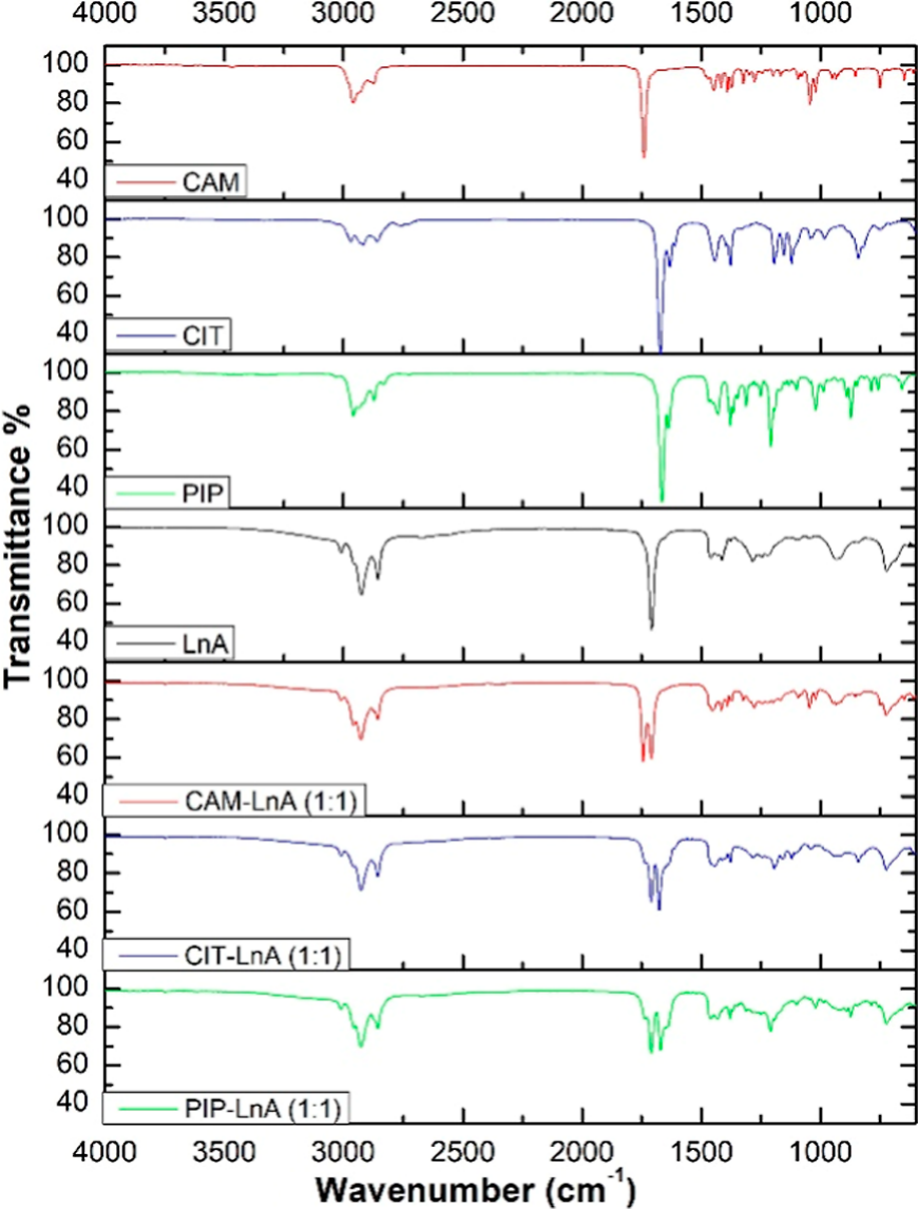
FTIR data curves of the prepared hydrophobic NADES.

Upon comparison of the FTIR spectra of the individual components (CAM, CIT, PIP, and LnA) to those of the formed HDES systems, it is evident that the peaks observed in the DES systems largely originate from their parent components. This suggests that no new bonds or chemical reactions have formed during the DES creation, which aligns with previous findings regarding the physical mixing nature of DES formation [48]. In particular, changes in transmittance values across the spectra indicate concentration-dependent variations in bond interactions but not the formation of new functional groups. For example, the CAM–LnA system shows two prominent peaks around 1750 cm^–1^, which correspond to characteristic stretching vibrations from both CAM and LnA, albeit with shifts in intensity due to the change in concentration. Similarly, peaks below 3000 cm^–1^ arise from the combined contributions of both CAM and LnA, with shifts in the intensity and position. This pattern repeats across the fingerprint region for all tested DESs, indicating that the interaction mechanisms within these systems are based on van der Waals forces and hydrogen bonding, typical for nonionic DES. Furthermore, the FTIR spectra of the different DES systems reveal a high degree of similarity, especially in regions corresponding to the HBD component (LnA), due to its structural consistency across all systems. This supports the idea that the main differences between the spectra of the HDES systems are driven by minor shifts in intensity due to different concentrations, rather than any new chemical interaction. These observations are consistent with the known behavior of DES systems, which do not form new chemical bonds but rely on physical interactions such as hydrogen bonding. The lack of significant spectral shifts supports this assertion. The transmittance changes merely reflect the varying absorptive capacities of the systems at different wavelengths, depending on the relative concentration and interaction of the original components.

#### 3.1.7 Electrospray Ionization Mass Spectrometry (ESI-MS)

The spectral profiles for these samples are provided in Figures S5–S8. The mass spectrum of linoleic acid shows (Figure S6) a dominant peak at 279.53 *m*/*z*, corresponding to its protonated molecular ion, with smaller fragment peaks resulting from the expected cleavages in the long hydrocarbon chain. As a nonconjugated fatty acid, its fragmentation pattern is primarily dictated by its carboxylic acid group and chain scission, leading to predictable and stable fragments without forming unique reactive byproducts. The stability of linoleic acid during ionization was also confirmed by Müller et al., and it can be attributed to its unsaturated structure, which is less prone to forming unstable radicals under standard ionization conditions, as evidenced by the dominant peak at ∼279 *m*/*z* and minimal secondary peaks during analysis.^52^ The mass spectrum of piperitone shows (Figure S7) a dominant peak at 153.27 *m*/*z*, corresponding to its protonated molecular ion with very few significant secondary fragments, indicating its stability during ionization. As a nonconjugated ketone, piperitone lacks aldehyde functionality and conjugated double bonds found in citral, resulting in fewer reactive fragmentation pathways and minimal impurity formation. The mass spectrum of camphor exhibits (Figure S8) a dominant peak at 153.27 *m*/*z*, representing its protonated molecular ion with negligible fragmentation, reflecting the stability of its rigid bicyclic structure. Camphor’s saturated ketone group and absence of conjugation make it highly stable under ionization, which minimizes byproduct formation or reactivity during processing. The mass spectrum of citral shows (Figure S9) a dominant peak at 153.07 *m*/*z*, corresponding to its protonated molecular ion ([M + H]^+^), along with a significant peak at 135.27 *m*/*z*. This peak is likely specific to the production or extraction process of citral due to its nature as a conjugated aldehyde, which makes it prone to forming unique byproducts or impurities such as oxidized terpenoid derivatives. This result aligns with the expected fragmentation pathways of terpenoid derivatives specific to citral, as supported by its standard profile listed in the NIST Chemistry WebBook.^53^ In contrast, simpler terpenes, such as camphor and piperitone, lack conjugation, making them less chemically reactive and significantly less susceptible to forming similar byproducts.

### 3.2 CO_2_ and N_2_ Experimental Solubility Data and COSMO-RS Predictions of Solubility

#### 3.2.1 Experimental and COSMO-RS Absorption Data of CO_2_

The experimental absorption data were acquired at 25 °C temperature and over the gas pressure range of ∼2.5 bar to ∼31 bar. In this work, gas sorption was estimated and reported as mole fractions of the gas in the DES sample. Experimental gas sorption data and COSMO-RS predictions are shown in Figure 8a.

**Figure 8:**
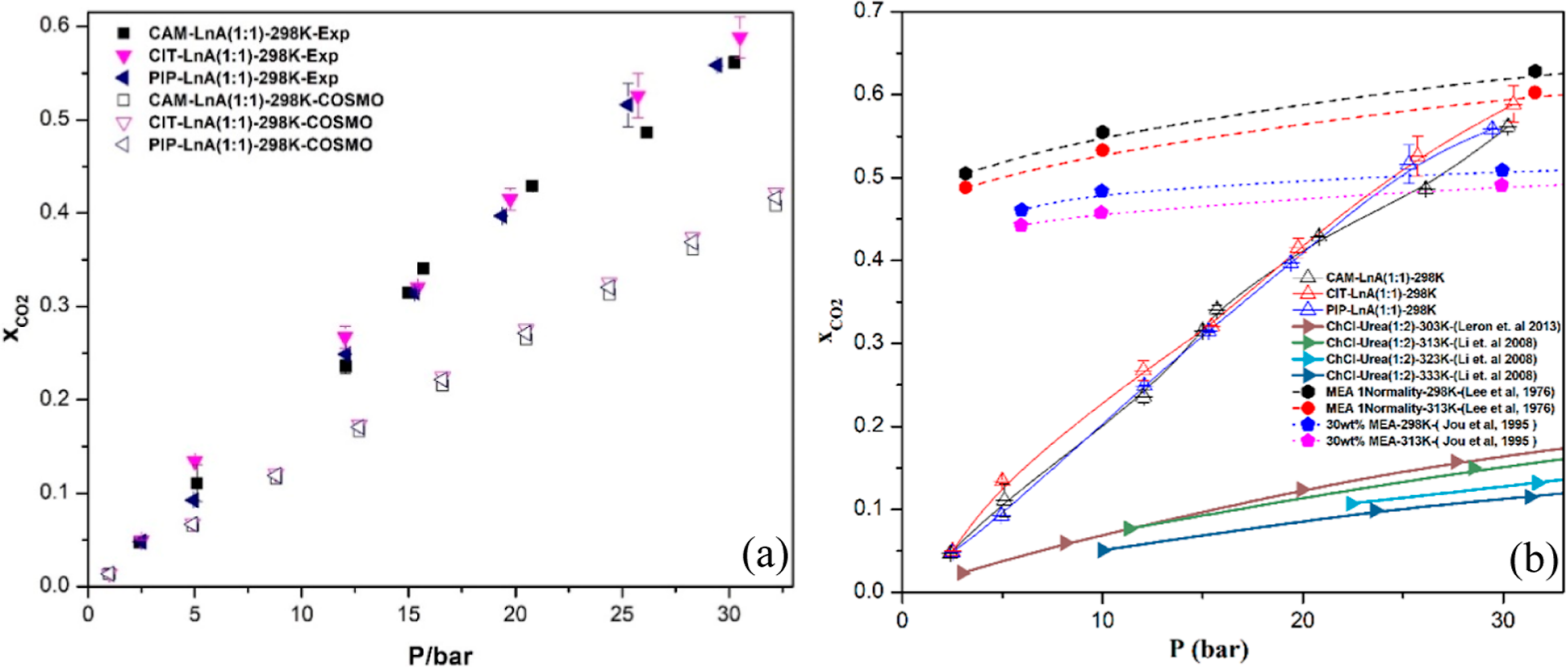
CO_2_ experimental absorption compared to (a) COSMO-RS predictions and (b) the conventional amine and DES system.

As usual, the absorption magnitude of the CO_2_ gas by the tested natural HDES was positively affected by the gas pressure increase. Clearly, the lowest CO_2_ capture value was ∼0.05 at 2.5 bar of gas pressure for the three prepared systems, while the highest achieved sorption magnitude was ∼0.58 at ∼31 bar by the CIT–LnA system. At 25 bar, a mole fraction absorption of ∼0.47 for all systems matched the performance of 30 wt % aqueous monoethanolamine (MEA) at 25 °C. Similarly, at around ∼30 bar pressure, the CO_2_ absorption of the three systems reached very close to the absorption at the same pressure by one normal MEA solution. In comparison with the most common DES system which is ChCl–Urea (1:2) known as reline,^54^ the CO_2_ capacity of the three DES system is way higher than the reline. For example, at a pressure of ∼30 bar, the absorption by the prepared systems in this work is around 5 times the absorption by reline. Figure 8b shows a comparison between the absorption capacity of the prepared DES systems in this study and the traditional amine solution and reline DES. Absorption values by all systems are close to each other; nevertheless, the studied systems did not show the tendency to reach the saturation state within the tested pressure range. In addition to the experimental sorption data, theoretical absorption values were predicted via COSMO-RS software. COSMO-RS predictions were collected because they provide fast selections and quantitative analyses, which cut down the number of experiments in the design of experiments.

A systematic gap between the predictions and the experimental data starts over ∼2.5 bar and continues to grow with the pressure increase. The HBD purity effect on the absorption of CO_2_ gas was investigated. Figure S4 shows the absorption comparison between 85.0% purity HBD-based DES and 95% purity-HBD based DES. The comparison revealed that there is no significant impact of the HBD purity on the CO_2_ absorption. Based on that comparison, all the investigations were carried out using 85.0% purity LnA as HBD rather than using 95.0% purity LnA. Over the course of this work, the procedure of the gas absorption included an implicit reusability (cyclability) test. This was done by exploiting the same DES sample for the whole pressure range measurements without changing the sample inside the equilibrium cell. After the solubility was scanned over the whole pressure range (7 pressure points), the experiment at each pressure point was repeated at least two times. Following this protocol, the reusability was examined completely, and there was no observation of the decline in the gas absorption capacity nor the amount of the liquid sample. Similarly, this experimental procedure revealed that the sorption mechanism here is physisorption absorption and does not involve any reaction. That is because the generation stage of the DES sample was carried on only by depleting the gas and applying a vacuum for 2–5 min without any external heating step.

#### 3.2.2 Absorption Kinetics of CO_2_

The speed of the CO_2_ capture was assessed based on the solubility kinetics method. Figure 9 is the representation of the CO_2_ absorption kinetics of the prepared HDES at a 25 °C isotherm and 10, 20, and 30 bar pressures. The reported data were estimated through computing the cumulative amount of CO_2_ absorbed as a percentage of the final sorption magnitude at the end of the experiment. The absorption rate is positively affected by the pressure increase, where the dissolution of CO_2_ in the DES is faster at higher pressure points.

**Figure 9:**
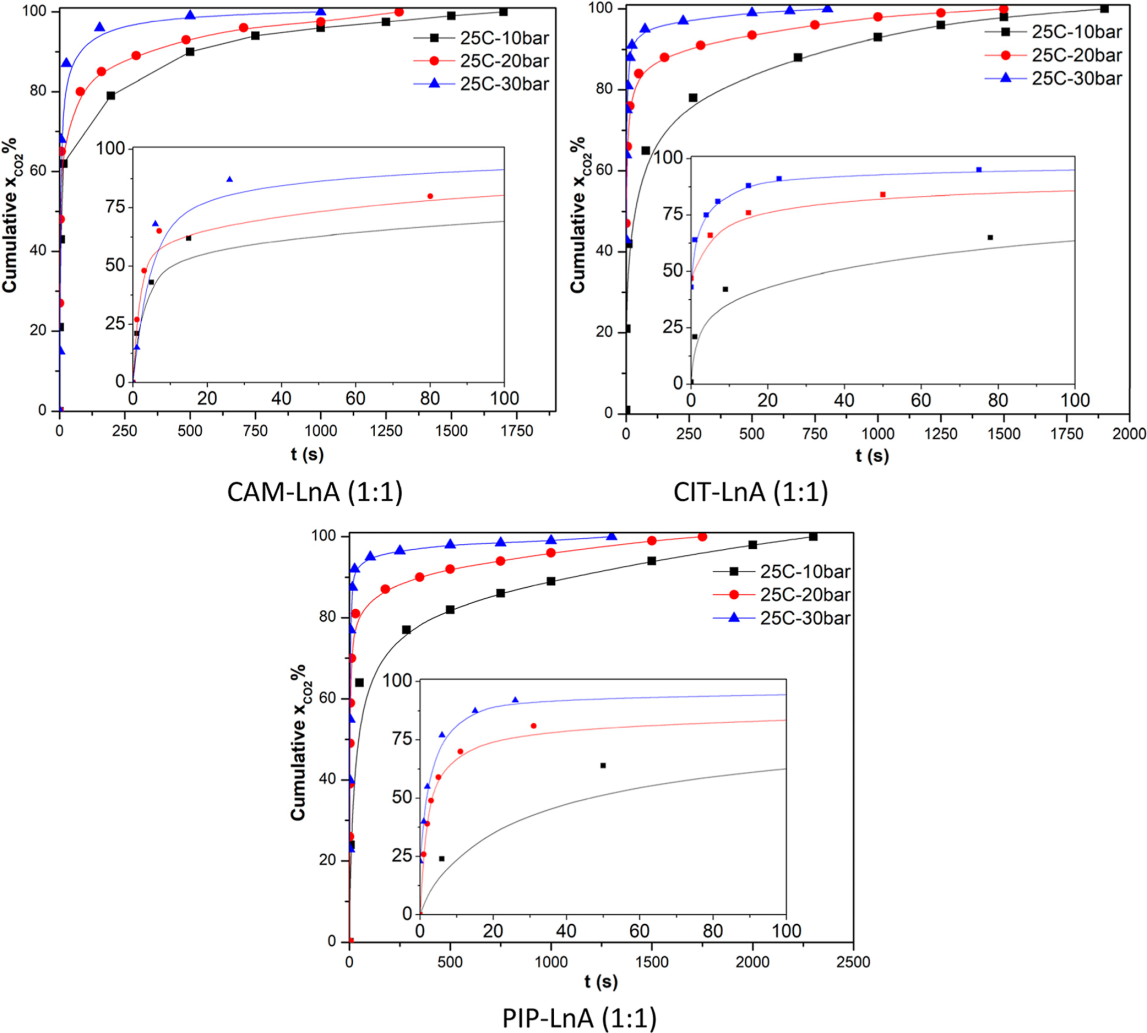
Kinetics of CO_2_ solubility in the studied HDES systems.

According to the data presented in the graph, the rapidest solubility of CO_2_ gas was at the pressure of 30 bar, and the slower process was at 10 bar. During the initial 100 s, the cumulative sorption values were ∼62%, ∼77%, and ∼90% at the pressures of 10, 20, and 30 bar, correspondingly, for CAM–LnA DES, whereas the sorption magnitudes by CIT–LnA during the initial 100 s of the experiment were ∼62%, ∼87%, and ∼92%, respectively, at 10, 20, and 30 bar pressures. In the case of PIP–LnA, cumulative sorption values were ∼62%, ∼81%, and ∼90%, respectively, at 10, 20, and 30 bar of pressure. The times needed to reach saturation at 10, 20, and 30 bar, respectively, were 28.3, 21, and ∼17 min for CAM–LnA, 31, 24, and 14 min for CIT–LnA, and 38, 29, and 22 min for the PIP–LnA system. Hua et al. reported an increase in the used liquid sample viscosity magnitude from 0.15 Pa s to 1.3 Pa s after the CO_2_ absorption.^55^ Also, Gu et al. measured the viscosity for 4 DES systems before and after the CO_2_ absorption process. They found a strong impact of the CO_2_ sorption on the viscosity of the used DES during the process.^56^ The magnitude of the [TETA]­Cl–thymol (1:3) viscosity increased 33 times, ∼57 times in the case of [TEPA]­Cl–thymol (1:3), and 9 times in the case of [TEPA]­Cl–thymol (1:5) and [TETA]­Cl–thymol (1:5). In the current work, the absorption happened during the initial 5 min. That can be explained by the high impact of CO_2_ sorption on the viscosity of the DES. Hence, at a bigger viscosity magnitude, the process will be slower due to the mass transport hindrance.

#### 3.2.3 Experimental and COSMO-RS Absorption Data of N_2_ and Gas Selectivity

The experiment of N_2_ absorption and the calculations of N_2_ absorption magnitudes were carried out by using the same methodologies that were employed with CO_2_ gas. Figure 10 shows the reported experimental and COSMO-RS sorption data for N_2_ gas.

**Figure 10:**
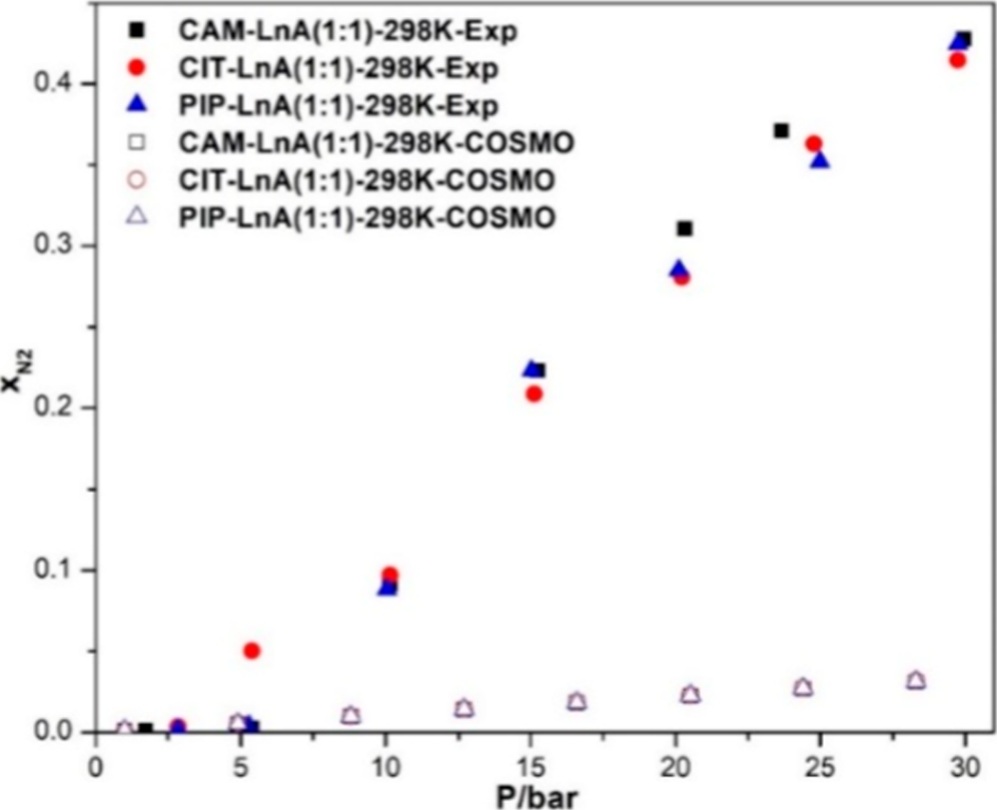
N_2_ experimental absorption data in comparison with the COSMO-RS predictions for the investigated NADES.

The quantification of N_2_ solubility is reported in terms of the gas mole fraction in the HDES specimen. Similar to the trend of the CO_2_ solubility, N_2_ solubility is positively impacted with pressure escalating. The pressure effect on the solubility in the predicted values is like that in the experimental data. The predicted values and the experimental values depart from each other at 5 bar pressure for the CIT–LnA case and at 10 bar for the other cases. Overall, the three systems showed pretty much similar values to each other. At ∼30 bar pressure, the maximum solubility of N_2_ was achieved by the CAM–LnA system and the least value was by CIT–LnA with magnitudes of 0.428 and 0.0415, correspondingly. At the pressure of ∼2.5 bar, the highest absorption was 0.004 and the lowest was 0.001 by CIT–LnA and CAM–LnA, respectively. The sorption trends of N_2_ gas show the ability to absorb more gas at an escalated pressure than those applied in this research. Regarding the poor prediction of N_2_ solubility in DES, it is well known that COSMO-RS is more commonly applied to predict CO_2_ solubility. To our knowledge, COSMO-RS has only been used to predict N_2_ solubility in DES in our previous work^46^ and in the work of Kamgar et al., 2017.^57^ In both cases, COSMO-RS performed poorly in predicting the N_2_ solubility. However, generally, these discrepancies in the CO_2_ and N_2_ gas solubility prediction arise because the COSMO-RS typically predicts solubility well under conditions of high temperature and low pressure, where gases can be assumed to behave ideally. Increasing pressure significantly impacts the accuracy of COSMO-RS predictions for the two gases, as demonstrated by the increasing gap between the experimental and predicted data observed in our work and prior studies. Despite N_2_ being a smaller molecule than CO_2_, the interaction of N_2_ molecules with DES in the liquid phase involves complex, less predictable behaviors, which may not be fully captured by COSMO-RS. The differences in the predictive accuracy for N_2_ and CO_2_ arise from the inherent challenges of modeling weak van der Waals interactions in nonpolar gases like N_2_ using COSMO-RS. In contrast, as aforementioned at high-temperature and low-pressure conditions, the model excels in capturing the stronger electrostatic and hydrogen-bonding interactions present in polarizable molecules like CO_2_, highlighting its strengths in systems dominated by such forces while recognizing limitations in nonpolar scenarios. Hence, this model needs more enhancements to be able to predict the solubility under high-pressure and low-temperature conditions.

Any capture technology requires a proper level of selectivity of CO_2_, because separation of CO_2_ from the gas mixture of the effluent stream is a challenge. Normally, CO_2_ is emitted as a component of a mixture of gases which includes N_2_ gas as a major constituent.^58^ In this research, the ideal selectivity of CO_2_ gas by the employed hydrophobic NADES was quantified following a single gas solubility method. This method is based on the use of the gases’ mole fractions in the liquid phase at constant pressure and temperature as in eq 6.^59^ At the same temperature and pressure magnitudes, N_2_ absorption values were assumed as the baseline and the solubility of CO_2_ were divided by that of N_2_. Table 3 reports the ideal selectivity of CO_2_ against N_2_ (S^I^_CO_2_/N_2__) by the studied natural HDES.

6 $$S_{{CO}_{2}/N_{2}}^{I}={\left(\frac{x_{{CO}_{2}}}{x_{N_{2}}}\right)}_{P,T}$$

**Table 3:** CO_2_ Gas Selectivity against N_2_ Gas by the Prepared HDES

	S^I^_CO_2_/N_2__		
*P*/bar	CAM–LnA (1:1)	CIT–LnA (1:1)	PIP–LnA (1:1)
2.5	41.4	13.9	27.2
5	44.2	2.7	22.4
10	2.3	2.4	2.4
15	1.4	1.5	1.4
20	1.4	1.5	1.4
25	1.2	1.5	1.5
30	1.3	1.5	1.5

The best HDES system in selectivity performance was CAM–LnA at the pressure of 2.5 and 5 bar; however, all systems showed similar selectivity values at a higher pressure than 5 bar. Based on these findings, CAM–LnA DES is the best option for low-pressure-separation condition utilization, particularly in the oxy-fuel method and direct air capture technology. That is because the typical operational conditions of these methods are 1 bar pressure and a temperature of −55 °C for the oxy-fuel technology and 25 °C for the process of direct air capture.^60^

#### 3.2.4 Absorption Kinetics of N_2_

The assessment of N_2_ absorption speed was executed following the same procedure that was performed for the CO_2_ capture process pace evaluation. Figure 11 presents the solubility kinetics of the N_2_ absorption process for the studied HDES at 25 °C and 10, 20, and 30 bar pressure.

**Figure 11:**
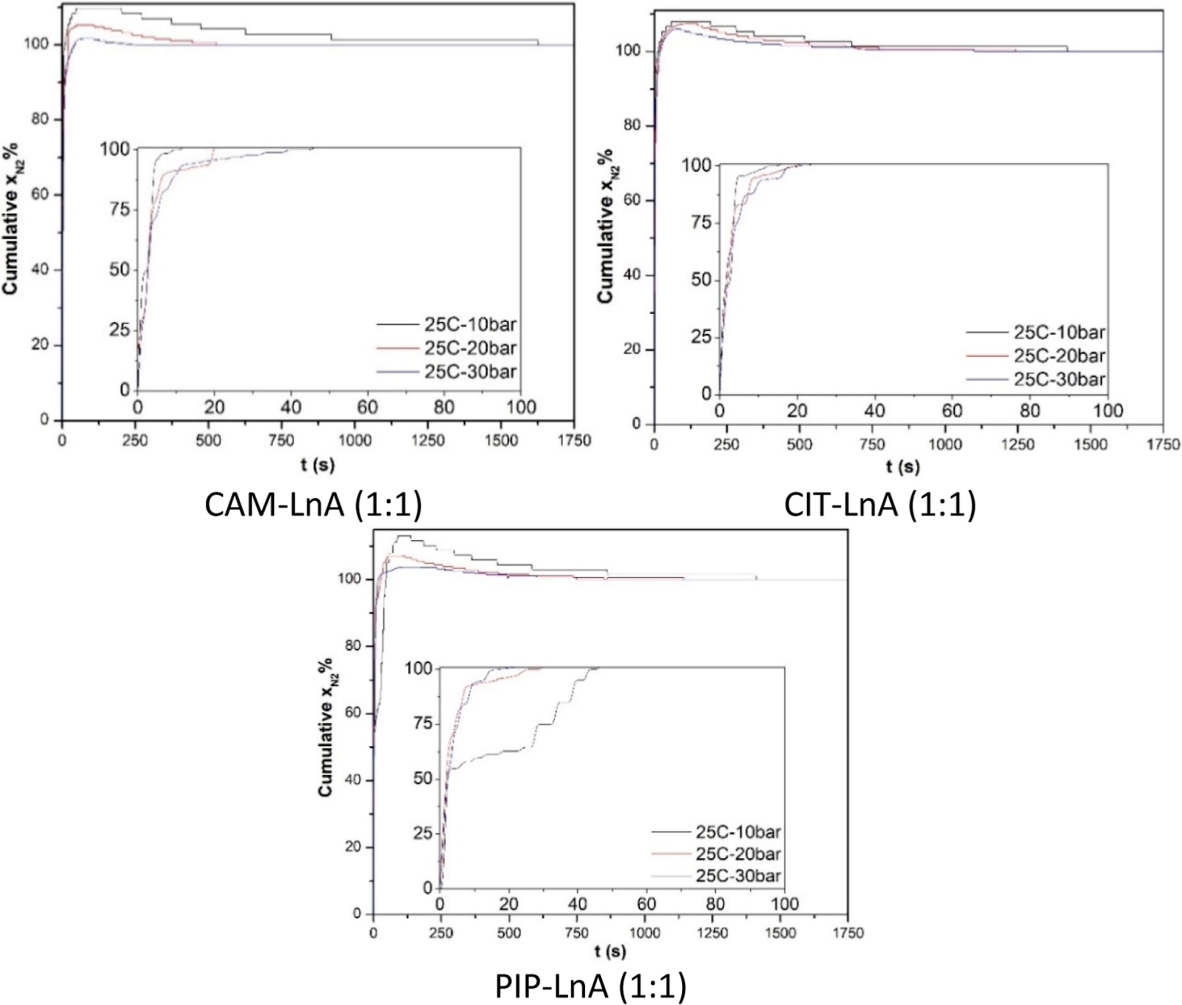
N_2_ absorption process kinetics for the prepared HDES systems.

All the studied DES systems showed a phenomenon of an excess N_2_ absorption stage before the process reached the equilibrium state. Pressure changes affect the excess amount of N_2_ absorption and the equilibrium time. With increasing the gas pressure, the excess magnitude of absorption was reduced, and the time of equilibrium was reduced too. At the gas pressure of 30 bar, the excess absorption was at its lowest level and the equilibrium time was the least too. At the gas pressure of 10 bar, the largest excess absorption was observed, and the equilibrium time was the longest. The equilibrium time was 27, 10, and 4 min at 10, 20, and 30 bar pressures, respectively, in the case of CAM–LnA HDES. While in the case of CIT–LnA DES, the equilibrium time was 25, 21, and 19 min at 10, 20, and 30 bar, separately. Equilibrium time in the PIP–LnA case was 23, 20, and 13 min at the same previous pressures. The overshooting phenomenon in the solubility kinetics illustrated an initial spike state in the sorption that was higher than the equilibrium level, later followed by the equilibrium. At the beginning of the sorption experiment, the concentration difference between the liquid HDES phase and the gas phase was at its maximum level which caused the accelerated sorption rate. Here, this spiked phase is clarified via the fast gas molecule uptake due to the significant concentration difference which pushes the molecules of the gas into the liquid phase of HDES. Likewise, the sudden gas pressure applying to the liquid HDES may cause DES swelling leading to more gas diffusion to the bulk of DES. Nevertheless, when the pressure achieves the state of equilibrium, the excess absorbed gas begins to desorb. During the initial stages of absorption, gas molecule absorption happens at the surface of the DES. When the surface layer reaches the saturation stage, the system redistributes the absorbed gas molecules to the bulk of DES. This redistribution causes a transient excess absorption stage before attaining the equilibrium and stability stage. Consequently, the explained process is responsible for the observed overshooting absorption.

### 3.3 Molecular Dynamics (MD) Simulations

Classical MD simulation using MDynaMix v.5.2 molecular modeling package^61^ was used to study the CO_2_ and N_2_ solubility in DES systems (Table S1) at 293 K and 1 bar and for the force fields included in Table S2. The DES contained HBD linoleic and HBA camphor, citral, and piperitone (CAM, CIT, and PIP, respetively) with the gas (CO_2_, N_2_) (Figure 12). In this work, the mole fractions of HBAs and HBD were treated depending on the determined molar mixing ratios (e.g., 1:1 M mixing ratio corresponds to *x*HBA = 0.5 and *x*HBD = 0.5). The concentration of the gas ratio will be up to 0.5 (*x*CO_2_, *N*_2_ = 0.05, 0.1, 0.3, and 0.5). Force field parameters were obtained from the SwissParam database (Merck Molecular Force Field) with atomic charges obtained from ChelpG-DFT-optimized structures for isolated monomers (HBAs and HBD).^55–57^ MD initial cubic simulation boxes were built with Packmol program.^65^ All simulations were carried out using periodic boundary conditions in the three space directions applying a three-step consecutive procedure: (i) 1 ns *NVT* simulations at 293 K, (ii) 10 ns *NPT* equilibration step at 293 K and 1 bar, and (iii) 5 ns *NPT* production simulations at 293 K and 1 bar. Equilibrium was assured by monitoring the time evolution of total potential energy and for selected properties such as density. The Nose–Hoover method was used for pressure and temperature control, with 30 and 1000 ps as the time constants for the thermostat and barostat, respectively. The Tuckerman–Berne double time step algorithm^66^ (with long- and short-time steps of 1 and 0.1 fs, respectively) was applied for solving the equations of motion. The Ewald method^67^ (1.5 nm for the cut-off radius) was applied for handling Coulombic interactions. Intermolecular interactions were described with the Lennard-Jones potential with a 15 Å cutoff distance and Lorentz–Berthelot mixing rules for cross terms.^68^ The visualization, analysis, and postprocessing of MD trajectories were carried out using VMD^69^ and TRAVIS.^70^

**Figure 12:**
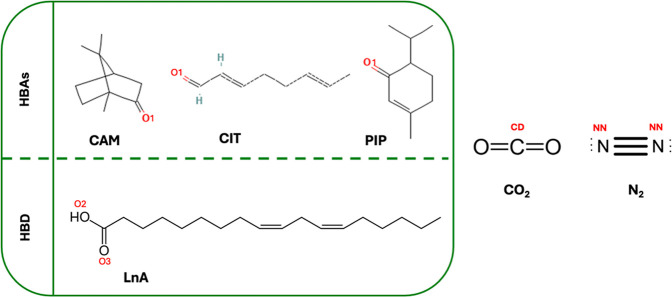
Molecular structures of compounds used in this work for the considered DES (HBAs/HBD), CO_2_ and N_2_.

Regarding real flue gas absorption, we currently lack the necessary experimental setup to measure absorption under such conditions. Our future works aim to enhance experimental capabilities in this direction. Nevertheless, we believe the results obtained from our CO_2_ absorption studies provide significant insights into the potential of these DES systems for industrial applications.^62–64^

MD simulations were performed for the DES and DES + CO_2_/N_2_ mixtures. The MD study provided information about the nanoscopic properties of the studied DES (1:1) and DES (1:1) + CO_2_/N_2_ mixtures to analyze the behavior of these gases in the studied DES, as well as the structural changes in the DES upon gas absorption for gas capture operations. Force field parametrizations were validated first by comparison of the experimental and predicted by using MD density values (ρ) obtained for the studied DES at 293 K and 1 bar, which were, respectively, 0.9101 g·cm^–3^ for [CAM]/[LnA]; 0.8838 g·cm^–3^ for [CIT]/[LnA]; and 0.0.901 for [PIP]/[LnA]. It should be noted that the experimental density (uncertainty ±1 × 10^–4^ g·cm^–3^) was measured with an Anton Paar DMA1001 vibrating tube densimeter, with Peltier element controlling temperature measured to ±0.01 K.

For initial characterization of the hydrogen bonds in the bulk mixtures, Radial Distribution Functions (RDFs) for HBA–HBD, HBA–HBA, and HBD–HBD DES + CO_2_/N_2_ mixtures are reported in Figure 13 and 14 for all the possible donor–acceptor sites as a function of the DES content. These HBA–HBD RDFs show a first intense peak at 2.75 Å for the interaction between the oxygen atom in CAM/CIT/PIP and the corresponding hydroxyl group in linoleic acid (O1–O2 site) and also a first narrow and less intense peak at 3.12 Å for the interaction between the oxygen atom in CAM, CIT, PIP, and the corresponding carbonyl group in linoleic acid (O1–O3 site), which confirms the development of hydrogen bonding. The strength of the HBA–HBD interactions was quantified by the intermolecular interaction energies, E_inter_, Figure 21. The reported results (blue) agree with those for the number of hydrogen bonds per molecule.

**Figure 13:**
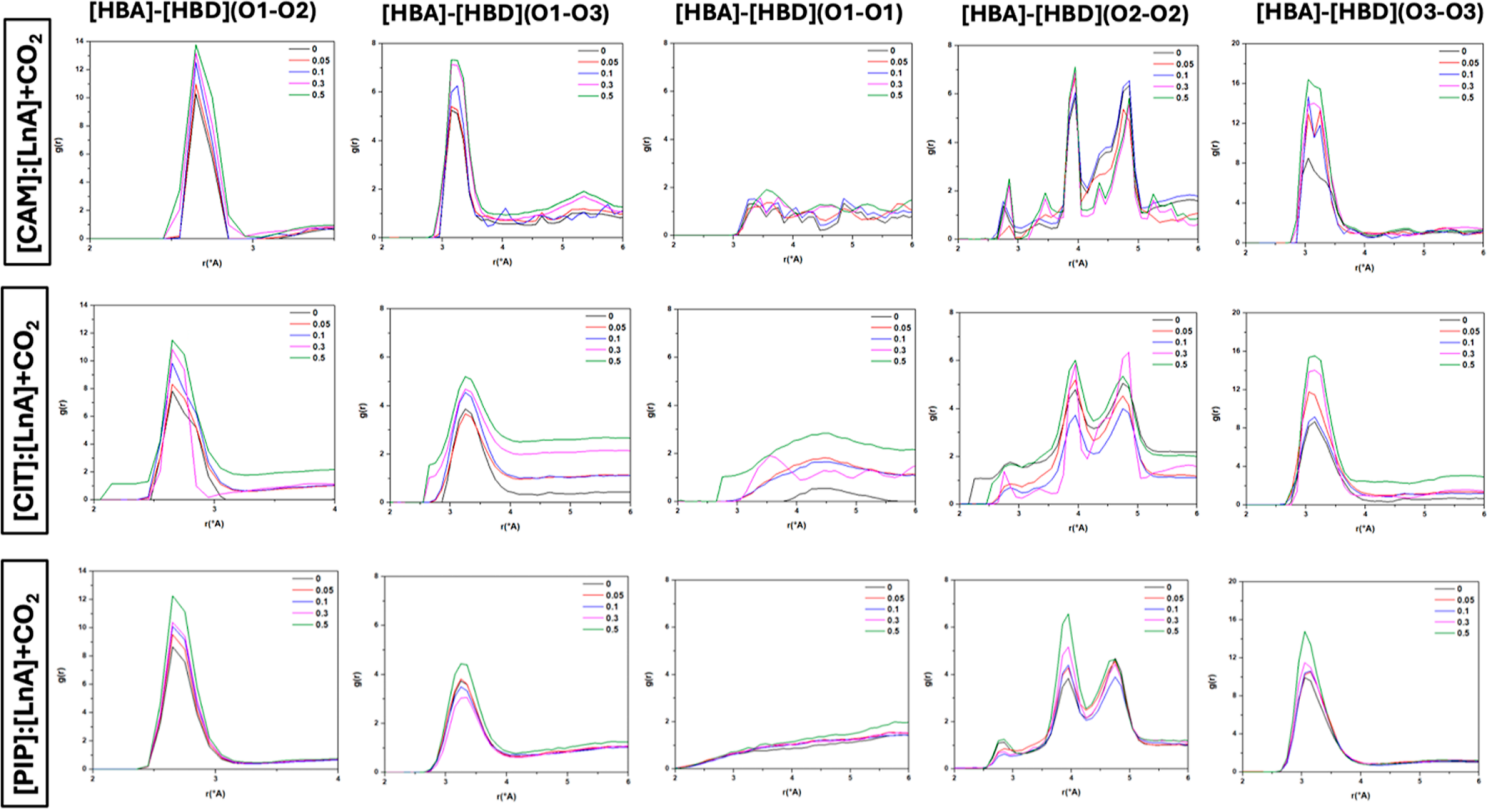
Site–site radial distribution functions, *g*(*r*), for [HBAs]/[HBD], [HBA]/[HBA], and [HBD]/[HBD] sites in the reported DES (1:1) + CO_2_ systems at *x*_CO_2__ = 0/0.05/0.1/0.3/0.5 (CO_2_ effect) (atom labeling is shown in Figure 12).

**Figure 14:**
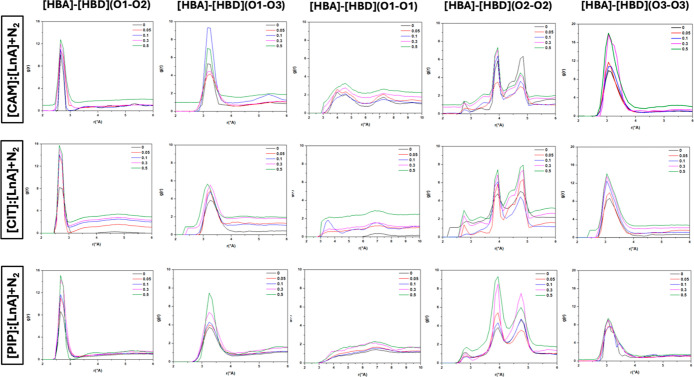
Site–site radial distribution functions, *g*(*r*), for [HBA]/[HBD], [HBA]/[HBA], and [HBD]/[HBD] sites in the reported DES (1:1) + N_2_ systems at *x*_N_2__ = 0/0.05/0.1/0.3/0.5 (N_2_ effect) (atom labeling is shown in Figure 12).

Upon further analysis of our radial distribution functions, particularly for [HBA–HBA], [HBD–HBD] (O1–O1) (O2–O2) and (O3–O3) were studied. For HBD–HBD RDFs, the intense peaks (especially between the oxygen atom of the carbonyl group in linoleic acid, the O3–O3 site) confirm that HBD molecules are self-associated by hydrogen bonding. In the [CAM]/[LnA] + CO_2_ system, we observe a distinct correlation between the magnitude of the first peak and the gas concentration. This correlation indicates that the intensity of the RDF peaks is directly influenced by the CO_2_ concentration, underscoring the importance of the gas concentration in modulating molecular interactions in the system.

In our analysis of the HBD–HBD interactions, particularly between the O3 and O3 sites, the CAM–LnA system exhibited characteristics strongly indicative of hydrogen bonding. The observed trends in bond distances and RDF peaks for the CAM–LnA system consistently support this interpretation. On the other hand, while the CIT–LnA and PIP–LnA systems also showed trends in bond distances and RDF peaks that were in line with our observations, the nature and strength of the HBD–HBD interactions in these systems were less definitive in the PIP–LnA system.

The RDF peaks for the possible site CO_2_ mixtures and for N_2_ mixtures with HBA and HBD are reported in Figures 15 and 16, the effect of CO_2_ and N_2_ concentration on the peak intensity, a peak at 3.15 Å for O1-CD sites in the CIT and PIP, while more intense peaks in the CAM case at 5.1 Å. This structure is maintained upon the increase of CO_2_ concentration, with an increase in the intensity of RDF peaks because of a higher concentration of CO_2_ molecules. For [CIT]/[LnA] DES, [HBD]–CO_2_ RDFs show a similar behavior to [PIP]/[LnA] DES, a first peak at 3.15 Å for the O2-CD and O3-CD sites but with less intense peaks in the O2-CD than for the O3-CD.

**Figure 15:**
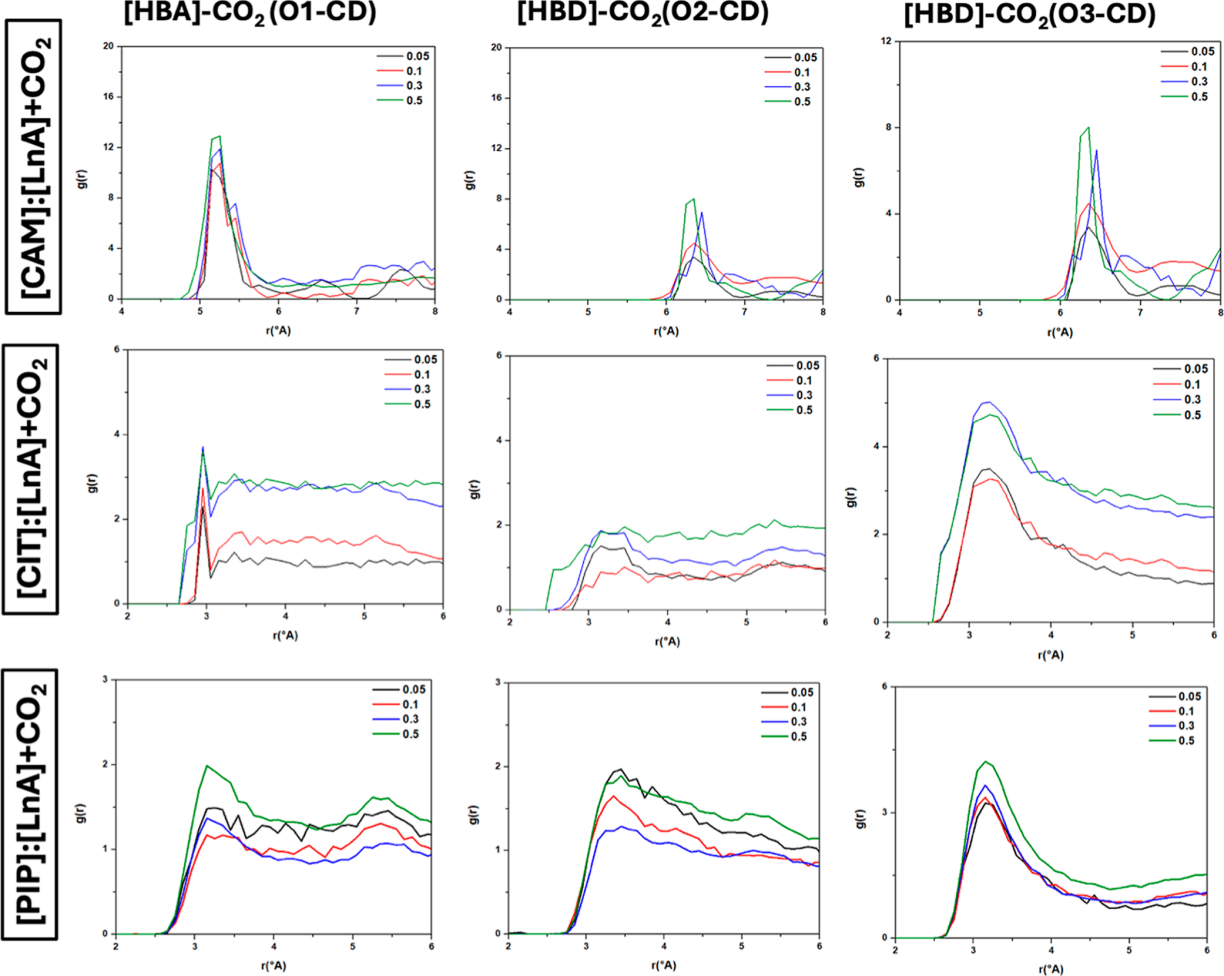
Site–site radial distribution functions, *g*(*r*), for [HBA]/CO_2_ and [HB]/CO_2_ sites in the reported DES (1:1) + CO_2_ systems at *x*_CO_2__ = 0.05/0.1/0.3/0.5 (atom labeling is shown in Figure 12).

**Figure 16:**
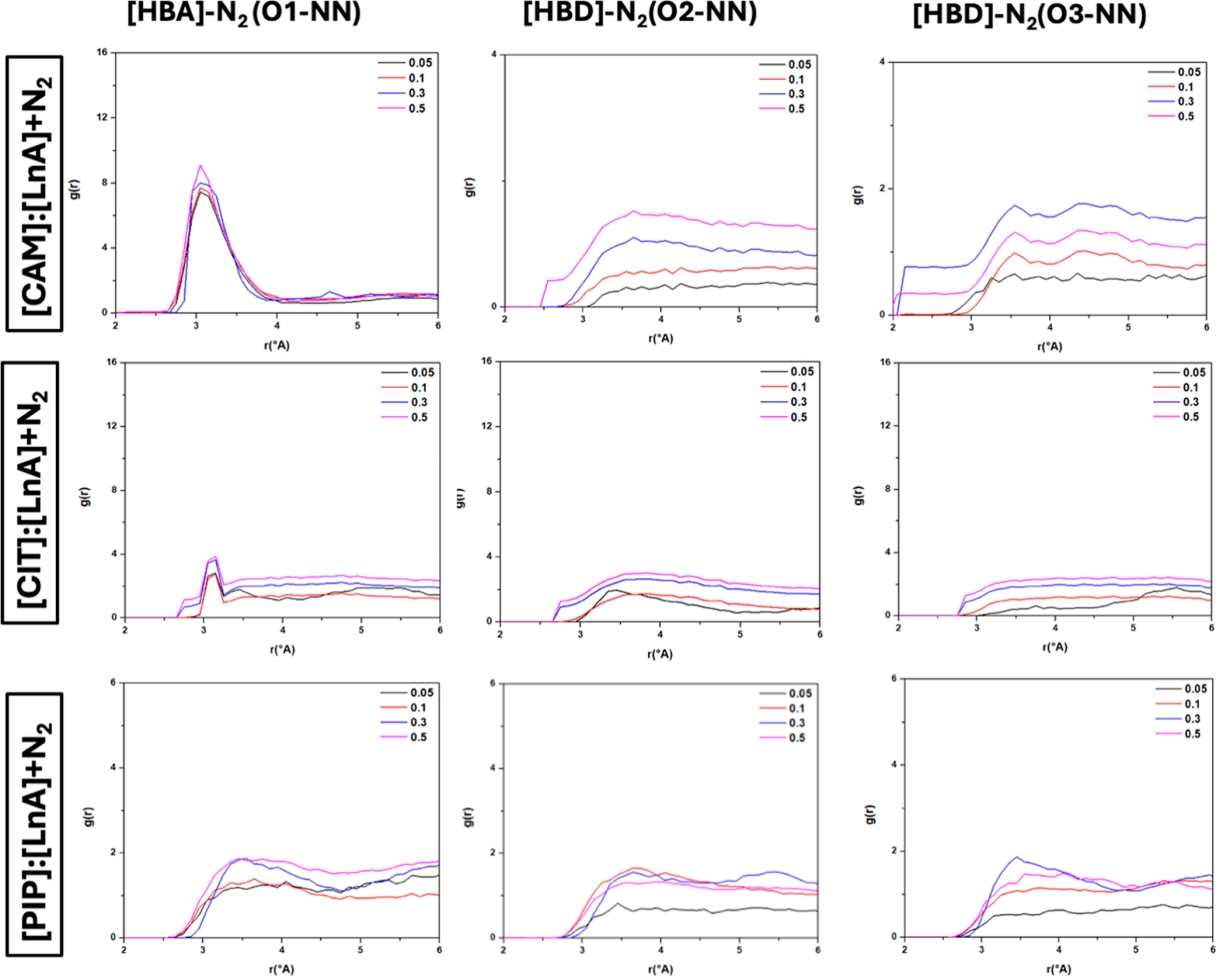
Site–site radial distribution functions, *g*(*r*), for [HBA]/N_2_ and [HBD]/N_2_ sites in the reported DES (1:1) + N_2_ systems at *x*_N_2__ = 0.05/0.1/0.3/0.5 (atom labeling is shown in Figure 12).

For [CAM]/[LnA] DES, [HBD]–CO_2_ RDFs show higher intensity for the O2-CD and O3-CD at distance 6.2. However, RDFs for [HBA]–CO_2_ and [HBD]–CO_2_ pairs show the trend of DES compounds interacts with CO_2_ molecules. It shows a less strong trend (especially the [LnA] O2 site) interacting with CO_2_ molecules compared with [LnA]­O3. O2 seems to interact strongly with the HBA, while O3 tends to build a strong interaction with CO_2_. The distribution of N_2_ molecules around DES components was also studied with the corresponding RDFs reported in Figure 16. These results also indicate how N_2_ molecules are distributed near the O (HBA)–COOH (HBD) region. For [CAM]/[LnA] DES, [HBA]–N_2_ and [HBD]–N_2_ RDFs show a first peak at 3.25 Å and a less intense peak at 3.20 for [CIT]/[LnA] DES and [PIP]/[LnA] DES.

In all DESs, the intensity of the RDFs for [HBA]–N_2_ and [HBD]–N_2_ pairs confirms the trend of the DES compounds to interact with N_2_ molecules. [CAM]/[LnA] shows a stronger trend (especially [CAM]) to interact with N_2_ molecules compared to [CIT]/[LnA] and [PIP]/[LnA].

The extension of hydrogen bonding was quantified using a geometrical criterion considering 3.5 Å and 60° for the donor–acceptor separation and angle, respectively. Results for the number of hydrogen bonds per molecule, *N*_HB_, for HBA–HBD interactions are reported in Figure 17 for DES + CO_2_ mixtures as a function of *x*_CO_2__, confirming the development of HBA–HBD associations by hydrogen bonding with a minor concentration impact. The solvation numbers, *N*, reported in Figure S6, confirm that this mechanism of interaction of RDF is maintained in the studied concentration ranges, thus the available space around the polar sites of the considered NADES allows proper fitting of the increasing number of CO_2_/N_2_ molecules.

**Figure 17:**
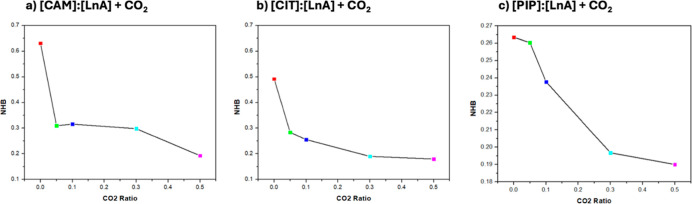
Average number of hydrogen bonds per HBD molecule, NH, for [HBA]/[HBD] interactions (O1–O2) in the reported DES (1:1) + CO_2_ from MD simulations at 293 K and 1 bar as a function of *x*_CO_2__ (atom labeling as in Figure 12).

The extension of hydrogen bonds is larger for [CAM]/[LnA] than for [CIT]/ [LnA] and [PIP]/[LnA], so [CAM]/[LnA] (1:1) is more efficient in the CO_2_ absorption operation compared to [CIT]/[LnA] and [PIP]/[LnA] systems. Likewise, results for the number of hydrogen bonds per molecule, *N*_HB_, for HBA–HBD interactions reported in Figure 18 for DES + N_2_ mixtures as a function of *x*_N_2__ also confirm the development of HBA–HBD associations by hydrogen bonding with a minor concentration effect. However, in the case of N_2_ mixtures, the extension of hydrogen bonds is larger for both [CAM]/[LnA] and [CIT]/[LnA] than for [PIP]/[LnA].

**Figure 18:**
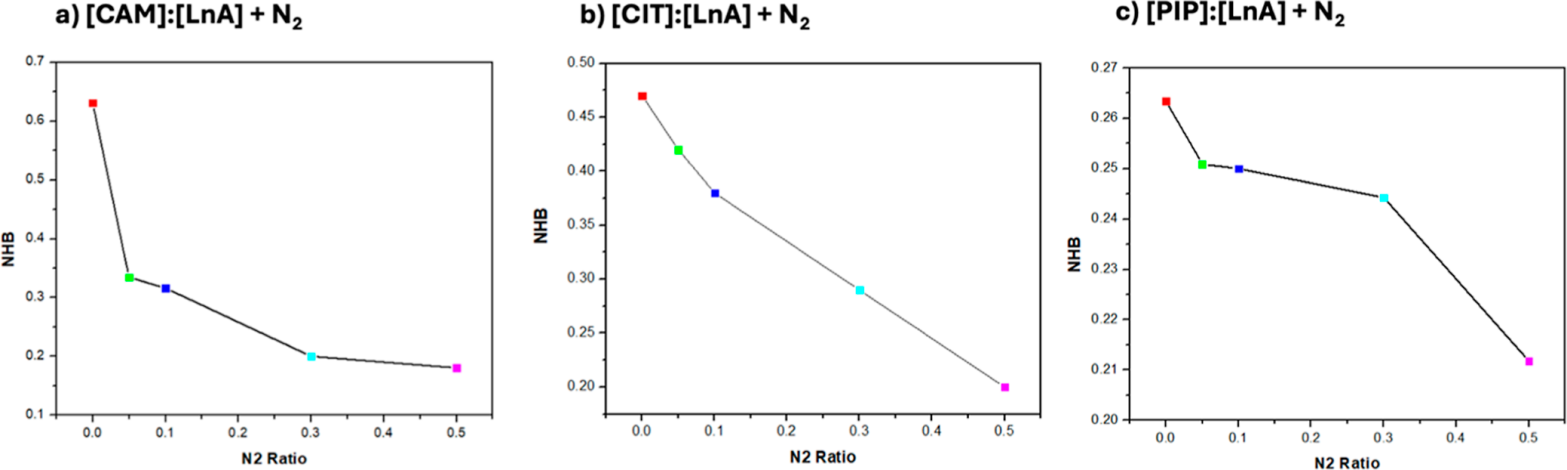
Average number of hydrogen bonds per HBD molecule, *N*_H_, for [HBA]/[HBD] interactions (O1–O2) in the reported DES (1:1) + N_2_ from MD simulations at 293 K and 1 bar as a function of *x*_N_2__ (atom labeling is shown in Figure 12).

The trend to develop hydrogen bonding is confirmed through the Spatial Distribution Functions (SDFs) as reported in Figures 19 and 20, which illustrates distinct and competitive H-bonding patterns with highly localized distributions of LnA molecules (in blue) around the HBA sites, becoming less dense as *x*_CO_2_/N_2__ increases. Red regions around the O1 of the HBAs reflect the CO_2_ solubility interaction scenario where HBAs contest with CO_2_ for bonding sites (Figure 19). Green regions around the O1 of HBAs suggest the hydrogen bonds with the N_2_ (Figure 20). Figure 21 shows the intermolecular interaction energies for the different interaction sites in the reported DES (1:1) + CO_2_/N_2_ from MD simulations at 293 K and 1 bar as a function of *x*_CO_2__/*x*_N_2__.

**Figure 19:**
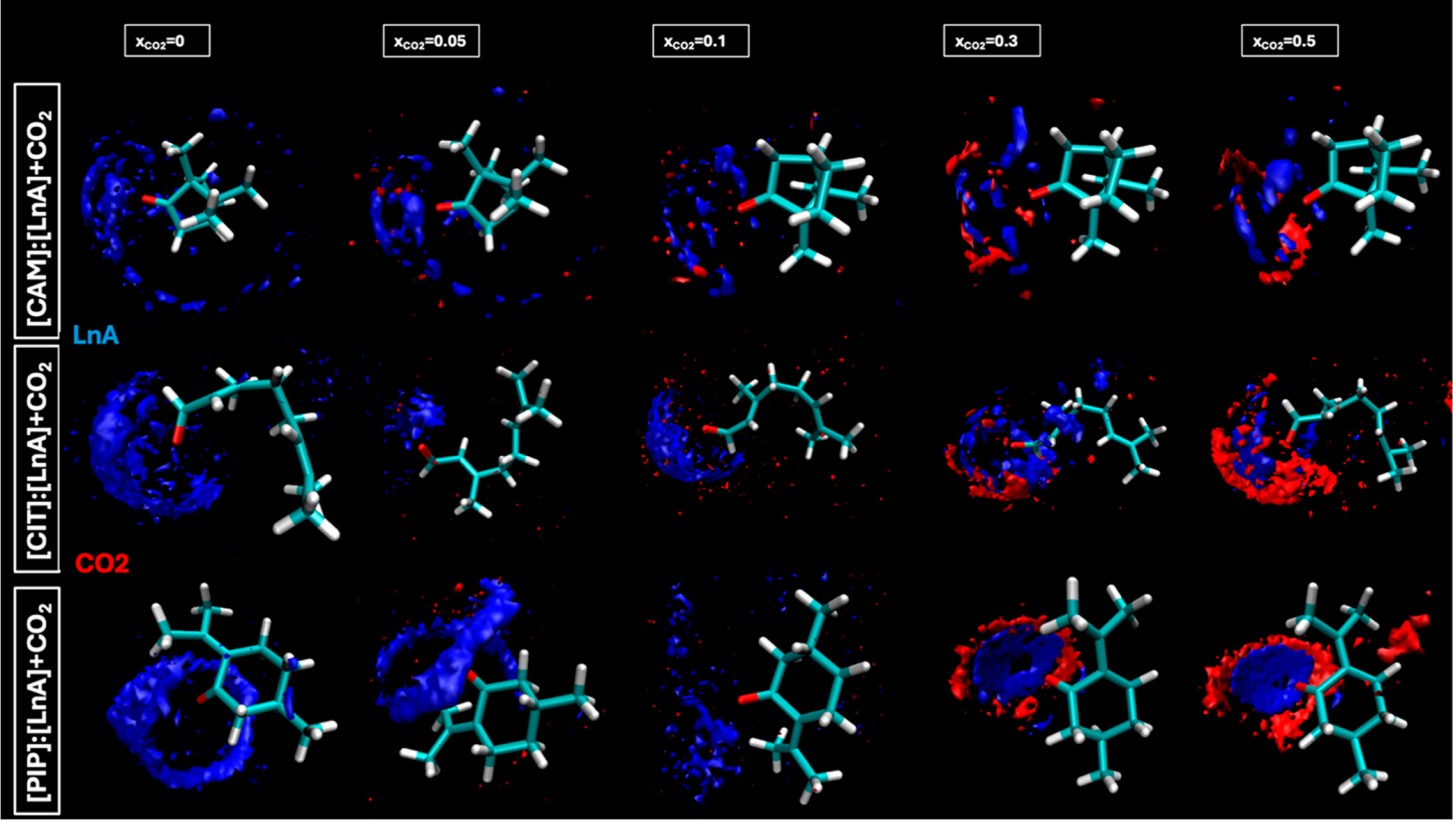
Spatial distribution functions, SDFs, of the corresponding centers-of-mass of LnA and CO_2_ around central [HBA] molecules for the reported DES (1:1) + CO_2_ as a function of *x*_CO_2__.

**Figure 20:**
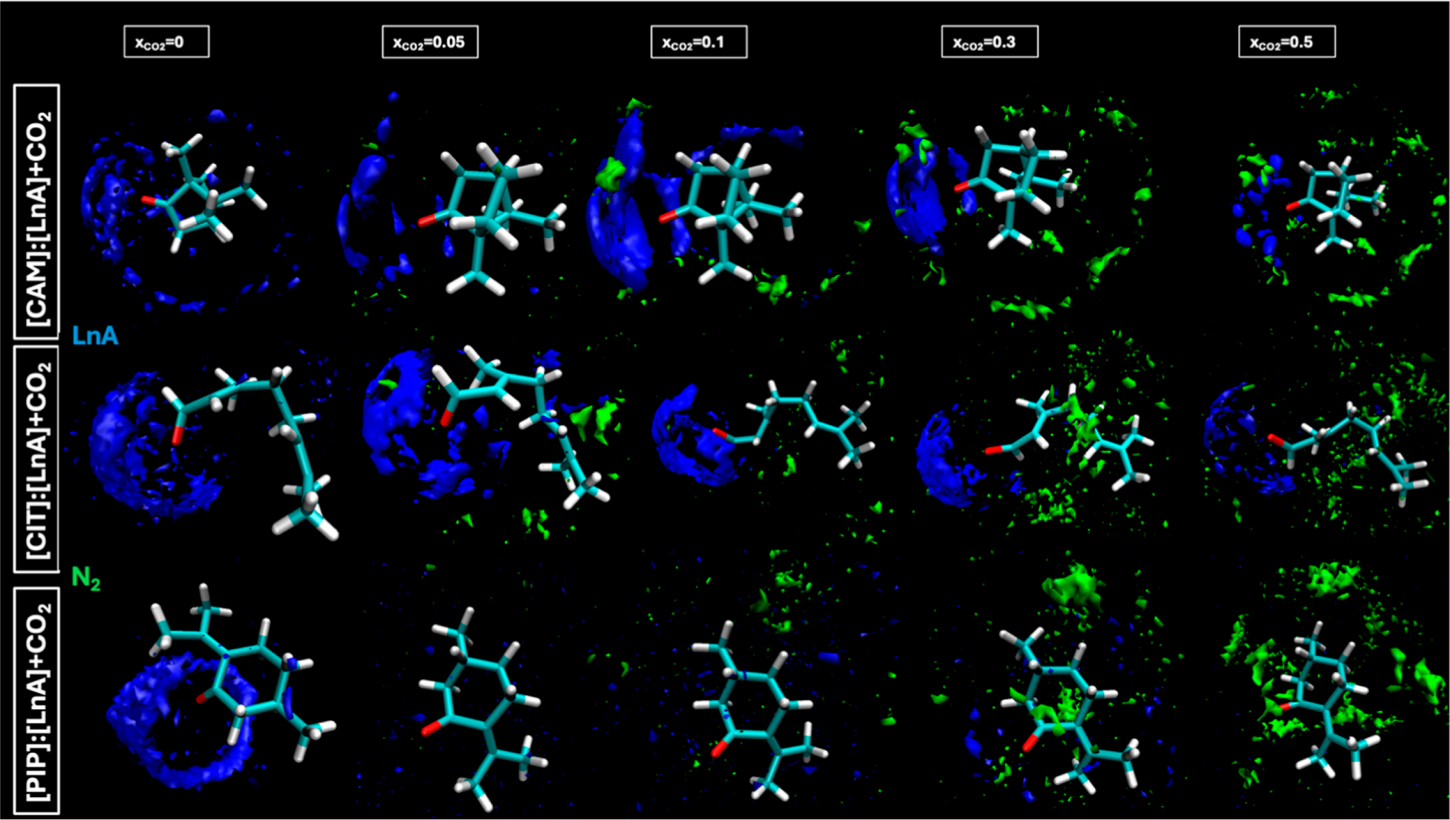
Spatial distribution functions, SDFs, of the corresponding centers-of-mass of LnA and N_2_ around central [HBA] molecules for the reported DES (1:1) + N_2_ as a function of *x*_N_2__.

**Figure 21:**
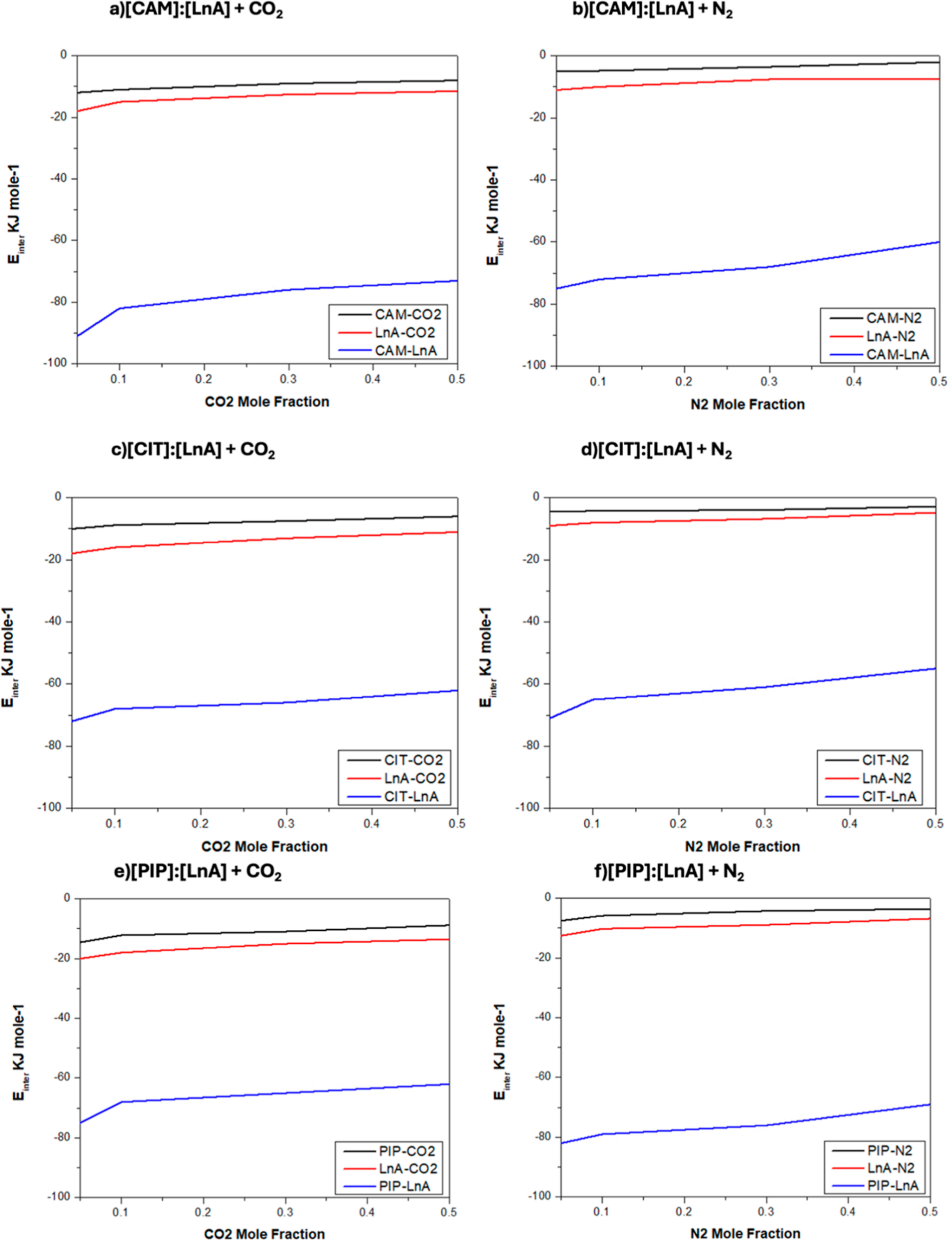
Intermolecular interaction energies, E_inter_ (sum of Lennard-Jones and Coulombic contributions), for the different interaction sites in the reported DES (1:1) + CO_2_/N_2_ from MD simulations at 293 K and 1 bar as a function of *x*_CO_2__/*x*_N_2__.

All in all, as a comprehensive summary of the mechanistic insight into gas absorption, the process in the studied HDES systems is primarily governed by physisorption, driven by weak van der Waals and electrostatic interactions. The structural flexibility of the HDES network allows gas, especially CO_2_ molecules, to localize in specific nanocavities or interact with functional groups, such as hydroxyl and carbonyl moieties, via hydrogen bonding, as characterized through molecular simulations. This conclusion is supported by three key observations: (1) FTIR spectra before and after gas exposure show no formation of new chemical bonds, indicating nonreactive, physical uptake; (2) molecular dynamics simulations reveal preferential CO_2_ accumulation in structured zones of the DES matrix, stabilized by noncovalent forces; and (3) the complete regeneration of the DESs after simple vacuuming without heating further confirms the reversible, nonchemical nature of the sorption. CO_2_ exhibits stronger interactions due to its quadrupole moment and higher polarizability, while N_2_ shows a more dispersed, less structured interaction profile. These findings collectively provide molecular-level insights into the observed gas selectivity and the superior CO_2_ absorption capacity of the studied systems.

## 4 Conclusions

This study highlights the effectiveness of the CAM–LnA, CIT–LnA, and PIP–LnA HDES systems for CO_2_ capture, showing significant CO_2_ absorption behavior under varying pressures. Among the systems, CIT–LnA displayed the highest absorption efficiency (approximately 5.2% greater than those of the other systems), particularly at low pressures. All systems demonstrated increased CO_2_ absorption rates with pressure, with CAM–LnA performing best at high pressures and CIT–LnA showing faster initial kinetics.

The molecular simulations provided crucial insights into the gas–DES interactions, particularly through radial distribution functions (RDFs) and spatial distribution functions (SDFs). These analyses revealed a strong affinity for CO_2_ and N_2_ molecules to localize around the HBA–HBD regions of the DES, with CAM–LnA demonstrating the strongest interactions, followed by CIT–LnA. The hydrogen bonding between gas molecules and DES components, as indicated by hydrogen-bond counts, further confirmed the affinity of these systems for CO_2_ and N_2_. Changes in the gas concentration led to shifts in the spatial arrangement around the polar regions of the DES, contributing to the absorption behavior observed experimentally.

These HDES systems provide a sustainable alternative to conventional CO_2_ capture agents, offering the potential for lower operational costs and simpler regeneration processes. Future work should explore the scalability of these systems at higher pressures and their applications in industrial carbon capture processes.

## Supplementary Material



## Notes

Actual high-pressure gas sorption equipment; VFT-model fitting of viscosity data of the studied NADES; regular residual of VFT-model fitting of viscosity of the studied NADES; impact of LnA material purity on the CO_2_ absorption capacity; hydrophobicity of the prepared DES systems; mass spectrum for linoleic acid; mass spectrum for piperitone; mass spectrum for camphor; mass spectrum for citral; solvation numbers (*N*) for [HBA]/CO_2_/N_2_ and [HBD]/CO_2_/N_2_ sites in the reported NADES (1:1) + CO_2_/N_2_ as a function of *x*_CO_2__ and *x*_N_2__; systems considered for *NPT* molecular dynamics simulations; and force field parametrization for compounds studied in MD simulations (PDF)

## References

[1] Le
Quéré C., Jackson R. B., Jones M. W., Smith A. J. P., Abernethy S., Andrew R. M., De-Gol A. J., Willis D. R., Shan Y., Canadell J. G., Friedlingstein P., Creutzig F., Peters G. P. (2020). Temporary Reduction in Daily Global
CO2 Emissions during the COVID-19 Forced Confinement. Nat. Clim. Change.

[2] Peters G. P., Andrew R. M., Canadell J. G., Friedlingstein P., Jackson R. B., Korsbakken J. I., Le Quéré C., Peregon A. (2020). Carbon Dioxide Emissions Continue to Grow amidst Slowly
Emerging Climate Policies. Nat. Clim. Change.

[3] Karadas F., Atilhan M., Aparicio S. (2010). Review on
the Use of Ionic Liquids
(ILs) as Alternative Fluids for CO2 Capture and Natural Gas Sweetening. Energy Fuels.

[4] Zeng S., Zhang X., Bai L., Zhang X., Wang H., Wang J., Bao D., Li M., Liu X., Zhang S. (2017). Ionic-Liquid-Based CO2 Capture Systems: Structure, Interaction and
Process. Chem. Rev..

[5] Huy P. Q., Sasaki K., Sugai Y., Kiga T., Fujioka M., Adachi T. (2009). Effects of SO2 and
pH Concentration on CO2 Adsorption
Capacity in Coal Seams for CO2 Sequestration With Considerations for
Flue Gas From Coal-Fired Power Plants. J. Can.
Pet. Technol..

[6] Salmón I. R., Cambier N., Luis P. (2018). CO2 Capture
by Alkaline Solution
for Carbonate Production: A Comparison between a Packed Column and
a Membrane Contactor. Appl. Sci..

[7] Choi Y.-S., Im J., Jeong J. K., Hong S. Y., Jang H. G., Cheong M., Lee J. S., Kim H. S. (2014). CO2 Absorption and Desorption in
an Aqueous Solution of Heavily Hindered Alkanolamine: Structural Elucidation
of CO2-Containing Species. Environ. Sci. Technol..

[8] Barzagli F., Di Vaira M., Mani F., Peruzzini M. (2012). Improved Solvent
Formulations for Efficient CO2 Absorption and Low-Temperature Desorption. ChemSusChem.

[9] Zheng L., Matin N. S., Landon J., Thomas G. A., Liu K. (2016). CO2 Loading-Dependent
Corrosion of Carbon Steel and Formation of Corrosion Products in Anoxic
30wt.% Monoethanolamine-Based Solutions. Corros.
Sci..

[10] Poste A. E., Grung M., Wright R. F. (2014). Amines
and Amine-Related Compounds
in Surface Waters: A Review of Sources, Concentrations and Aquatic
Toxicity. Sci. Total Environ..

[11] Chowdhury F. A., Goto K., Yamada H., Matsuzaki Y. (2020). A Screening
Study of Alcohol Solvents for Alkanolamine-Based CO2 Capture. Int. J. Greenh. Gas Control.

[12] Reynolds A. J., Verheyen T. V., Adeloju S. B., Meuleman E., Feron P. (2012). Towards Commercial
Scale Postcombustion Capture of CO2 with Monoethanolamine Solvent:
Key Considerations for Solvent Management and Environmental Impacts. Environ. Sci. Technol..

[13] Rao A. B., Rubin E. S. (2002). A Technical, Economic, and Environmental Assessment
of Amine-Based CO2 Capture Technology for Power Plant Greenhouse Gas
Control. Environ. Sci. Technol..

[14] Davis J., Rochelle G. (2009). Thermal Degradation
of Monoethanolamine at Stripper
Conditions. Energy Procedia.

[15] Zubeir L. F., van Osch D. J. G. P., Rocha M. A. A., Banat F., Kroon M. C. (2018). Carbon
Dioxide Solubilities in Decanoic Acid-Based Hydrophobic Deep Eutectic
Solvents. J. Chem. Eng. Data.

[16] Francisco M., van den Bruinhorst A., Zubeir L. F., Peters C. J., Kroon M. C. (2013). A New Low
Transition Temperature Mixture (LTTM) Formed by Choline Chloride+lactic
Acid: Characterization as Solvent for CO2 Capture. Fluid Phase Equilib..

[17] Song C., Pan W., Srimat S. T., Zheng J., Li Y., Wang Y.-H., Xu B.-Q., Zhu Q.-M. (2004). Tri-Reforming of Methane over Ni
Catalysts for CO2 Conversion to Syngas With Desired H2/CO Ratios Using
Flue Gas of Power Plants Without CO2 Separation. Stud. Surf. Sci. Catal..

[18] Xu X., Song C., Miller B. G., Scaroni A. W. (2005). Influence of Moisture
on CO2 Separation from Gas Mixture by a Nanoporous Adsorbent Based
on Polyethylenimine-Modified Molecular Sieve MCM-41. Ind. Eng. Chem. Res..

[19] van
Osch D. J. G. P., Zubeir L. F., van den Bruinhorst A., Rocha M. A. A., Kroon M. C. (2015). Hydrophobic Deep Eutectic Solvents
as Water-Immiscible Extractants. Green Chem..

[20] Dietz C. H. J. T., van Osch D. J. G. P., Kroon M. C., Sadowski G., van Sint Annaland M., Gallucci F., Zubeir L. F., Held C. (2017). PC-SAFT Modeling
of CO2 Solubilities in Hydrophobic Deep Eutectic Solvents. Fluid Phase Equilib..

[21] Cao J., Yang M., Cao F., Wang J., Su E. (2017). Tailor-Made
Hydrophobic Deep Eutectic Solvents for Cleaner Extraction of Polyprenyl
Acetates from Ginkgo Biloba Leaves. J. Clean.
Prod..

[22] Al-Bodour A., Alomari N., Gutiérrez A., Aparicio S., Atilhan M. (2024). Exploring
the Thermophysical Properties of Natural Deep Eutectic Solvents for
Gas Capture Applications: A Comprehensive Review. Green Chem. Eng..

[23] Zamora L., Benito C., Gutiérrez A., Alcalde R., Alomari N., Bodour A. A., Atilhan M., Aparicio S. (2022). Nanostructuring and
Macroscopic Behavior of Type V Deep Eutectic Solvents Based on Monoterpenoids. Phys. Chem. Chem. Phys..

[24] Goswami A., Rahman S. N. R., Sree A., Shunmugaperumal T. (2024). Solubility
of Cinnarizine in Natural Deep Eutectic Solvent (Camphor + Menthol)
and Correlation with Different Solubility Models. Fluid Phase Equilib..

[25] Lu W.-C., Huang D.-W., Wang C.-C. R., Yeh C.-H., Tsai J.-C., Huang Y.-T., Li P.-H. (2018). Preparation,
Characterization, and
Antimicrobial Activity of Nanoemulsions Incorporating Citral Essential
Oil. J. Food Drug Anal..

[26] Xiao L., Wei Y., Liu X., Wang B., Chen Y., Cui Z. (2024). Extractive
Separation of Citral and Limonene with Quaternary Ammonium/Alkanediol
Deep Eutectic Solvents: An Experimental and Mechanistic Study. J. Mol. Liq..

[27] Wei S., Xu Q., Pei S., Lv Y., Lei Y., Zhang S., zhai H., Hu Y. (2024). Unraveling
the Antifungal and Anti-Aflatoxin
B1Mechanisms of Piperitone on Aspergillus Flavus. Food Microbiol..

[28] Sawant N., Alomari N., Aguilar J., Velez M., Lizardo M., Caceres S., Ogando R., Garcia C., Maletta A., Hossen A., Gutierrez A., Springstead J., Aparicio S., Atilhan M. (2024). Enhanced Water Purification
with
Hydrophobic Natural Deep Eutectic Solvents Focused on Phenolic Compounds
Removal. J. Water Process Eng..

[29] Maletta A., Gutiérrez A., Jian Tan P., Springstead J., Aparicio S., Atilhan M. (2023). Separation of Phenolic Compounds
from Water by Using Monoterpenoid and Fatty Acid Based Hydrophobic
Deep Eutectic Solvents. J. Mol. Liq..

[30] Klamt A. (1995). Conductor-like
Screening Model for Real Solvents: A New Approach to the Quantitative
Calculation of Solvation Phenomena. J. Phys.
Chem..

[31] Klamt A., Eckert F. (2000). COSMO-RS: A Novel and Efficient Method for the a Priori
Prediction of Thermophysical Data of Liquids. Fluid Phase Equilib..

[32] Al-Bodour A., Alomari N., Gutiérrez A., Aparicio S., Atilhan M. (2022). High-Pressure
Carbon Dioxide Solubility in Terpene Based Deep Eutectic Solvents. J. Environ. Chem. Eng..

[33] Span R., Wagner W. (1996). A New Equation of State for Carbon Dioxide Covering
the Fluid Region from the Triple-Point Temperature to 1100 K at Pressures
up to 800 MPa. J. Phys. Chem. Ref. Data.

[34] Al-Bodour A., Alomari N., Aparicio S., Atilhan M. (2023). A Comprehensive Study
on Carbon Capture Potential of Lactic Acid Based Deep Eutectic Solvents
at Wide Process Conditions. J. Mol. Liq..

[35] França J. M. P., Nieto de Castro C. A., Lopes M. M., Nunes V. M. B. (2009). Influence
of Thermophysical Properties of Ionic Liquids in Chemical Process
Design. J. Chem. Eng. Data.

[36] Schroeder, M. ; Martin, M. Chemical Thermodynamics for Industry; RSC, 2004.

[37] Ghaedi H., Ayoub M., Sufian S., Shariff A. M., Murshid G., Hailegiorgis S. M., Khan S. N. (2017). Density, Excess and Limiting Properties
of (Water and Deep Eutectic Solvent) Systems at Temperatures from
293.15K to 343.15K. J. Mol. Liq..

[38] Halder A. K., Haghbakhsh R., Voroshylova I. V., Duarte A. R. C., Cordeiro M. N. D. S. (2021). Density
of Deep Eutectic Solvents: The Path Forward Cheminformatics-Driven
Reliable Predictions for Mixtures. Molecules.

[39] García G., Aparicio S., Ullah R., Atilhan M. (2015). Deep Eutectic Solvents:
Physicochemical Properties and Gas Separation Applications. Energy Fuels.

[40] Nowosielski B., Jamrógiewicz M., Łuczak J., Śmiechowski M., Warmińska D. (2020). Experimental and Predicted Physicochemical Properties
of Monopropanolamine-Based Deep Eutectic Solvents. J. Mol. Liq..

[41] Abbott A. P., Ahmed E. I., Harris R. C., Ryder K. S. (2014). Evaluating Water
Miscible Deep Eutectic Solvents (DESs) and Ionic Liquids as Potential
Lubricants. Green Chem..

[42] Zhou Q., Wang L.-S., Chen H.-P. (2006). Densities and Viscosities of 1-Butyl-3-Methylimidazolium
Tetrafluoroborate + H2O Binary Mixtures from (303.15 to 353.15) K. J. Chem. Eng. Data.

[43] DiGuilio R. M., Lee R. J., Schaeffer S. T., Brasher L. L., Teja A. S. (1992). Densities
and Viscosities of the Ethanolamines. J. Chem.
Eng. Data.

[44] Gao Q., Jian Z. (2020). Fragility and Vogel-Fulcher-Tammann
Parameters near Glass Transition
Temperature. Mater. Chem. Phys..

[45] Ikeda M., Aniya M. (2010). Bond Strength—Coordination
Number Fluctuation Model of Viscosity:
An Alternative Model for the Vogel-Fulcher-Tammann Equation and an
Application to Bulk Metallic Glass Forming Liquids. Materials.

[46] Alomari N., Al-Bodour A., Khai Liew S., Gutiérrez A., Aparicio S., Atilhan M. (2023). Exploiting Monoterpenoids in Type
V Deep Eutectic Solvents: A Combined High-Pressure Experiments and
Theoretical Approach for Enhanced Carbon Dioxide and Nitrogen Absorption. J. Mol. Liq..

[47] Chen Y., Chen W., Fu L., Yang Y., Wang Y., Hu X., Wang F., Mu T. (2019). Surface Tension of 50 Deep Eutectic
Solvents: Effect of Hydrogen-Bonding Donors, Hydrogen-Bonding Acceptors,
Other Solvents, and Temperature. Ind. Eng. Chem.
Res..

[48] Zhang Q., De Oliveira Vigier K., Royer S., Jérôme F. (2012). Deep Eutectic
Solvents: Syntheses, Properties and Applications. Chem. Soc. Rev..

[49] González-Rivera J., Pelosi C., Pulidori E., Duce C., Tiné M. R., Ciancaleoni G., Bernazzani L. (2022). Guidelines for a Correct Evaluation
of Deep Eutectic Solvents Thermal Stability. Curr. Res. Green Sustain. Chem..

[50] Nandiyanto A. B. D., Oktiani R., Ragadhita R. (2019). How to Read
and Interpret FTIR Spectroscope
of Organic Material. Indones. J. Sci. Technol..

[51] Farooq M. Q., Abbasi N. M., Anderson J. L. (2020). Deep Eutectic
Solvents in Separations:
Methods of Preparation, Polarity, and Applications in Extractions
and Capillary Electrochromatography. J. Chromatogr.
A.

[52] Müller M., Stefanetti F., Krieger U. K. (2023). Oxidation Pathways of Linoleic Acid
Revisited with Electrodynamic Balance–Mass Spectrometry. Environ. Sci.: Atmos..

[53] NIST Chemistry Webbook. Https://Webbook.Nist.Gov/Cgi/Cbook.Cgi?ID=C5392405&Mask=200 (accessed Feb 15, 2025).

[54] Al-Bodour, A. M. R. Creating Advanced Processes Through Utilizing Renewable Eutectics for Gas Capture and Separation, Ph.D. Dissertation, Western Michigan University, Kalamazoo, MI, USA, 2024. https://scholarworks.wmich.edu/dissertations/4076.

[55] Hua J., Björling M., Grahn M., Larsson R., Shi Y. (2019). A Smart Friction
Control Strategy Enabled by CO2 Absorption and Desorption. Sci. Rep..

[56] Gu Y., Hou Y., Ren S., Sun Y., Wu W. (2020). Hydrophobic Functional
Deep Eutectic Solvents Used for Efficient and Reversible Capture of
CO2. ACS Omega.

[57] Kamgar A., Mohsenpour S., Esmaeilzadeh F. (2017). Solubility
Prediction of CO2, CH4,
H2, CO and N2 in Choline Chloride/Urea as a Eutectic Solvent Using
NRTL and COSMO-RS Models. J. Mol. Liq..

[58] Dowson G. R. M., Reed D. G., Bellas J.-M., Charalambous C., Styring P. (2016). Fast and Selective Separation of
Carbon Dioxide from
Dilute Streams by Pressure Swing Adsorption Using Solid Ionic Liquids. Faraday Discuss..

[59] Ramdin M., Amplianitis A., Bazhenov S., Volkov A., Volkov V., Vlugt T. J. H., de Loos T. W. (2014). Solubility of CO2 and CH4 in Ionic
Liquids: Ideal CO2/CH4 Selectivity. Ind. Eng.
Chem. Res..

[60] Lai J. Y., Ngu L. H., Hashim S. S. (2021). A Review
of CO2 Adsorbents Performance
for Different Carbon Capture Technology Processes Conditions. Greenhouse Gases:Sci. Technol..

[61] Lyubartsev A. P., Laaksonen A. M. (2000). DynaMix – a Scalable Portable Parallel MD Simulation
Package for Arbitrary Molecular Mixtures. Comput.
Phys. Commun..

[62] Zoete V., Cuendet M. A., Grosdidier A., Michielin O. (2011). SwissParam:
A Fast Force Field Generation Tool for Small Organic Molecules. J. Comput. Chem..

[63] Ehrman J. N., Lim V. T., Bannan C. C., Thi N., Kyu D. Y., Mobley D. L. (2021). Improving Small Molecule Force Fields by Identifying
and Characterizing Small Molecules with Inconsistent Parameters. J. Comput. Aided Mol. Des..

[64] Jász A. ´., Rák A. ´., Ladjánszki I., Cserey G. (2019). Optimized GPU Implementation of Merck Molecular Force
Field and Universal Force Field. J. Mol. Struct..

[65] Martínez L., Andrade R., Birgin E. G., Martínez J. M. (2009). PACKMOL:
A package for building initial configurations for molecular dynamics
simulations. J. Comput. Chem..

[66] Hoover W. G. (1985). Canonical
Dynamics: Equilibrium Phase-Space Distributions. Phys. Rev. A.

[67] Tuckerman M., Berne B. J., Martyna G. J. (1992). Reversible Multiple Time Scale Molecular
Dynamics. J. Chem. Phys..

[68] Essmann U., Perera L., Berkowitz M. L., Darden T., Lee H., Pedersen L. G. (1995). A Smooth Particle Mesh Ewald Method. J. Chem. Phys..

[69] Humphrey W., Dalke A., Schulten K. (1996). VMD: Visual Molecular Dynamics. J. Mol. Graph..

[70] Brehm M., Kirchner B. (2011). TRAVIS - A Free Analyzer and Visualizer
for Monte Carlo
and Molecular Dynamics Trajectories. J. Chem.
Inf. Model..

