# Supplementary material for: Experimental and Theoretical Insights into CO2 and N2 Capture Using Natural Hydrophobic Deep Eutectic Solvents at High Pressures
Source: ACS Omega. 2025 Jun 4;10(23):24675–96. doi: 10.1021/acsomega.5c01533 (PMC12177657; doi:10.1021/acsomega.5c01533)
Supplement: Supplementary file 1 [file ao5c01533_si_001.pdf]

Electronic Supporting Information (ESI)

Experimental and Theoretical Insights into CO_2 and N_2 Capture Using Natural Hydrophobic Deep Eutectic Solvents at High Pressures

Ahmad Al-Bodour [a, ‡], Noor Alomari [a, ‡], Shan Khai Liew [a], Santiago Aparicio [b*], James Springstead [a*], Mert Atilhan [a*]

[a] *Western Michigan University, Chemical and Paper Engineering Department, 4601 Campus Dr. Floyd Hall, Kalamazoo, MI 49008-5462, United States*

[b] *University of Burgos, Department of Chemistry, Plaza Misael Bañuelos s.n., Burgos 09001, Spain*

[‡]A.A. and N.A. contributed equally to this work

[*]Corresponding Authors: sapar@ubu.es (S.A.), james.springstead@wmich.edu (J.S.) and mert.atilhan@wmich.edu (M.A.)

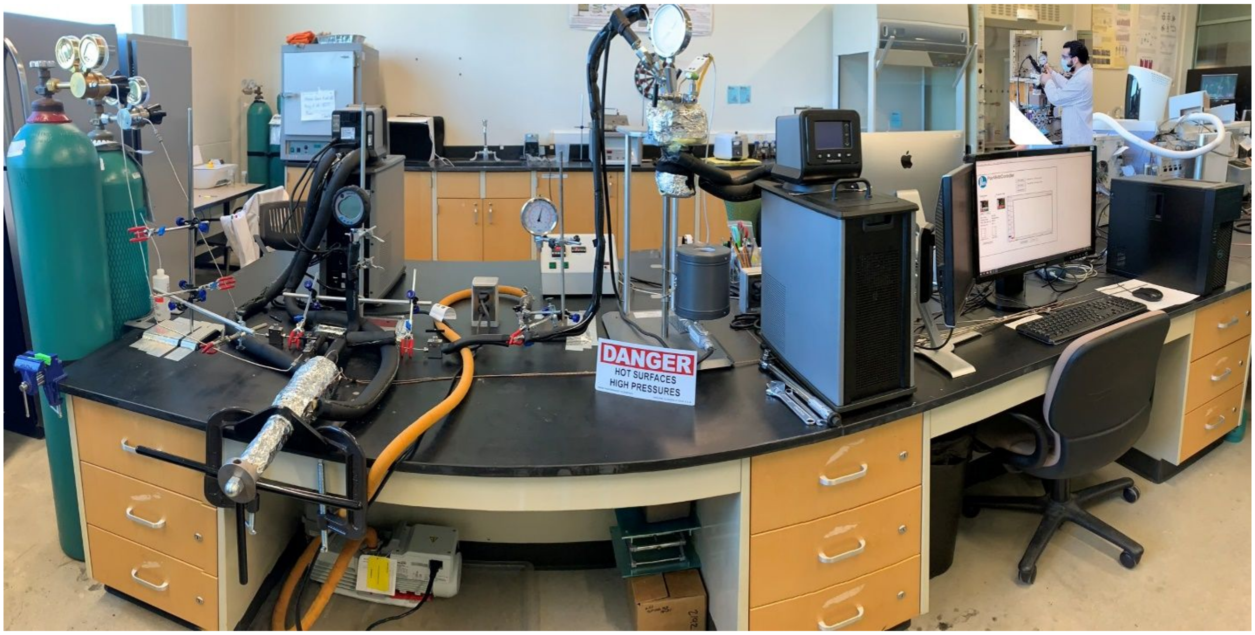

Figure S1. The actual high-pressure gas sorption system in the lab.

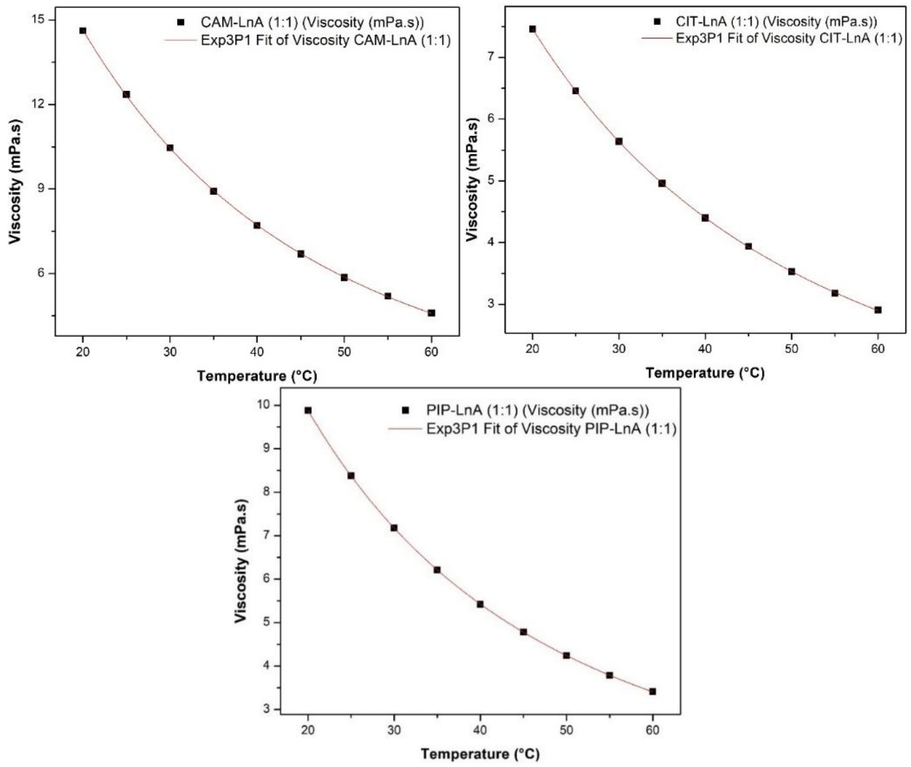

Figure S2. VFT-Model fitting of viscosity data of the studied NADES.

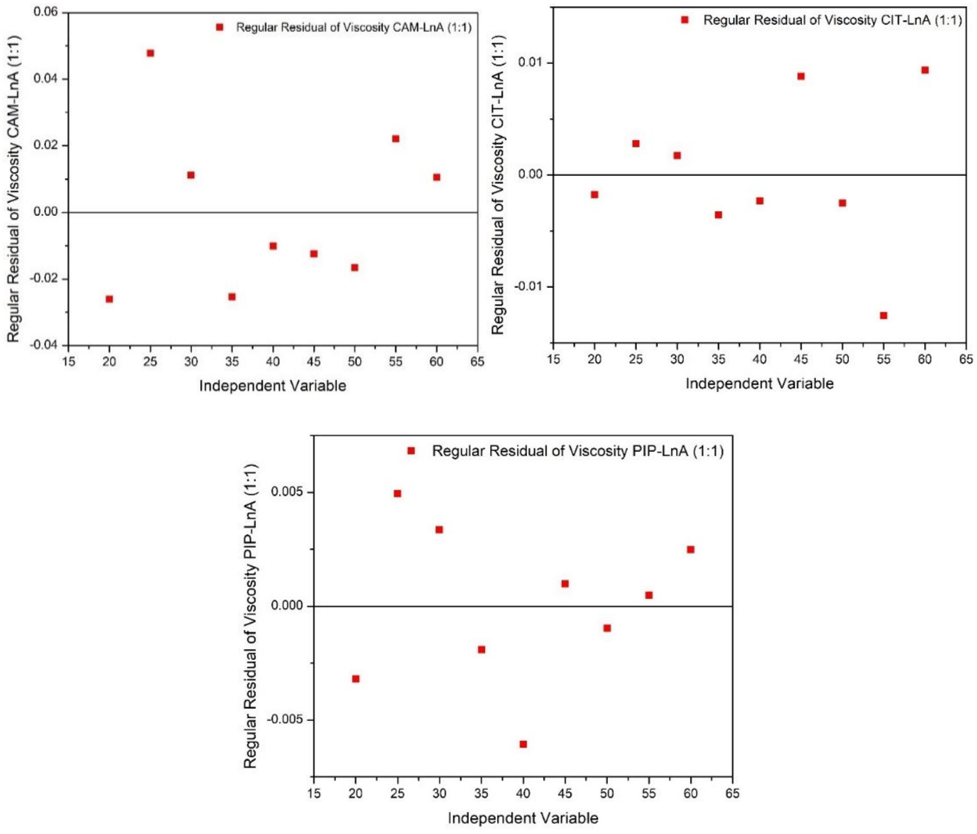

Figure S3. Regular residual of VFT-Model fitting of viscosity of the studied NADES.

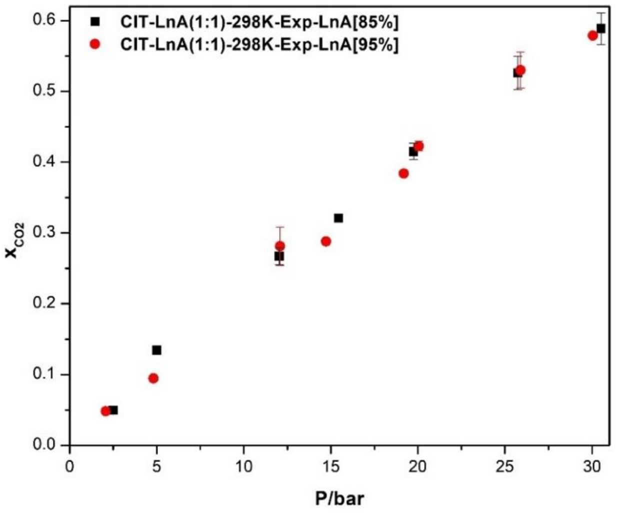

Figure S4. The impact of LnA material purity on the CO_2 absorption capacity.

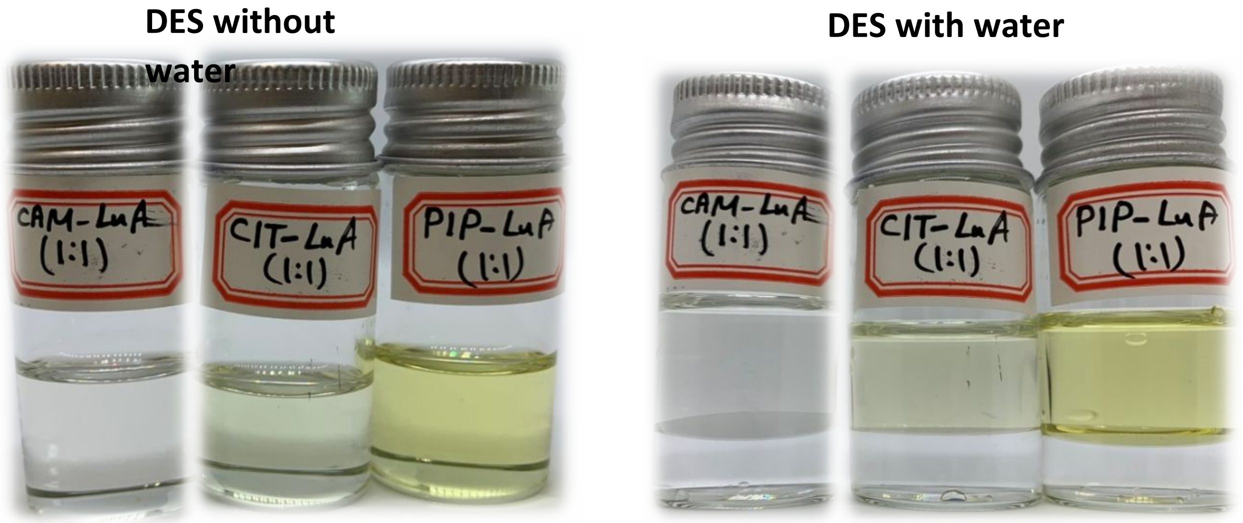

Figure S5. The hydrophobicity of the prepared DES systems.

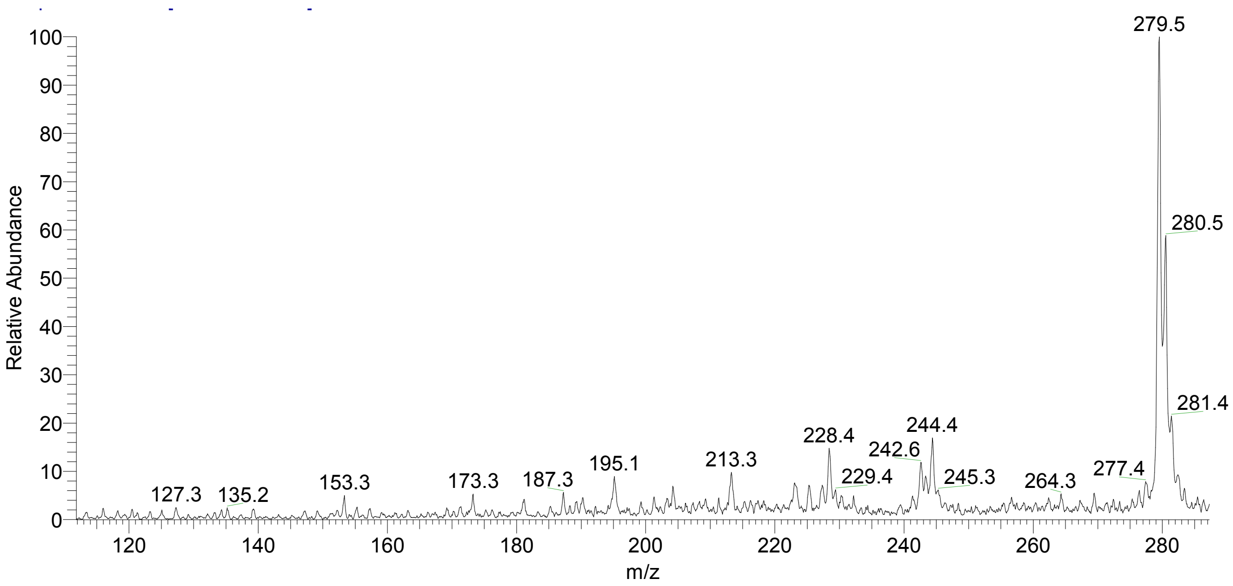

Figure S6. Mass spectrum for Linoleic acid

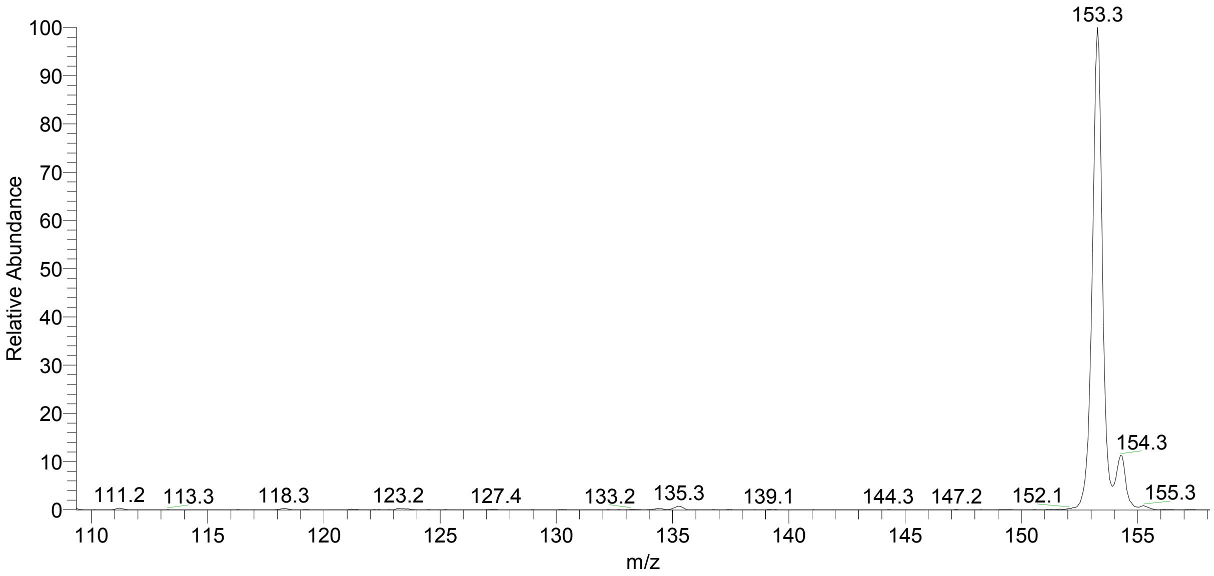

Figure S7. Mass spectrum for Piperitone

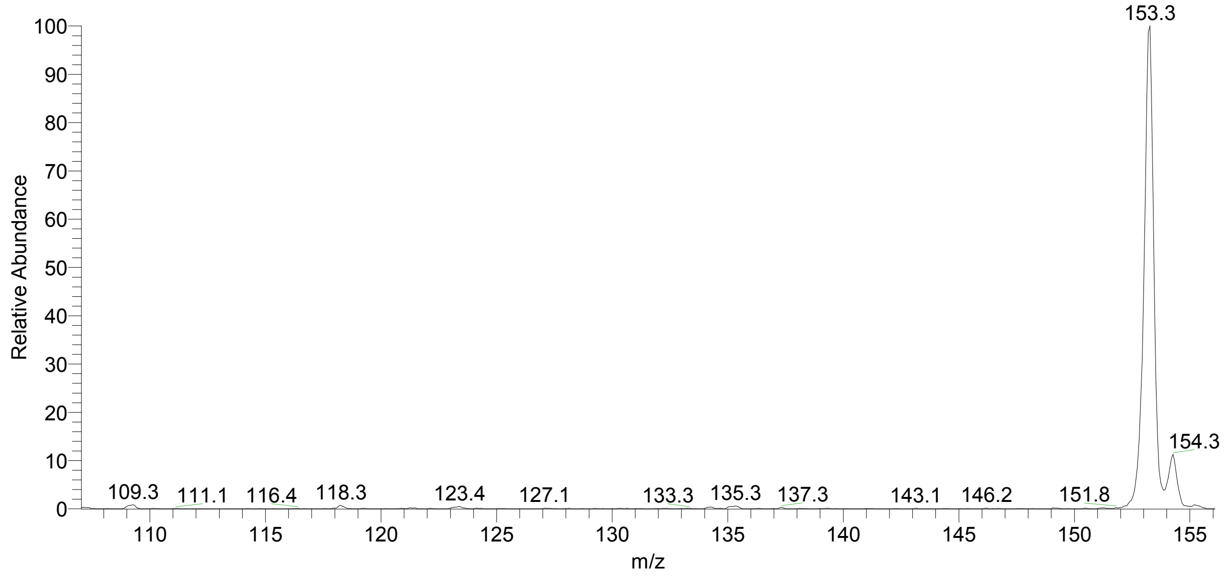

Figure S8. Mass spectrum for Camphor

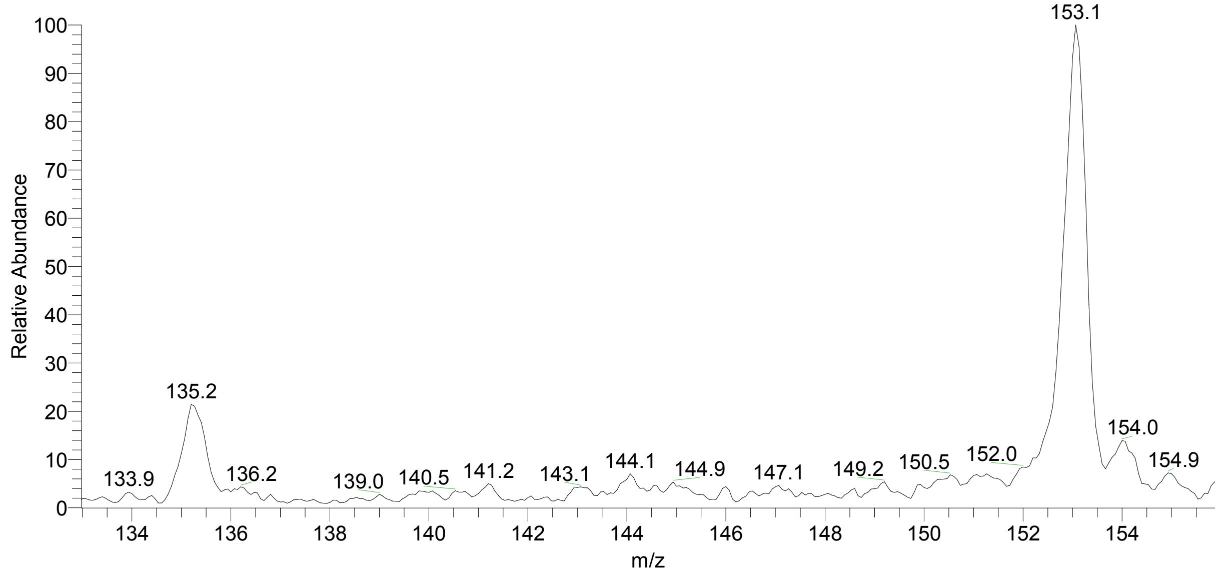

Figure S9. Mass spectrum for Citral

Table S1. Systems considered for NPT molecular dynamics simulations of the studied DES (1:1) and DES (1:1) + CO_2/N_2 mixtures at 293K and 1bar. *X* stands for CO_2/N_2 mole fraction, *N* stands for the number of molecules of each type

x	*N* (HBAs)	*N* (HBD)	*N* (CO_2/N_2)
0	400	400	0
0.05	380	380	20
0.1	360	360	40
0.3	280	280	120
0.5	200	200	200

Table S2. Force field parameterization for compounds studied in MD simulation.

The general form of the applied force field is:

$$E = \sum_{\text{bonds}} k_r (r - r_{eq})^2 + \sum_{\text{angles}} k_\theta \left(\theta - \theta_{eq}\right)^2 + E_{\text{tor}} + \sum_i \sum_j \left\{ 4\varepsilon_{ij} \left[\left(\frac{\sigma_{ij}}{r_{ij}}\right)^{12} - \left(\frac{\sigma_{ij}}{r_{ij}}\right)^{6} \right] + \frac{q_i q_j e^2}{4\pi\varepsilon_0 r_{ij}} \right\}$$

Dihedrals (E_{tor}) were described according to:

$$E_{\text{tor}} = \sum_{\text{torsions}} k_\phi (1 + \cos{(m\phi - \delta)})$$

Improper dihedrals were described according to:

$$E_{impoper} = k_d (\phi - \phi_0)^2$$

CAM

**label**	***q***	**σ_{ii} / Å**	**ε_{ii} / kJ mol^{-1}**	**#**
C1	0.331233	3.550050.292881		
C2	-0.307364	3.550050.292882		
C3	-0.07622	3.550050.292883		
C4	-0.145541	3.550050.292884		

C5	-0.213604	3.550050.292885	
C6	-0.027343	3.550050.292886	
O1	-0.524708	3.153780.636397	
C8	0.795433	3.296320.83680	8
C8	-0.470408	3.029050.502089	
C6	-0.431685	3.029050.5020810	
HZ	0.420009	0.400010.1924611	
HJ	0.164858	2.351970.0920512	
HJ	0.144702	2.351970.0920513	
HJ	0.145263	2.351970.0920514	
HJ	0.195374	2.351970.0920515	

Bonds

Bonds

**Atom Numbers**		**r_{eq} /Å**	**k_r/ kJ mol^{-1} Å^{-2}**
1	6	1.222	3899.333
2	3	1.508	1282.1115
2	4	1.508	1282.1115
2	5	1.482	1366.7245
2	12	1.093	1435.0745
3	6	1.492	1261.639
3	13	1.093	1435.0745
3	14	1.093	1435.0745
4	7	1.482	1366.7245
4	15	1.093	1435.0745
4	16	1.093	1435.0745
5	9	1.482	1366.7245
5	11	1.333	2862.019
6	8	1.468	1374.553
7	8	1.333	2862.019
7	17	1.083	1556.724
8	10	1.482	1366.7245
9	18	1.093	1435.0745
9	19	1.093	1435.0745
9	20	1.093	1435.0745
10	21	1.093	1435.0745
10	22	1.093	1435.0745
10	23	1.093	1435.0745
11	24	1.083	1556.724

11	25		1.083	1556.724

Angles

Atom Numbers			θ_{eq} / deg	k_θ / kJ mol^{-1} rad^{-2}
3	2	4	109.608	512.48
3	2	5	109.445	443.23
3	2	12	110.549	383
4	2	5	109.445	443.23
4	2	12	110.549	383
5	2	12	110.292	380.59
2	3	6	107.517	467.91
2	3	13	110.549	383
2	3	14	110.549	383
6	3	13	108.385	391.44
6	3	14	108.385	391.44
13	3	14	108.836	310.74
2	4	7	109.445	443.23
2	4	15	110.549	383
2	4	16	110.549	383
7	4	15	110.292	380.59
7	4	16	110.292	380.59
15	4	16	108.836	310.74
2	5	9	118.043	452.86
2	5	11	122.141	404.68
9	5	11	122.141	404.68
1	6	3	124.41	564.87
1	6	8	122.623	563.67
3	6	8	116.853	666.04
4	7	8	122.141	404.68
4	7	17	120.108	268.59
8	7	17	121.004	322.18
6	8	7	111.297	328.2
6	8	10	116.104	420.34
7	8	10	122.141	404.68
5	9	18	110.292	380.59
5	9	19	110.292	380.59
5	9	20	110.292	380.59
18	9	19	108.836	310.74
18	9	20	108.836	310.74
19	9	20	108.836	310.74
8	10	21	110.292	380.59
8	10	22	110.292	380.59
8	10	23	110.292	380.59
21	10	22	108.836	310.74
21	10	23	108.836	310.74
22	10	23	108.836	310.74
5	11	24	121.004	322.18

5	11	25	121.004	322.18
24	11	25	119.523	219.8

Dihedrals

**Atom Numbers**				**δ / deg**	**k_{ϕ} / kJ mol^{-1}**	**m**
1	2	3	4	180	14.644	2
1	2	3	13	180	14.644	2
1	6	5	4	180	14.644	2
1	6	5	15	180	14.644	2
1	6	8	9	180	8.368	2
1	6	8	10	180	8.368	2
2	1	6	5	180	14.644	2
2	1	6	8	180	14.644	2
2	1	7	11	180	5.8618	2
2	3	4	5	180	14.644	2
2	3	4	14	180	14.644	2
3	2	1	6	180	14.644	2
3	2	1	7	180	14.644	2
3	4	5	6	180	14.644	2
3	4	5	15	180	14.644	2
4	3	2	12	180	14.644	2
4	5	6	8	180	14.644	2
5	4	3	13	180	14.644	2
5	6	1	7	180	14.644	2
5	6	8	9	180	8.368	2
5	6	8	10	180	8.368	2
6	1	2	12	180	14.644	2
6	1	7	11	180	5.8618	2
6	5	4	14	180	14.644	2
7	1	2	12	180	14.644	2

7	1	6	8	180	14.644	2
8	6	5	15	180	14.644	2
12	2	3	13	180	14.644	2
13	3	4	14	180	14.644	2
14	4	5	15	180	14.644	2

Improper

**Atom Numbers**				**ϕ_0 / deg**	**k_ϕ / kJ mol^{-1}**
1	2	6	7	0	28.9031
2	3	1	12	0	9.0291
3	4	2	13	0	9.0291
4	5	3	14	0	9.0291
6	5	1	8	0	27.6981
8	9	6	10	0	-3.0125
5	6	4	15	0	9.0291

CIT

#

**label**	***q***	**σ_{ii} / Å**	**ε_{ii} / kJ mol^{-1}**	**#**
CB	-0.124378	3.55005	0.29288	
CJ	-0.359592	3.550050.29288		
CJ	0.477819	3.550050.29288		
CJ	-0.228031	3.550050.29288		
CJ	-0.210277	3.550050.29288		
CA	0.036241	3.550050.29288		
O1	0.754679	3.296320.8368		
H4	-0.452791	3.029050.50208		
H5	-0.464483	3.029050.50208		
HZ	-0.603374	3.153780.636386		

HJ 0.181316 0.400010.192464

HJ 0.178099 2.351970.092048

HJ 0.188272.351970.092048

HJ 0.191774 2.351970.092048

HJ 0.434728 2.351970.092048

Bonds

**Atom Numbers**			r_{eq} **/Å**	k_r**/ kJ mol**$^{-1}$ **Å**$^{-2}$
#	N1	N2	R-eqv	Force Type
0	1	2	1.374	1678.0685
0	2	3	1.374	1678.0685
0	3	4	1.374	1678.0685
0	4	5	1.374	1678.0685
0	5	6	1.374	1678.0685
0	6	1	1.374	1678.0685
0	5	7	1.374	1678.0685
0	7	9	1.431	1416.7065
0	7	8	1.233	2836.4255
0	2	10	1.233	2836.4255
0	10	11	1.376	1690.4155
0	1	12	0.973	2360.3745
0	3	15	1.084	1597.673
0	4	14	1.084	1597.673
0	6	13	1.084	1597.673

Angles

**Atom Numbers**			θ_{eq} **/ deg**	k_θ **/ kJ mol**$^{-1}$ **rad**$^{-2}$
	2	1	6	119.977402.88

2	1	12	120.571339.05
6	1	12	120.571339.05
1	2	3	119.977402.88
1	2	10	116.495582.94
3	2	10	116.495582.94
2	3	4	119.977402.88
2	3	15	120.571339.05
4	3	15	120.571339.05
3	4	5	119.977402.88
3	4	14	120.571339.05
5	4	14	120.571339.05
4	5	6	119.977402.88
4	5	7	112.337670.86
6	5	7	112.337670.86
1	6	5	119.977402.88
1	6	13	120.571339.05
5	6	13	120.571339.05
5	7	8	117.857781.66
5	7	9	117.857781.66
8	7	9	128.036883.44
2	10	11	105.409437.2

Dihedrals

**Atom Numbers**				**δ / deg**	**k_ϕ / kJ mol^{-1}**	**m**
1	2	3	4	180	14.644	2
1	2	3	15	180	14.644	2
1	2	10	11	180	5.8618	2
1	6	5	4	180	14.644	2
1	6	5	7	180	14.644	2
2	1	6	5	180	14.644	2

2	1	6	13	180	14.644	2
2	3	4	5	180	14.644	2
2	3	4	14	180	14.644	2
3	2	1	6	180	14.644	2
3	2	1	12	180	14.644	2
3	2	10	11	180	5.8618	2
3	4	5	6	180	14.644	2
3	4	5	7	180	14.644	2
4	3	2	10	180	14.644	2
4	5	6	13	180	14.644	2
4	5	7	8	180	3.7656	2
4	5	7	9	180	3.7656	2
5	4	3	15	180	14.644	2
5	6	1	12	180	14.644	2
6	1	2	10	180	14.644	2
6	5	4	14	180	14.644	2
6	5	7	8	180	3.7656	2
6	5	7	9	180	3.7656	2
7	5	4	14	180	14.644	2
7	5	6	13	180	14.644	2
10	2	1	12	180	14.644	2
10	2	3	15	180	14.644	2
12	1	6	13	180	14.644	2
14	4	3	15	180	14.644	2

Improper

**Atom Numbers**				**ϕ_0 / deg**	**k_ϕ / kJ mol^{-1}**
1	2	6	12	0	9.0291
2	3	1	10	0	28.9031

3	4	2	15	0	9.0291
4	5	3	14	0	9.0291
5	6	4	7	0	21.079
7	9	5	8	0	90.3326
6	5	1	13	0	9.0291

PIP

q	σ_{ii} / Å	ε_{ii} / kJ mol^{-1}	#
-0.727613	3.15378	0.636386	1
-0.08927	3.87541	0.23012	2
0.756874	3.87541	0.23012	3
1.040726	3.87541	0.23012	4
-0.085486	3.87541	0.23012	5
-0.087383	3.87541	0.23012	6
-0.182317	3.87541	0.23012	7
-0.180233	3.87541	0.23012	8
-0.630259	3.87541	0.23012	9
-0.703498	3.87541	0.23012	10
-0.708447	3.87541	0.23012	11
-0.005404	2.35197	0.092048	12
0.018789	2.35197	0.092048	13
0.043211	2.35197	0.092048	14
0.043968	2.35197	0.092048	15
0.018971	2.35197	0.092048	16
0.030833	2.35197	0.092048	17
0.036502	2.35197	0.092048	18
0.035374	2.35197	0.092048	19
0.030653	2.35197	0.092048	20
0.113756	2.35197	0.092048	21
0.157233	2.35197	0.092048	22
0.157314	2.35197	0.092048	23
0.128536	2.35197	0.092048	24
0.160146	2.35197	0.092048	25
0.167092	2.35197	0.092048	26
0.129617	2.35197	0.092048	27
0.168862	2.35197	0.092048	28
0.161454	2.35197	0.092048	29

Bonds

**Atom Numbers**		**r_{eq} /Å**	**k_r/ kJ mol^{-1} Å^{-2}**
1	18	1.355	1746.7195
1	52	0.981	2229.093
2	18	1.222	3899.333
3	4	1.508	1282.1115
3	5	1.508	1282.1115
3	21	1.093	1435.0745
3	22	1.093	1435.0745
4	6	1.508	1282.1115
4	23	1.093	1435.0745
4	24	1.093	1435.0745
5	7	1.508	1282.1115
5	25	1.093	1435.0745
5	26	1.093	1435.0745
6	8	1.508	1282.1115
6	27	1.093	1435.0745
6	28	1.093	1435.0745
7	9	1.508	1282.1115
7	29	1.093	1435.0745
7	30	1.093	1435.0745
8	12	1.482	1366.7245
8	31	1.093	1435.0745
8	32	1.093	1435.0745
9	18	1.492	1261.639
9	33	1.093	1435.0745
9	34	1.093	1435.0745

10	11	1.508	1282.1115
10	13	1.508	1282.1115
10	35	1.093	1435.0745
10	36	1.093	1435.0745
11	14	1.508	1282.1115
11	37	1.093	1435.0745
11	38	1.093	1435.0745
12	15	1.333	2862.019
12	39	1.083	1556.724
13	17	1.482	1366.7245
13	40	1.093	1435.0745
13	41	1.093	1435.0745
14	20	1.508	1282.1115
14	42	1.093	1435.0745
14	43	1.093	1435.0745
15	16	1.482	1366.7245
15	44	1.083	1556.724
16	19	1.482	1366.7245
16	45	1.093	1435.0745
16	46	1.093	1435.0745
17	19	1.333	2862.019
17	47	1.083	1556.724
19	48	1.083	1556.724
20	49	1.093	1435.0745
20	50	1.093	1435.0745
20	51	1.093	1435.0745

Angles

**Atom Numbers**			**θ_{eq} / deg**	**k_θ / kJ mol^{-1} rad^{-2}**
18	1	52	111.948	351.09
4	3	5	109.608	512.48
4	3	21	110.549	383
4	3	22	110.549	383
5	3	21	110.549	383
5	3	22	110.549	383
21	3	22	108.836	310.74
3	4	6	109.608	512.48
3	4	23	110.549	383
3	4	24	110.549	383
6	4	23	110.549	383
6	4	24	110.549	383
23	4	24	108.836	310.74
3	5	7	109.608	512.48
3	5	25	110.549	383
3	5	26	110.549	383
7	5	25	110.549	383
7	5	26	110.549	383
25	5	26	108.836	310.74
4	6	8	109.608	512.48
4	6	27	110.549	383
4	6	28	110.549	383
8	6	27	110.549	383
8	6	28	110.549	383
27	6	28	108.836	310.74

5	7	9	109.608	512.48
5	7	29	110.549	383
5	7	30	110.549	383
9	7	29	110.549	383
9	7	30	110.549	383
29	7	30	108.836	310.74
6	8	12	109.445	443.23
6	8	31	110.549	383
6	8	32	110.549	383
12	8	31	110.292	380.59
12	8	32	110.292	380.59
31	8	32	108.836	310.74
7	9	18	107.517	467.91
7	9	33	110.549	383
7	9	34	110.549	383
18	9	33	108.385	391.44
18	9	34	108.385	391.44
33	9	34	108.836	310.74
11	10	13	109.608	512.48
11	10	35	110.549	383
11	10	36	110.549	383
13	10	35	110.549	383
13	10	36	110.549	383
35	10	36	108.836	310.74
10	11	14	109.608	512.48
10	11	37	110.549	383
10	11	38	110.549	383

14	11	37	110.549	383
14	11	38	110.549	383
37	11	38	108.836	310.74
8	12	15	122.141	404.68
8	12	39	120.108	268.59
15	12	39	121.004	322.18
10	13	17	109.445	443.23
10	13	40	110.549	383
10	13	41	110.549	383
17	13	40	110.292	380.59
17	13	41	110.292	380.59
40	13	41	108.836	310.74
11	14	20	109.608	512.48
11	14	42	110.549	383
11	14	43	110.549	383
20	14	42	110.549	383
20	14	43	110.549	383
42	14	43	108.836	310.74
12	15	16	122.141	404.68
12	15	44	121.004	322.18
16	15	44	120.108	268.59
15	16	19	111.453	670.26
15	16	45	110.292	380.59
15	16	46	110.292	380.59
19	16	45	110.292	380.59
19	16	46	110.292	380.59
45	16	46	108.836	310.74

13	17	19	122.141	404.68
13	17	47	120.108	268.59
19	17	47	121.004	322.18
1	18	2	124.425	695.55
1	18	9	109.716	628.1
2	18	9	124.41 564.87	
16	19	17	122.141	404.68
16	19	48	120.108	268.59
17	19	48	121.004	322.18
14	20	49	110.549	383
14	20	50	110.549	383
14	20	51	110.549	383
49	20	50	108.836	310.74
49	20	51	108.836	310.74
50	20	51	108.836	310.74

Dihedrals

**Atom Numbers**				**δ / deg**	**k_ϕ / kJ mol^{-1}**	**m**
1	18	9	7	0	-0.2469	1
1	18	9	7	180	-0.6987	2
1	18	9	7	0	0.4226	3
1	18	9	33	180	-1.3054	2
1	18	9	33	0	0.6904	3
1	18	9	34	180	-1.3054	2
1	18	9	34	0	0.6904	3
2	18	1	52	0	3.4769	1
2	18	1	52	180	12.87	2

2	18	1	52	0	-0.1213	3
2	18	9	7	0	1.7238	1
2	18	9	7	180	0.2929	2
2	18	9	7	0	0.682	3
2	18	9	33	0	1.3807	1
2	18	9	33	180	-2.9455	2
2	18	9	33	0	0.6443	3
2	18	9	34	0	1.3807	1
2	18	9	34	180	-2.9455	2
2	18	9	34	0	0.6443	3
3	4	6	8	0	0.2134	1
3	4	6	8	180	1.4267	2
3	4	6	8	0	0.6945	3
3	4	6	27	0	1.3389	1
3	4	6	27	180	-1.318	2
3	4	6	27	0	0.5523	3
3	4	6	28	0	1.3389	1
3	4	6	28	180	-1.318	2
3	4	6	28	0	0.5523	3
3	5	7	9	0	0.2134	1
3	5	7	9	180	1.4267	2
3	5	7	9	0	0.6945	3
3	5	7	29	0	1.3389	1
3	5	7	29	180	-1.318	2
3	5	7	29	0	0.5523	3
3	5	7	30	0	1.3389	1
3	5	7	30	180	-1.318	2

3	5	7	30	0	0.5523	3
4	3	5	7	0	0.2134	1
4	3	5	7	180	1.4267	2
4	3	5	7	0	0.6945	3
4	3	5	25	0	1.3389	1
4	3	5	25	180	-1.318	2
4	3	5	25	0	0.5523	3
4	3	5	26	0	1.3389	1
4	3	5	26	180	-1.318	2
4	3	5	26	0	0.5523	3
4	6	8	12	0	-0.615	1
4	6	8	12	180	0.9163	2
4	6	8	12	0	1.2217	3
4	6	8	31	0	1.3389	1
4	6	8	31	180	-1.318	2
4	6	8	31	0	0.5523	3
4	6	8	32	0	1.3389	1
4	6	8	32	180	-1.318	2
4	6	8	32	0	0.5523	3
5	3	4	6	0	0.2134	1
5	3	4	6	180	1.4267	2
5	3	4	6	0	0.6945	3
5	3	4	23	0	1.3389	1
5	3	4	23	180	-1.318	2
5	3	4	23	0	0.5523	3
5	3	4	24	0	1.3389	1
5	3	4	24	180	-1.318	2

5	3	4	24	0	0.5523	3
5	7	9	18	0	0.1381	1
5	7	9	18	180	-0.3264	2
5	7	9	18	0	0.2971	3
5	7	9	33	0	1.3389	1
5	7	9	33	180	-1.318	2
5	7	9	33	0	0.5523	3
5	7	9	34	0	1.3389	1
5	7	9	34	180	-1.318	2
5	7	9	34	0	0.5523	3
6	4	3	21	0	1.3389	1
6	4	3	21	180	-1.318	2
6	4	3	21	0	0.5523	3
6	4	3	22	0	1.3389	1
6	4	3	22	180	-1.318	2
6	4	3	22	0	0.5523	3
6	8	12	15	0	-1.0334	1
6	8	12	15	180	0.5732	2
6	8	12	15	0	-1.318	3
6	8	12	39	0	0.1548	1
6	8	12	39	0	0.7489	3
7	5	3	21	0	1.3389	1
7	5	3	21	180	-1.318	2
7	5	3	21	0	0.5523	3
7	5	3	22	0	1.3389	1
7	5	3	22	180	-1.318	2
7	5	3	22	0	0.5523	3

8	6	4	23	0	1.3389	1
8	6	4	23	180	-1.318	2
8	6	4	23	0	0.5523	3
8	6	4	24	0	1.3389	1
8	6	4	24	180	-1.318	2
8	6	4	24	0	0.5523	3
8	12	15	16	0	-0.8452	1
8	12	15	16	180	25.104	2
8	12	15	44	180	25.104	2
9	7	5	25	0	1.3389	1
9	7	5	25	180	-1.318	2
9	7	5	25	0	0.5523	3
9	7	5	26	0	1.3389	1
9	7	5	26	180	-1.318	2
9	7	5	26	0	0.5523	3
9	18	1	52	0	-2.4393	1
9	18	1	52	180	10.6232	2
9	18	1	52	0	-1.1422	3
10	11	14	20	0	0.2134	1
10	11	14	20	180	1.4267	2
10	11	14	20	0	0.6945	3
10	11	14	42	0	1.3389	1
10	11	14	42	180	-1.318	2
10	11	14	42	0	0.5523	3
10	11	14	43	0	1.3389	1
10	11	14	43	180	-1.318	2
10	11	14	43	0	0.5523	3

10	13	17	19	0	-1.0334	1
10	13	17	19	180	0.5732	2
10	13	17	19	0	-1.318	3
10	13	17	47	0	0.1548	1
10	13	17	47	0	0.7489	3
11	10	13	17	0	-0.615	1
11	10	13	17	180	0.9163	2
11	10	13	17	0	1.2217	3
11	10	13	40	0	1.3389	1
11	10	13	40	180	-1.318	2
11	10	13	40	0	0.5523	3
11	10	13	41	0	1.3389	1
11	10	13	41	180	-1.318	2
11	10	13	41	0	0.5523	3
11	14	20	49	0	1.3389	1
11	14	20	49	180	-1.318	2
11	14	20	49	0	0.5523	3
11	14	20	50	0	1.3389	1
11	14	20	50	180	-1.318	2
11	14	20	50	0	0.5523	3
11	14	20	51	0	1.3389	1
11	14	20	51	180	-1.318	2
11	14	20	51	0	0.5523	3
12	8	6	27	0	0.6736	1
12	8	6	27	180	-0.8577	2
12	8	6	27	0	0.3012	3
12	8	6	28	0	0.6736	1

12	8	6	28	180	-0.8577	2
12	8	6	28	0	0.3012	3
12	15	16	19	0	-0.6109	1
12	15	16	19	180	0.2427	2
12	15	16	19	0	-1.0627	3
12	15	16	45	0	1.0502	1
12	15	16	45	180	-0.8577	2
12	15	16	45	0	-1.1213	3
12	15	16	46	0	1.0502	1
12	15	16	46	180	-0.8577	2
12	15	16	46	0	-1.1213	3
13	10	11	14	0	0.2134	1
13	10	11	14	180	1.4267	2
13	10	11	14	0	0.6945	3
13	10	11	37	0	1.3389	1
13	10	11	37	180	-1.318	2
13	10	11	37	0	0.5523	3
13	10	11	38	0	1.3389	1
13	10	11	38	180	-1.318	2
13	10	11	38	0	0.5523	3
13	17	19	16	0	-0.8452	1
13	17	19	16	180	25.104	2
13	17	19	48	180	25.104	2
14	11	10	35	0	1.3389	1
14	11	10	35	180	-1.318	2
14	11	10	35	0	0.5523	3
14	11	10	36	0	1.3389	1

14	11	10	36	180	-1.318	2
14	11	10	36	0	0.5523	3
15	12	8	31	0	1.0502	1
15	12	8	31	180	-0.8577	2
15	12	8	31	0	-1.1213	3
15	12	8	32	0	1.0502	1
15	12	8	32	180	-0.8577	2
15	12	8	32	0	-1.1213	3
15	16	19	17	0	-0.6109	1
15	16	19	17	180	0.2427	2
15	16	19	17	0	-1.0627	3
15	16	19	48	0	0.6276	1
15	16	19	48	180	0.2176	2
15	16	19	48	0	1.0627	3
16	15	12	39	180	25.104	2
16	19	17	47	180	25.104	2
17	13	10	35	0	0.6736	1
17	13	10	35	180	-0.8577	2
17	13	10	35	0	0.3012	3
17	13	10	36	0	0.6736	1
17	13	10	36	180	-0.8577	2
17	13	10	36	0	0.3012	3
17	19	16	45	0	1.0502	1
17	19	16	45	180	-0.8577	2
17	19	16	45	0	-1.1213	3
17	19	16	46	0	1.0502	1
17	19	16	46	180	-0.8577	2

17	19	16	46	0	-1.1213	3
18	9	7	29	0	-0.5356	1
18	9	7	29	180	0.1213	2
18	9	7	30	0	-0.5356	1
18	9	7	30	180	0.1213	2
19	16	15	44	0	0.6276	1
19	16	15	44	180	0.2176	2
19	16	15	44	0	1.0627	3
19	17	13	40	0	1.0502	1
19	17	13	40	180	-0.8577	2
19	17	13	40	0	-1.1213	3
19	17	13	41	0	1.0502	1
19	17	13	41	180	-0.8577	2
19	17	13	41	0	-1.1213	3
20	14	11	37	0	1.3389	1
20	14	11	37	180	-1.318	2
20	14	11	37	0	0.5523	3
20	14	11	38	0	1.3389	1
20	14	11	38	180	-1.318	2
20	14	11	38	0	0.5523	3
21	3	4	23	0	0.5941	1
21	3	4	23	180	-2.8995	2
21	3	4	23	0	0.6569	3
21	3	4	24	0	0.5941	1
21	3	4	24	180	-2.8995	2
21	3	4	24	0	0.6569	3

LnA

label	q	σ_{ii} / Å	ε_{ii} / kJ mol^{-1}	#
O2	-0.676989	3.153780.636386		1
O3	-0.597888	3.029050.502082		
C8	0.109653.875410.230123			
C8	-0.093895	3.875410.230124		
C8	-0.002634	3.875410.230125		
C8	0.089646	3.875410.230126		
C8	-0.045956	3.875410.230127		
C8	0.263279	3.875410.230128		
C7	-0.173077	3.875410.230129		
C8	0.020909	3.875410.2301210		
C8	-0.123995	3.875410.2301211		
C8	-0.406697	3.723960.284512		12
C8	0.303591	3.875410.2301213		
C8	0.310303	3.875410.2301214		
C8	-0.183212	3.723960.284512		15
C8	0.172943.875410.2301216			
C8	-0.387605	3.723960.284512		17
C6	0.793424	3.563590.4602418		
C8	-0.229031	3.723960.284512		19
C8	-0.345572	3.875410.2301220		
H6	-0.024251	2.351970.092048		21
H6	-0.024187	2.351970.092048		22
H6	0.000307	2.351970.092048		23
H6	-0.000504	2.351970.092048		24
H6	0.003622.351970.092048		25	
H6	0.003822.351970.092048		26	

H6	-0.025439	2.351970.092048	27
H6	-0.005991	2.351970.092048	28
H6	0.037721	2.351970.092048	29
H6	0.034969	2.351970.092048	30
H6	-0.034944	2.351970.092048	31
H6	-0.030262	2.351970.092048	32
H5	0.077441	2.351970.092048	33
H5	0.076223	2.351970.092048	34
H6	0.003872.351970.092048		35
H6	-0.016512	2.351970.092048	36
H6	0.012234	2.351970.092048	37
H6	0.010872.351970.092048		38
H6	0.153435	2.351970.092048	39
H6	-0.036413	2.351970.092048	40
H6	-0.040667	2.351970.092048	41
H6	-0.055412	2.351970.092048	42
H6	-0.058467	2.351970.092048	43
H6	0.122669	2.351970.092048	44
H6	0.058227	2.351970.092048	45
H6	0.007404	2.351970.092048	46
H6	0.153045	2.351970.092048	47
H6	0.134795	2.351970.092048	48
H6	0.071281	2.351970.092048	49
H6	0.072896	2.351970.092048	50
H6	0.076865	2.351970.092048	51
H4	0.444166	0.400010.192464	52

Bonds

Atom Numbers **r_{eq} /Å** **k_r/ kJ mol^{-1} $Å^{-2}$**

1	18	1.355	1746.7195
1	52	0.981	2229.093
2	18	1.222	3899.333
3	4	1.508	1282.1115
3	5	1.508	1282.1115
3	21	1.093	1435.0745
3	22	1.093	1435.0745
4	6	1.508	1282.1115
4	23	1.093	1435.0745
4	24	1.093	1435.0745
5	7	1.508	1282.1115
5	25	1.093	1435.0745
5	26	1.093	1435.0745
6	8	1.508	1282.1115
6	27	1.093	1435.0745
6	28	1.093	1435.0745
7	9	1.508	1282.1115
7	29	1.093	1435.0745
7	30	1.093	1435.0745
8	12	1.482	1366.7245
8	31	1.093	1435.0745
8	32	1.093	1435.0745
9	18	1.492	1261.639
9	33	1.093	1435.0745
9	34	1.093	1435.0745
10	11	1.508	1282.1115
10	13	1.508	1282.1115
10	35	1.093	1435.0745
10	36	1.093	1435.0745

11	14	1.508	1282.1115
11	37	1.093	1435.0745
11	38	1.093	1435.0745
12	15	1.333	2862.019
12	39	1.083	1556.724
13	17	1.482	1366.7245
13	40	1.093	1435.0745
13	41	1.093	1435.0745
14	20	1.508	1282.1115
14	42	1.093	1435.0745
14	43	1.093	1435.0745
15	16	1.482	1366.7245
15	44	1.083	1556.724
16	19	1.482	1366.7245
16	45	1.093	1435.0745
16	46	1.093	1435.0745
17	19	1.333	2862.019
17	47	1.083	1556.724
19	48	1.083	1556.724
20	49	1.093	1435.0745
20	50	1.093	1435.0745
20	51	1.093	1435.0745

Angles

**Atom Numbers**			**θ_{eq} / deg**	**k_θ / kJ mol^{-1} rad^{-2}**
18	1	52	111.948	351.09
4	3	5	109.608	512.48
4	3	21	110.549	383
4	3	22	110.549	383

5	3	21	110.549	383
5	3	22	110.549	383
21	3	22	108.836	310.74
3	4	6	109.608	512.48
3	4	23	110.549	383
3	4	24	110.549	383
6	4	23	110.549	383
6	4	24	110.549	383
23	4	24	108.836	310.74
3	5	7	109.608	512.48
3	5	25	110.549	383
3	5	26	110.549	383
7	5	25	110.549	383
7	5	26	110.549	383
25	5	26	108.836	310.74
4	6	8	109.608	512.48
4	6	27	110.549	383
4	6	28	110.549	383
8	6	27	110.549	383
8	6	28	110.549	383
27	6	28	108.836	310.74
5	7	9	109.608	512.48
5	7	29	110.549	383
5	7	30	110.549	383
9	7	29	110.549	383
9	7	30	110.549	383
29	7	30	108.836	310.74
6	8	12	109.445	443.23
6	8	31	110.549	383

6	8	32	110.549	383
12	8	31	110.292	380.59
12	8	32	110.292	380.59
31	8	32	108.836	310.74
7	9	18	107.517	467.91
7	9	33	110.549	383
7	9	34	110.549	383
18	9	33	108.385	391.44
18	9	34	108.385	391.44
33	9	34	108.836	310.74
11	10	13	109.608	512.48
11	10	35	110.549	383
11	10	36	110.549	383
13	10	35	110.549	383
13	10	36	110.549	383
35	10	36	108.836	310.74
10	11	14	109.608	512.48
10	11	37	110.549	383
10	11	38	110.549	383
14	11	37	110.549	383
14	11	38	110.549	383
37	11	38	108.836	310.74
8	12	15	122.141	404.68
8	12	39	120.108	268.59
15	12	39	121.004	322.18
10	13	17	109.445	443.23
10	13	40	110.549	383
10	13	41	110.549	383
17	13	40	110.292	380.59

17	13	41	110.292	380.59
40	13	41	108.836	310.74
11	14	20	109.608	512.48
11	14	42	110.549	383
11	14	43	110.549	383
20	14	42	110.549	383
20	14	43	110.549	383
42	14	43	108.836	310.74
12	15	16	122.141	404.68
12	15	44	121.004	322.18
16	15	44	120.108	268.59
15	16	19	111.453	670.26
15	16	45	110.292	380.59
15	16	46	110.292	380.59
19	16	45	110.292	380.59
19	16	46	110.292	380.59
45	16	46	108.836	310.74
13	17	19	122.141	404.68
13	17	47	120.108	268.59
19	17	47	121.004	322.18
1	18	2	124.425	695.55
1	18	9	109.716	628.1
2	18	9	124.41 564.87	
16	19	17	122.141	404.68
16	19	48	120.108	268.59
17	19	48	121.004	322.18
14	20	49	110.549	383
14	20	50	110.549	383
14	20	51	110.549	383

49	20	50	108.836	310.74
49	20	51	108.836	310.74
50	20	51	108.836	310.74

Dihedrals

**Atom Numbers**				**δ / deg**	**k_ϕ / kJ mol^{-1}**	**m**
1	18	9	7	0	-0.2469	1
1	18	9	7	180	-0.6987	2
1	18	9	7	0	0.4226	3
1	18	9	33	180	-1.3054	2
1	18	9	33	0	0.6904	3
1	18	9	34	180	-1.3054	2
1	18	9	34	0	0.6904	3
2	18	1	52	0	3.4769	1
2	18	1	52	180	12.87	2
2	18	1	52	0	-0.1213	3
2	18	9	7	0	1.7238	1
2	18	9	7	180	0.2929	2
2	18	9	7	0	0.682	3
2	18	9	33	0	1.3807	1
2	18	9	33	180	-2.9455	2
2	18	9	33	0	0.6443	3
2	18	9	34	0	1.3807	1
2	18	9	34	180	-2.9455	2
2	18	9	34	0	0.6443	3
3	4	6	8	0	0.2134	1
3	4	6	8	180	1.4267	2
3	4	6	8	0	0.6945	3
3	4	6	27	0	1.3389	1

3	4	6	27	180	-1.318	2
3	4	6	27	0	0.5523	3
3	4	6	28	0	1.3389	1
3	4	6	28	180	-1.318	2
3	4	6	28	0	0.5523	3
3	5	7	9	0	0.2134	1
3	5	7	9	180	1.4267	2
3	5	7	9	0	0.6945	3
3	5	7	29	0	1.3389	1
3	5	7	29	180	-1.318	2
3	5	7	29	0	0.5523	3
3	5	7	30	0	1.3389	1
3	5	7	30	180	-1.318	2
3	5	7	30	0	0.5523	3
4	3	5	7	0	0.2134	1
4	3	5	7	180	1.4267	2
4	3	5	7	0	0.6945	3
4	3	5	25	0	1.3389	1
4	3	5	25	180	-1.318	2
4	3	5	25	0	0.5523	3
4	3	5	26	0	1.3389	1
4	3	5	26	180	-1.318	2
4	3	5	26	0	0.5523	3
4	6	8	12	0	-0.615	1
4	6	8	12	180	0.9163	2
4	6	8	12	0	1.2217	3
4	6	8	31	0	1.3389	1
4	6	8	31	180	-1.318	2
4	6	8	31	0	0.5523	3

4	6	8	32	0	1.3389	1
4	6	8	32	180	-1.318	2
4	6	8	32	0	0.5523	3
5	3	4	6	0	0.2134	1
5	3	4	6	180	1.4267	2
5	3	4	6	0	0.6945	3
5	3	4	23	0	1.3389	1
5	3	4	23	180	-1.318	2
5	3	4	23	0	0.5523	3
5	3	4	24	0	1.3389	1
5	3	4	24	180	-1.318	2
5	3	4	24	0	0.5523	3
5	7	9	18	0	0.1381	1
5	7	9	18	180	-0.3264	2
5	7	9	18	0	0.2971	3
5	7	9	33	0	1.3389	1
5	7	9	33	180	-1.318	2
5	7	9	33	0	0.5523	3
5	7	9	34	0	1.3389	1
5	7	9	34	180	-1.318	2
5	7	9	34	0	0.5523	3
6	4	3	21	0	1.3389	1
6	4	3	21	180	-1.318	2
6	4	3	21	0	0.5523	3
6	4	3	22	0	1.3389	1
6	4	3	22	180	-1.318	2
6	4	3	22	0	0.5523	3
6	8	12	15	0	-1.0334	1
6	8	12	15	180	0.5732	2

6	8	12	15	0	-1.318	3
6	8	12	39	0	0.1548	1
6	8	12	39	0	0.7489	3
7	5	3	21	0	1.3389	1
7	5	3	21	180	-1.318	2
7	5	3	21	0	0.5523	3
7	5	3	22	0	1.3389	1
7	5	3	22	180	-1.318	2
7	5	3	22	0	0.5523	3
8	6	4	23	0	1.3389	1
8	6	4	23	180	-1.318	2
8	6	4	23	0	0.5523	3
8	6	4	24	0	1.3389	1
8	6	4	24	180	-1.318	2
8	6	4	24	0	0.5523	3
8	12	15	16	0	-0.8452	1
8	12	15	16	180	25.104	2
8	12	15	44	180	25.104	2
9	7	5	25	0	1.3389	1
9	7	5	25	180	-1.318	2
9	7	5	25	0	0.5523	3
9	7	5	26	0	1.3389	1
9	7	5	26	180	-1.318	2
9	7	5	26	0	0.5523	3
9	18	1	52	0	-2.4393	1
9	18	1	52	180	10.6232	2
9	18	1	52	0	-1.1422	3
10	11	14	20	0	0.2134	1
10	11	14	20	180	1.4267	2

10	11	14	20	0	0.6945	3
10	11	14	42	0	1.3389	1
10	11	14	42	180	-1.318	2
10	11	14	42	0	0.5523	3
10	11	14	43	0	1.3389	1
10	11	14	43	180	-1.318	2
10	11	14	43	0	0.5523	3
10	13	17	19	0	-1.0334	1
10	13	17	19	180	0.5732	2
10	13	17	19	0	-1.318	3
10	13	17	47	0	0.1548	1
10	13	17	47	0	0.7489	3
11	10	13	17	0	-0.615	1
11	10	13	17	180	0.9163	2
11	10	13	17	0	1.2217	3
11	10	13	40	0	1.3389	1
11	10	13	40	180	-1.318	2
11	10	13	40	0	0.5523	3
11	10	13	41	0	1.3389	1
11	10	13	41	180	-1.318	2
11	10	13	41	0	0.5523	3
11	14	20	49	0	1.3389	1
11	14	20	49	180	-1.318	2
11	14	20	49	0	0.5523	3
11	14	20	50	0	1.3389	1
11	14	20	50	180	-1.318	2
11	14	20	50	0	0.5523	3
11	14	20	51	0	1.3389	1
11	14	20	51	180	-1.318	2

11	14	20	51	0	0.5523	3
12	8	6	27	0	0.6736	1
12	8	6	27	180	-0.8577	2
12	8	6	27	0	0.3012	3
12	8	6	28	0	0.6736	1
12	8	6	28	180	-0.8577	2
12	8	6	28	0	0.3012	3
12	15	16	19	0	-0.6109	1
12	15	16	19	180	0.2427	2
12	15	16	19	0	-1.0627	3
12	15	16	45	0	1.0502	1
12	15	16	45	180	-0.8577	2
12	15	16	45	0	-1.1213	3
12	15	16	46	0	1.0502	1
12	15	16	46	180	-0.8577	2
12	15	16	46	0	-1.1213	3
13	10	11	14	0	0.2134	1
13	10	11	14	180	1.4267	2
13	10	11	14	0	0.6945	3
13	10	11	37	0	1.3389	1
13	10	11	37	180	-1.318	2
13	10	11	37	0	0.5523	3
13	10	11	38	0	1.3389	1
13	10	11	38	180	-1.318	2
13	10	11	38	0	0.5523	3
13	17	19	16	0	-0.8452	1
13	17	19	16	180	25.104	2
13	17	19	48	180	25.104	2
14	11	10	35	0	1.3389	1

14	11	10	35	180	-1.318	2
14	11	10	35	0	0.5523	3
14	11	10	36	0	1.3389	1
14	11	10	36	180	-1.318	2
14	11	10	36	0	0.5523	3
15	12	8	31	0	1.0502	1
15	12	8	31	180	-0.8577	2
15	12	8	31	0	-1.1213	3
15	12	8	32	0	1.0502	1
15	12	8	32	180	-0.8577	2
15	12	8	32	0	-1.1213	3
15	16	19	17	0	-0.6109	1
15	16	19	17	180	0.2427	2
15	16	19	17	0	-1.0627	3
15	16	19	48	0	0.6276	1
15	16	19	48	180	0.2176	2
15	16	19	48	0	1.0627	3
16	15	12	39	180	25.104	2
16	19	17	47	180	25.104	2
17	13	10	35	0	0.6736	1
17	13	10	35	180	-0.8577	2
17	13	10	35	0	0.3012	3
17	13	10	36	0	0.6736	1
17	13	10	36	180	-0.8577	2
17	13	10	36	0	0.3012	3
17	19	16	45	0	1.0502	1
17	19	16	45	180	-0.8577	2
17	19	16	45	0	-1.1213	3
17	19	16	46	0	1.0502	1

17	19	16	46	180	-0.8577	2
17	19	16	46	0	-1.1213	3
18	9	7	29	0	-0.5356	1
18	9	7	29	180	0.1213	2
18	9	7	30	0	-0.5356	1
18	9	7	30	180	0.1213	2
19	16	15	44	0	0.6276	1
19	16	15	44	180	0.2176	2
19	16	15	44	0	1.0627	3
19	17	13	40	0	1.0502	1
19	17	13	40	180	-0.8577	2
19	17	13	40	0	-1.1213	3
19	17	13	41	0	1.0502	1
19	17	13	41	180	-0.8577	2
19	17	13	41	0	-1.1213	3
20	14	11	37	0	1.3389	1
20	14	11	37	180	-1.318	2
20	14	11	37	0	0.5523	3
20	14	11	38	0	1.3389	1
20	14	11	38	180	-1.318	2
20	14	11	38	0	0.5523	3
21	3	4	23	0	0.5941	1
21	3	4	23	180	-2.8995	2
21	3	4	23	0	0.6569	3
21	3	4	24	0	0.5941	1
21	3	4	24	180	-2.8995	2
21	3	4	24	0	0.6569	3
21	3	5	25	0	0.5941	1
21	3	5	25	180	-2.8995	2

21	3	5	25	0	0.6569	3
21	3	5	26	0	0.5941	1
21	3	5	26	180	-2.8995	2
21	3	5	26	0	0.6569	3
22	3	4	23	0	0.5941	1
22	3	4	23	180	-2.8995	2
22	3	4	23	0	0.6569	3
22	3	4	24	0	0.5941	1
22	3	4	24	180	-2.8995	2
22	3	4	24	0	0.6569	3
22	3	5	25	0	0.5941	1
22	3	5	25	180	-2.8995	2
22	3	5	25	0	0.6569	3
22	3	5	26	0	0.5941	1
22	3	5	26	180	-2.8995	2
22	3	5	26	0	0.6569	3
23	4	6	27	0	0.5941	1
23	4	6	27	180	-2.8995	2
23	4	6	27	0	0.6569	3
23	4	6	28	0	0.5941	1
23	4	6	28	180	-2.8995	2
23	4	6	28	0	0.6569	3
24	4	6	27	0	0.5941	1
24	4	6	27	180	-2.8995	2
24	4	6	27	0	0.6569	3
24	4	6	28	0	0.5941	1
24	4	6	28	180	-2.8995	2
24	4	6	28	0	0.6569	3
25	5	7	29	0	0.5941	1

25	5	7	29	180	-2.8995	2
25	5	7	29	0	0.6569	3
25	5	7	30	0	0.5941	1
25	5	7	30	180	-2.8995	2
25	5	7	30	0	0.6569	3
26	5	7	29	0	0.5941	1
26	5	7	29	180	-2.8995	2
26	5	7	29	0	0.6569	3
26	5	7	30	0	0.5941	1
26	5	7	30	180	-2.8995	2
26	5	7	30	0	0.6569	3
27	6	8	31	0	0.5941	1
27	6	8	31	180	-2.8995	2
27	6	8	31	0	0.6569	3
27	6	8	32	0	0.5941	1
27	6	8	32	180	-2.8995	2
27	6	8	32	0	0.6569	3
28	6	8	31	0	0.5941	1
28	6	8	31	180	-2.8995	2
28	6	8	31	0	0.6569	3
28	6	8	32	0	0.5941	1
28	6	8	32	180	-2.8995	2
28	6	8	32	0	0.6569	3
29	7	9	33	0	0.5941	1
29	7	9	33	180	-2.8995	2
29	7	9	33	0	0.6569	3
29	7	9	34	0	0.5941	1
29	7	9	34	180	-2.8995	2
29	7	9	34	0	0.6569	3

30	7	9	33	0	0.5941	1
30	7	9	33	180	-2.8995	2
30	7	9	33	0	0.6569	3
30	7	9	34	0	0.5941	1
30	7	9	34	180	-2.8995	2
30	7	9	34	0	0.6569	3
31	8	12	39	0	-1.0962	1
31	8	12	39	180	-0.477	2
31	8	12	39	0	0.4351	3
32	8	12	39	0	-1.0962	1
32	8	12	39	180	-0.477	2
32	8	12	39	0	0.4351	3
35	10	11	37	0	0.5941	1
35	10	11	37	180	-2.8995	2
35	10	11	37	0	0.6569	3
35	10	11	38	0	0.5941	1
35	10	11	38	180	-2.8995	2
35	10	11	38	0	0.6569	3
35	10	13	40	0	0.5941	1
35	10	13	40	180	-2.8995	2
35	10	13	40	0	0.6569	3
35	10	13	41	0	0.5941	1
35	10	13	41	180	-2.8995	2
35	10	13	41	0	0.6569	3
36	10	11	37	0	0.5941	1
36	10	11	37	180	-2.8995	2
36	10	11	37	0	0.6569	3
36	10	11	38	0	0.5941	1
36	10	11	38	180	-2.8995	2

36	10	11	38	0	0.6569	3
36	10	13	40	0	0.5941	1
36	10	13	40	180	-2.8995	2
36	10	13	40	0	0.6569	3
36	10	13	41	0	0.5941	1
36	10	13	41	180	-2.8995	2
36	10	13	41	0	0.6569	3
37	11	14	42	0	0.5941	1
37	11	14	42	180	-2.8995	2
37	11	14	42	0	0.6569	3
37	11	14	43	0	0.5941	1
37	11	14	43	180	-2.8995	2
37	11	14	43	0	0.6569	3
38	11	14	42	0	0.5941	1
38	11	14	42	180	-2.8995	2
38	11	14	42	0	0.6569	3
38	11	14	43	0	0.5941	1
38	11	14	43	180	-2.8995	2
38	11	14	43	0	0.6569	3
39	12	15	44	180	25.104	2
40	13	17	47	0	-1.0962	1
40	13	17	47	180	-0.477	2
40	13	17	47	0	0.4351	3
41	13	17	47	0	-1.0962	1
41	13	17	47	180	-0.477	2
41	13	17	47	0	0.4351	3
42	14	20	49	0	0.5941	1
42	14	20	49	180	-2.8995	2
42	14	20	49	0	0.6569	3

42	14	20	50	0	0.5941	1
42	14	20	50	180	-2.8995	2
42	14	20	50	0	0.6569	3
42	14	20	51	0	0.5941	1
42	14	20	51	180	-2.8995	2
42	14	20	51	0	0.6569	3
43	14	20	49	0	0.5941	1
43	14	20	49	180	-2.8995	2
43	14	20	49	0	0.6569	3
43	14	20	50	0	0.5941	1
43	14	20	50	180	-2.8995	2
43	14	20	50	0	0.6569	3
43	14	20	51	0	0.5941	1
43	14	20	51	180	-2.8995	2
43	14	20	51	0	0.6569	3
44	15	16	45	0	-1.0962	1
44	15	16	45	180	-0.477	2
44	15	16	45	0	0.4351	3
44	15	16	46	0	-1.0962	1
44	15	16	46	180	-0.477	2
44	15	16	46	0	0.4351	3
45	16	19	48	0	-1.0962	1
45	16	19	48	180	-0.477	2
45	16	19	48	0	0.4351	3
46	16	19	48	0	-1.0962	1
46	16	19	48	180	-0.477	2
46	16	19	48	0	0.4351	3
47	17	19	48	180	25.104	2

Improper

**Atom Numbers**				**ϕ_0 / deg**	**k_ϕ / kJ mol^{-1}**
18	9	1	2	0	84.9101
9	7	18	33	0	0
9	7	18	34	0	0
7	5	9	29	0	0
7	5	9	30	0	0
5	3	7	25	0	0
5	3	7	26	0	0
3	4	5	21	0	0
3	4	5	22	0	0
4	6	3	23	0	0
4	6	3	24	0	0
6	8	4	27	0	0
6	8	4	28	0	0
8	12	6	31	0	0
8	12	6	32	0	0
12	15	8	39	0	7.8324
15	16	12	44	0	7.8324
16	19	15	45	0	0
16	19	15	46	0	0
19	17	16	48	0	7.8324
17	13	19	47	0	7.8324
13	10	17	40	0	0
13	10	17	41	0	0
10	11	13	35	0	0
10	11	13	36	0	0
11	14	10	37	0	0
11	14	10	38	0	0

14	20	11	42	0	0
14	20	11	43	0	0
20	49	14	50	0	0
20	49	14	51	0	0

CO2

q	**σ_{ii} / Å**	**ε_{ii} / kJ mol^{-1}**	**#**
0.65120	2.75700	0.23390	1
-0.32560	3.03300	0.66950	2
-0.32560	3.03300	0.66950	3

Bonds

**Atom Numbers**		**r_{eq} /Å**	**k_r/ kJ mol^{-1} Å^{-2}**
1	2	1.160	4309.5
1	3	1.160	4309.5

Angles

**Atom Numbers**			**θ_{eq} / deg**	**k_θ / kJ mol^{-1} rad^{-2}**
2	1	3	180	234.3

N_2

q	**σ_{ii} / Å**	**ε_{ii} / kJ mol^{-1}**	**#**
0.00000	3.32000	0.30300	1
0.00000	3.32000	0.30300	2

Bonds

**Atom Numbers**		**r_{eq} /Å**	**k_r/ kJ mol^{-1} Å^{-2}**
1	2	1.100	2764.56

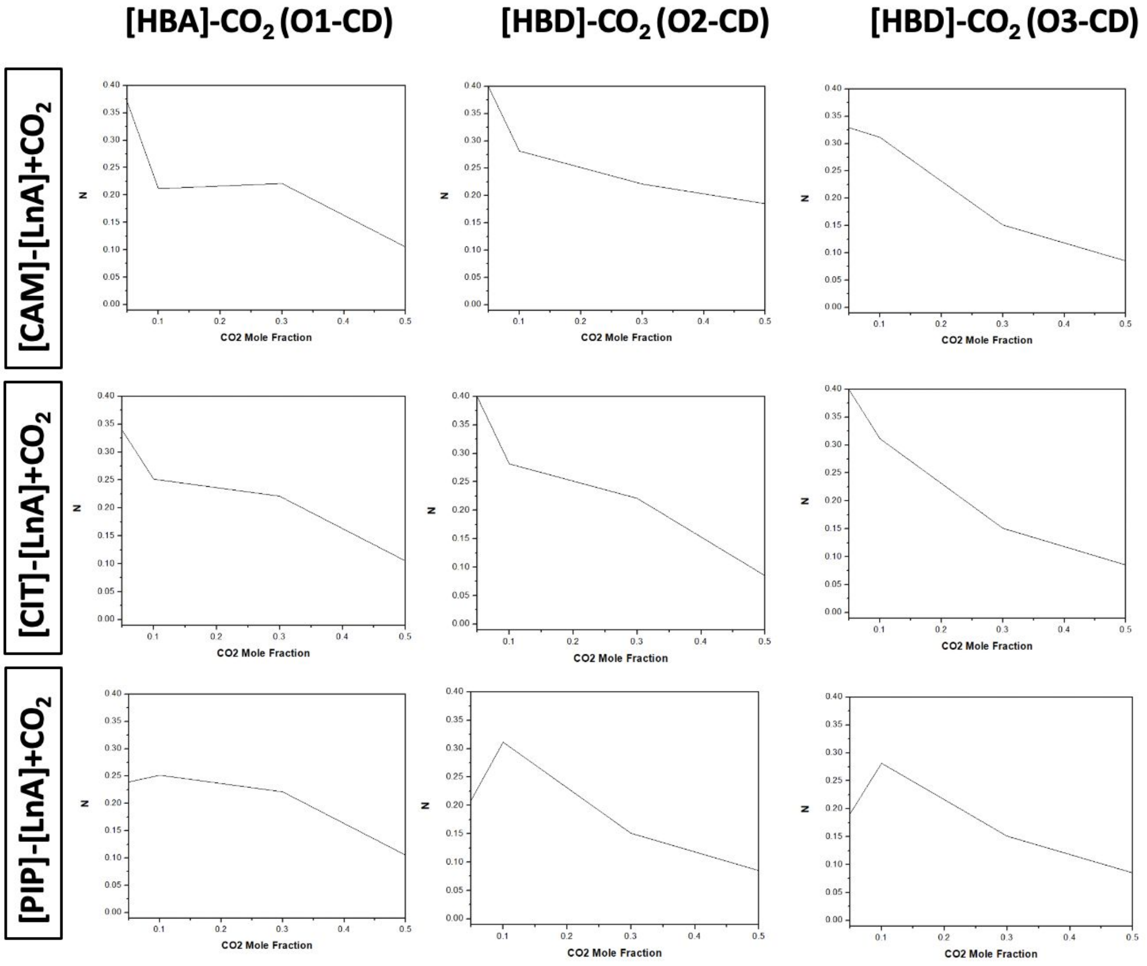

[HBA]-CO2 (O1-CD)
[HBD]-CO2 (O2-CD)
[HBD]-CO2 (O3-CD)
[CAM]-[LnA]+CO2
[CIT]-[LnA]+CO2
[PIP]-[LnA]+CO2
N
CO2 Mole Fraction

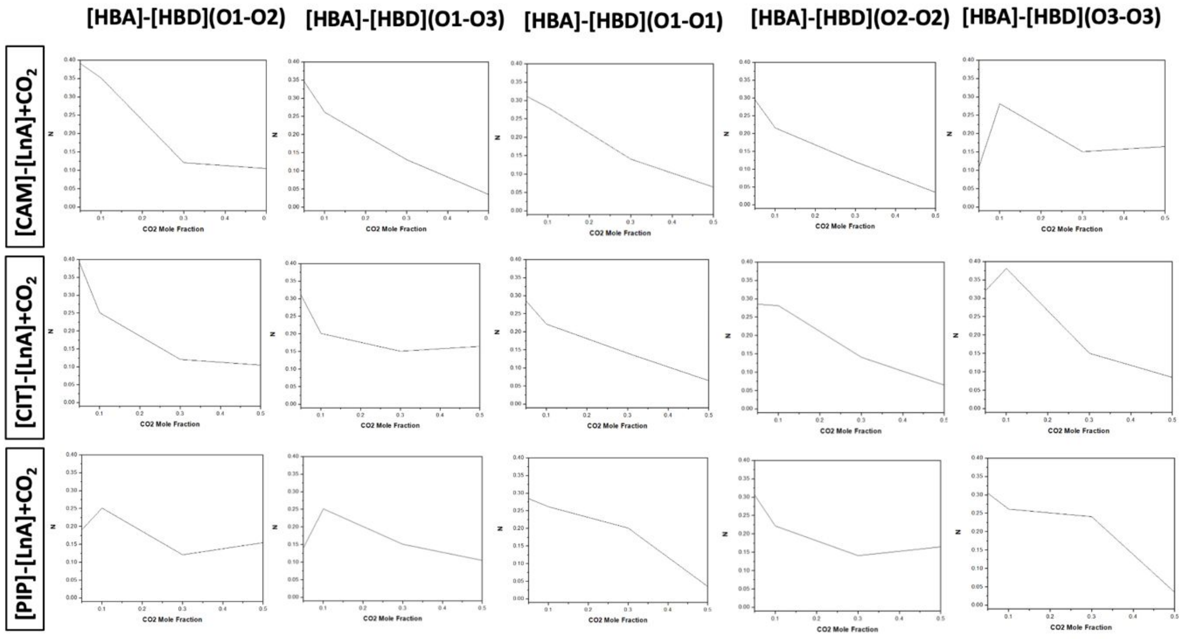

[HBA]-[HBD](O1-O2)
[HBA]-[HBD](O1-O3)
[HBA]-[HBD](O1-O1)
[HBA]-[HBD](O2-O2)
[HBA]-[HBD](O3-O3)
[CAM]-[LnA]+CO2
[CIT]-[LnA]+CO2
[PIP]-[LnA]+CO2
N
CO2 Mole Fraction

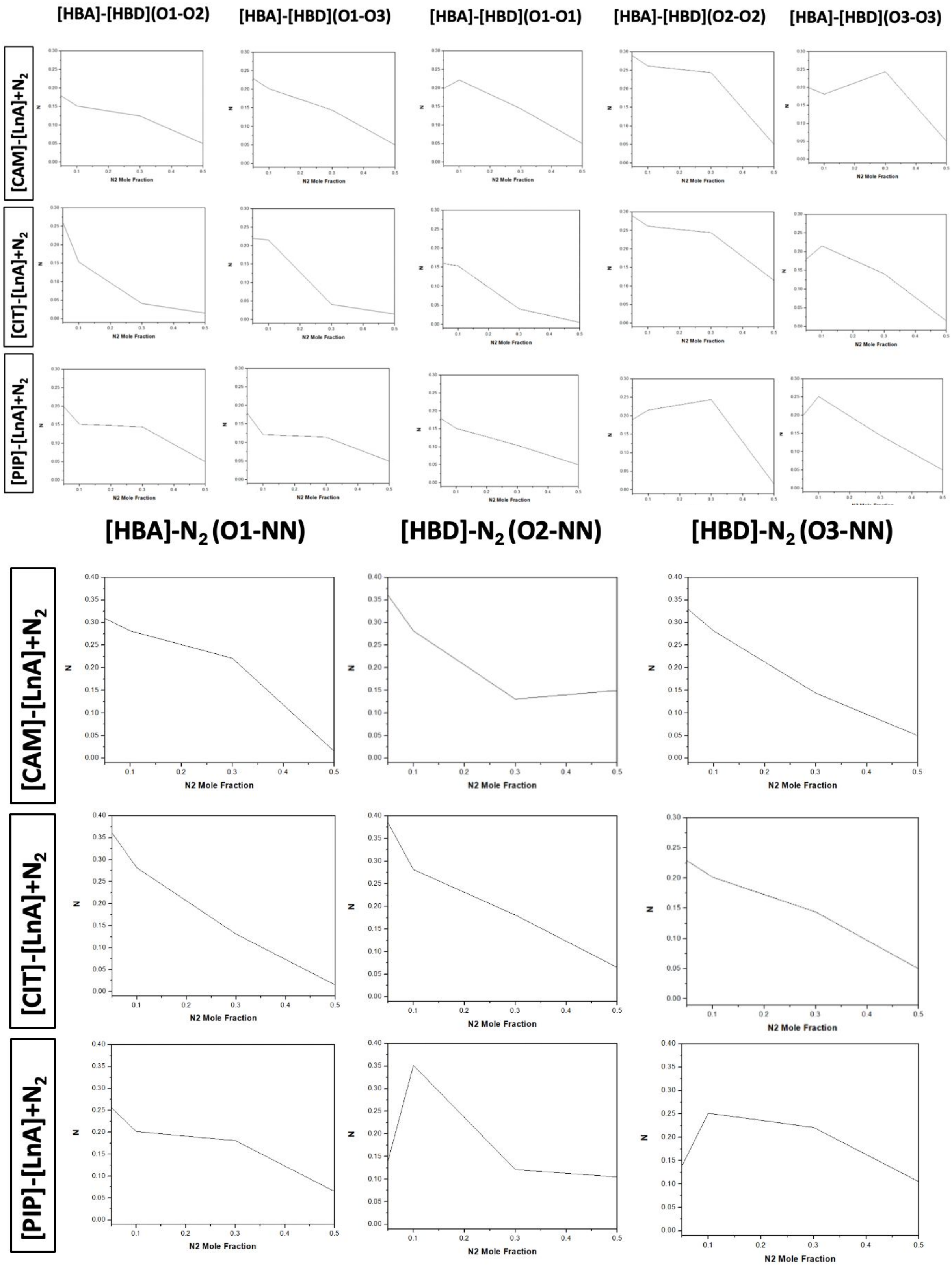

Figure S10. The solvation numbers (*N*) for [HBAs]:CO_2/N_2 and [HBD]:CO_2/N_2 sites in the reported NADES (1:1) + CO_2/N_2 as a function of x_{CO2} (CO_2 effect) and x_{N2} (N_2 effect).
